# Supplementary material for: The Cell Tracking Challenge: 10 years of objective benchmarking
Source: Nat Methods. 2023 May 18;20(7):1010–20. doi: 10.1038/s41592-023-01879-y (PMC10333123; doi:10.1038/s41592-023-01879-y)
Supplement: Supplementary file 1 — Supplementary Table 1 and Figs. 1–56 [file 41592_2023_1879_MOESM1_ESM.pdf]

nature methods

Analysis

https://doi.org/10.1038/s41592-023-01879-y

The Cell Tracking Challenge: 10 years of objective benchmarking

In the format provided by the authors and unedited

Supplementary Materials: Tables

Name	Objects of interest	Modality/Magnification /Bit depth	Frame size [grid points]	No. of frames	Spatial resolution [µm]	Temporal resolution [min]	No. of tracks	No. of cell instances
**BF-C2DL-HSC**	Mouse hematopoietic stem cells in microwells	BF/10×/8	1010×1010	1764	0.645×0.645	5	326 (820)	57915 (128494)
**BF-C2DL-MuSC**	Mouse muscle stem cells in microwells	BF/10×/8	1070×1036	1376	0.645×0.645	5	86 (47)	8753 (6372)
**DIC-C2DH-HeLa**	HeLa cells	DIC/63×/8	512×512	115	0.19×0.19	10	17 (37)	1366 (1157)
**Fluo-C2DL-Huh7**	Human hepatocarcinoma cells	WF/20×/8	1024×1024	30	0.65×0.65	15	31 (111)	781 (2783)
**Fluo-C2DL-MSC**	Rat mesenchymal stem cells	SDC/20×/16	992×832 (1200×782)	48	0.3×0.3 (0.398×0.398)	20 (30)	20 (15)	657 (258)
**Fluo-N2DH-GOWT1**	Nuclei of GOWT1 mouse stem cells	LSC/63×/8	1024×1024	92	0.24×0.24	5	56 (51)	3014 (2418)
**Fluo-N2DL-HeLa**	Nuclei of HeLa cells	WF/10×/16	1100×700	92	0.645×0.645	30	400 (406)	12836 (16538)
**PhC-C2DH-U373**	Glioblastoma-astrocytoma U373 cells	PhC/20×/8	696×520	115	0.65×0.65	15	7 (11)	531 (679)
**PhC-C2DL-PSC**	Pancreatic stem cells	PhC/4×/8	720×576	300	1.6×1.6	10	1299 (1404)	70268 (76372)
**Fluo-N2DH-SIM+**	Synthetic nuclei of HL60 cells	WF/40×/16	660×718 (664×790)	110 (138)	0.12×0.12	28.8	155 (141)	5463 (4767)
**Fluo-C3DH-A549**	A549 lung cancer cells embedded in Matrigel	SDC/63×/16	350×300×29 (300×400×34)	30	0.126×0.126×1	2	1	30
**Fluo-C3DH-H157**	H157 lung cancer cells	SDC/63×/16	992×832×35 (992×832×80)	60	0.12×0.12×0.5	1 (2)	6 (10)	347 (143)
**Fluo-C3DL-MDA231**	MDA231 human breast carcinoma cells	LSC/20×/16	512×512×30	12	1.24×1.24×6	80	46 (43)	452 (394)
**Fluo-N3DH-CE**	Early *C. elegans* developing embryo	LSC/63×/8	712×512×31	190 (140)	0.09×0.09×1	1	370 (718)	10823 (16227)
**Fluo-N3DH-CHO**	Nuclei of Chinese hamster ovarian cells	LSC/63×/8	512×443×5	92	0.2×0.2×1	9.5	27 (30)	1008 (985)

**Fluo-N3DL-DRO**	Developing *Drosophila melanogaster* embryo	LSh/16×/16	1272×603×125	50	0.4×0.4×2.03	0.5	193 (415)	9650 (11962)
**Fluo-N3DL-TRIC**	Developing *Tribolium castaneum* embryo (3D cartographic projection)	LSh/20×/16	1745×2440×13 (1693×2454×13)	100 (170)	N/A	1.5	266 (263)	14830 (32105)
**Fluo-N3DL-TRIF**	Developing *Tribolium castaneum* embryo	LSh/20×/16	1000×1820×975 (965×1861×991)	134 (77)	0.38×0.38×0.38	1.5	466 (261)	42483 (8744)
**Fluo-C3DH-A549-SIM**	Simulated A549 lung cancer cells embedded in Matrigel	SDC/63×/16	350×300×29 (400×300×40)	30	0.126×0.126×1	0.33	1	30
**Fluo-N3DH-SIM+**	Synthetic nuclei of HL60 cells	SDC/40×/16	720×728×59 (702×716×59)	150 (110)	0.12×0.12×0.2	28.8	136 (161)	4535 (5410)

Supplementary Table 1. Technical features of the test datasets. **Note**: brightfield (BF); spinning-disk confocal (SDC); laser-scanning confocal (LSC); widefield fluorescence (WF); phase contrast (PhC); differential interference contrast (DIC); light-sheet (LSh). The numbers in parentheses indicate particular values for the second video in a given dataset.

Supplementary Materials: Figures

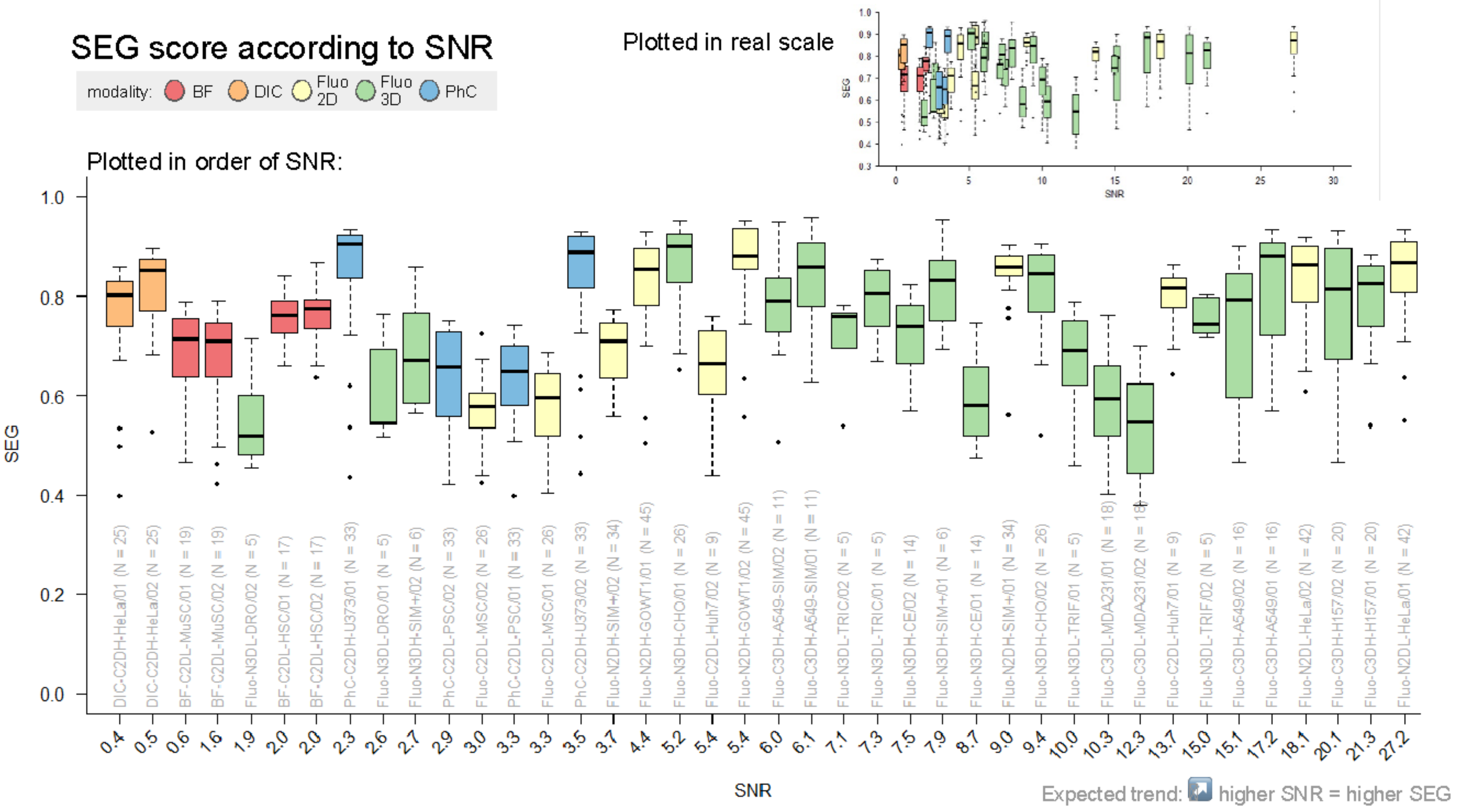

Supplementary Figure 1. Segmentation scores as a function of Signal-to-noise ratio (SNR). Bold line represents median values. Measurements given per video sequence. Outliers indicate values higher/lower than 1.5 times the interquartile range.

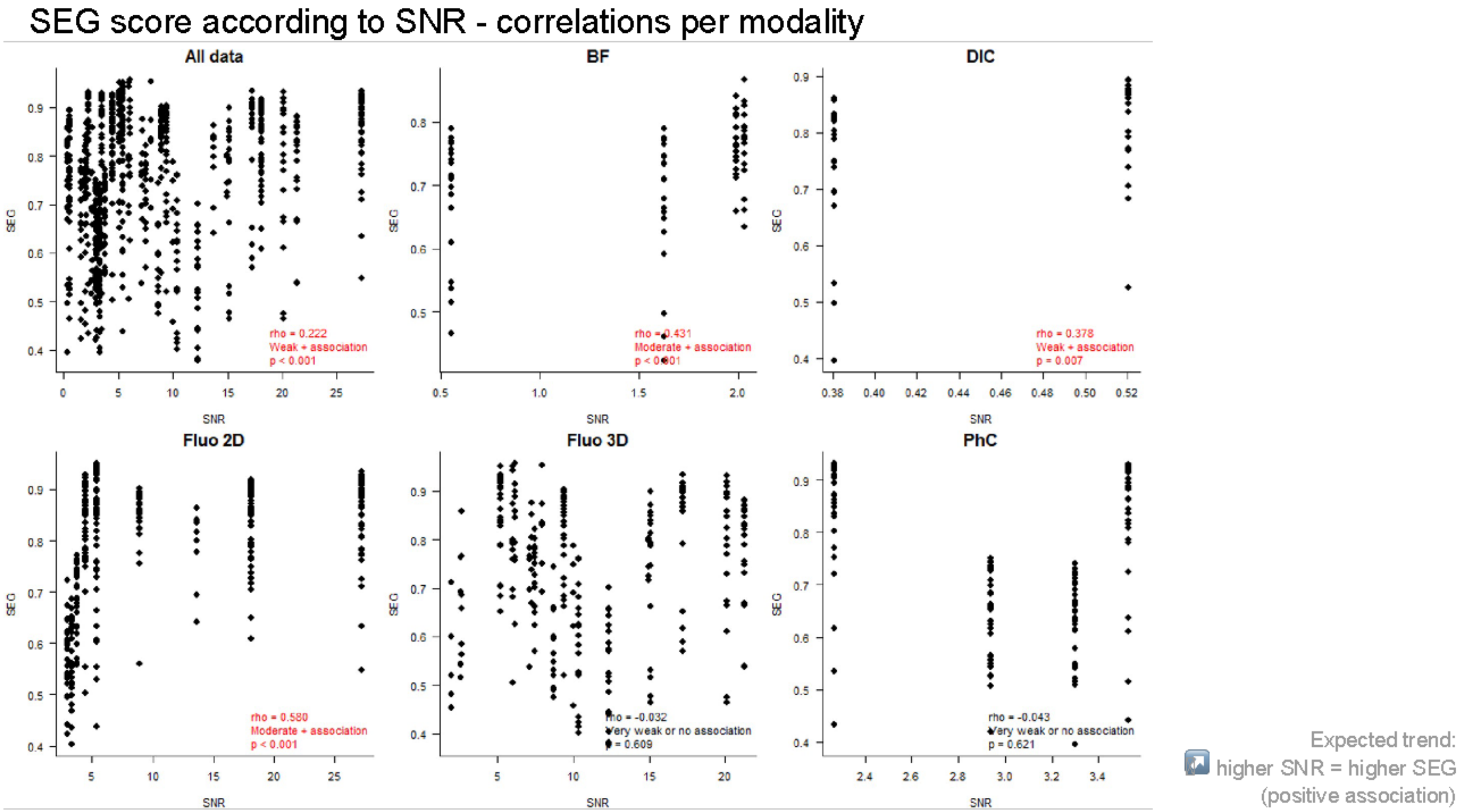

Supplementary Figure 2. Spearman's rank correlation coefficient between Segmentation scores and Signal-to-noise ratio (SNR). Significance level fixed at 0.05. Measurements given per video sequence.

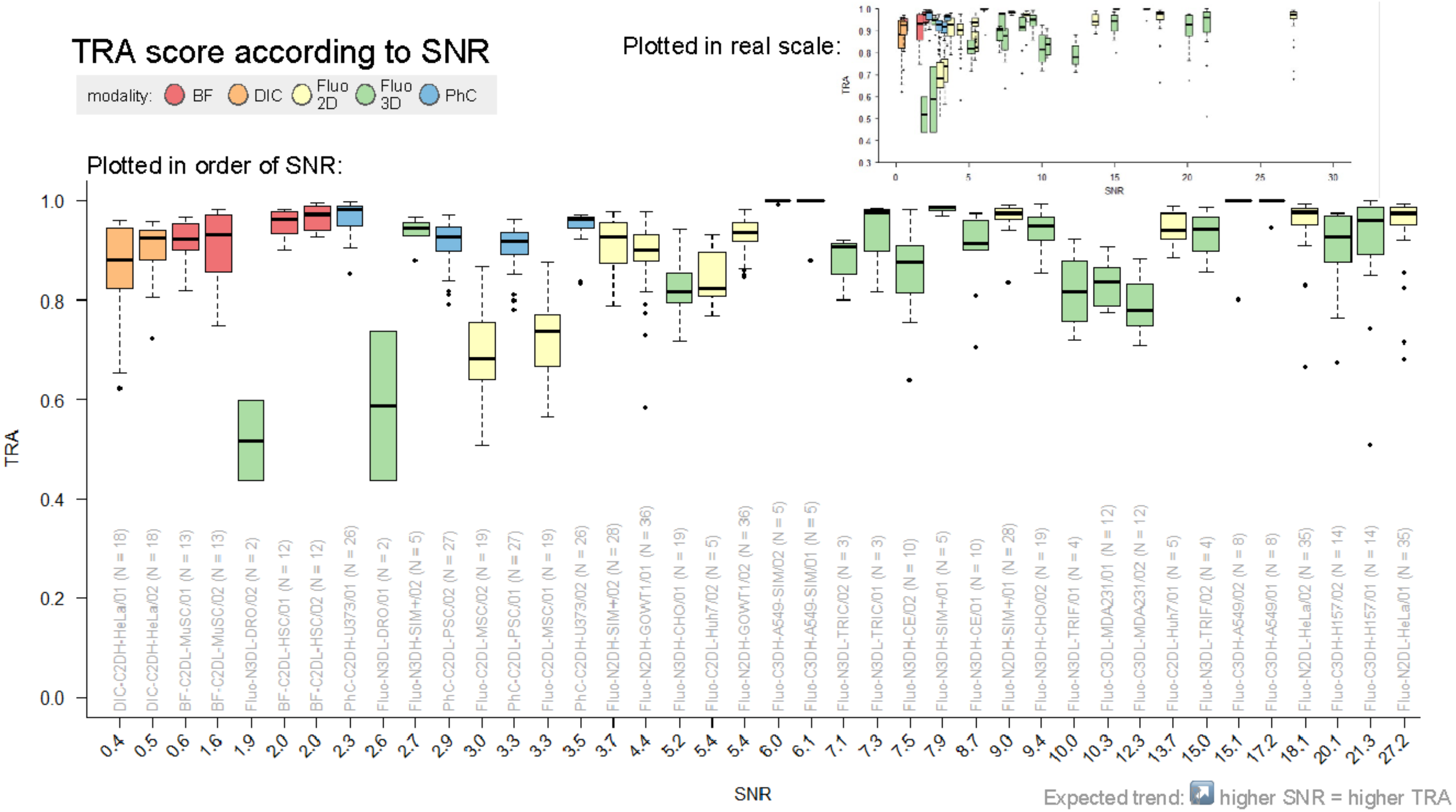

Supplementary Figure 3. Tracking scores as a function of Signal-to-noise ratio (SNR). Bold line represents median values. Measurements are given per video sequence. Outliers indicate values higher/lower than 1.5 times the interquartile range

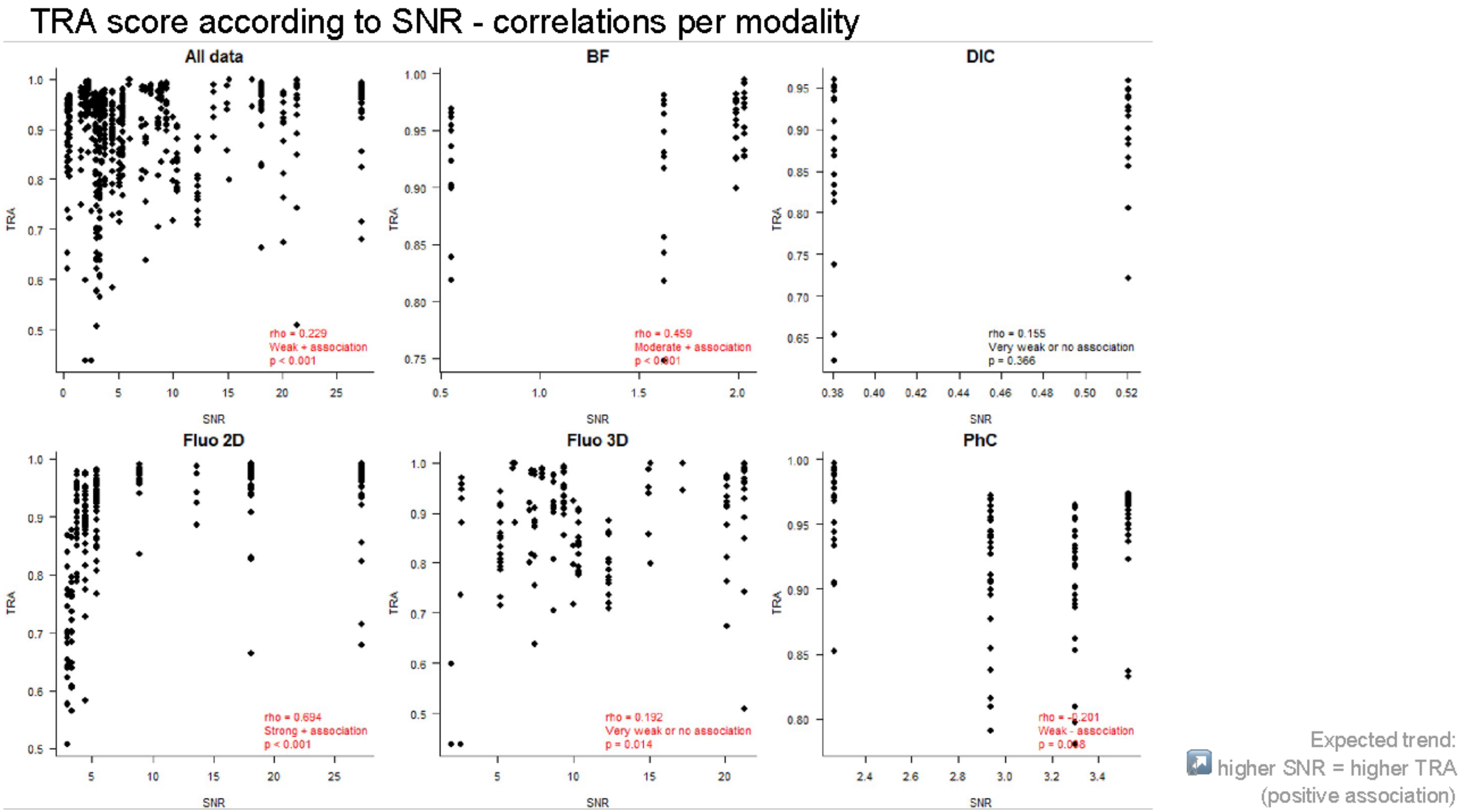

Supplementary Figure 4. Spearman's rank correlation coefficient between Tracking scores and Signal-to-noise ratio (SNR). Significance level fixed at 0.05. Measurements given per video sequence.

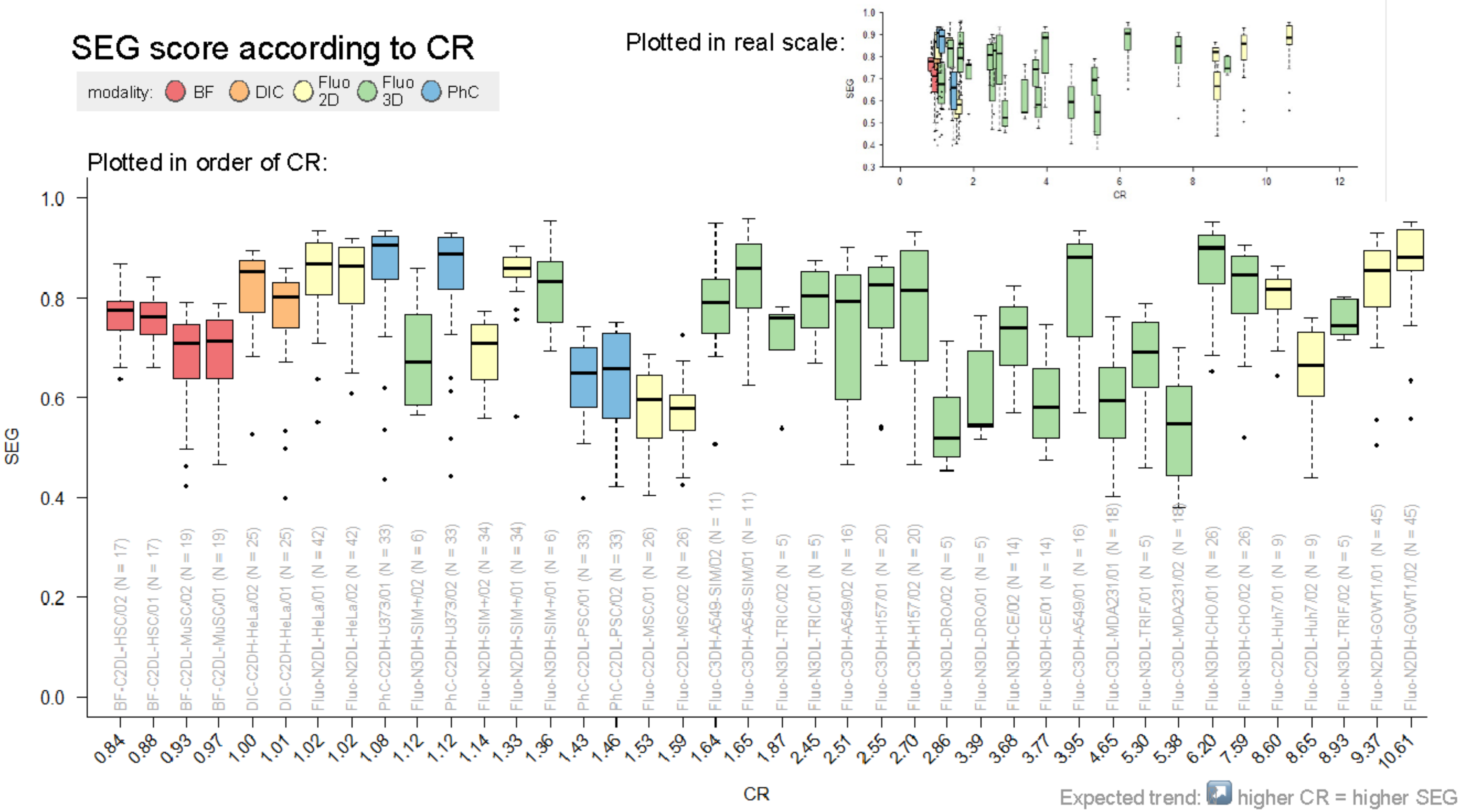

Supplementary Figure 5. Segmentation scores as a function of Contrast Ratio (CR). Bold line represents median values. Measurements are given per video sequence. Outliers indicate values higher/lower than 1.5 times the interquartile range.

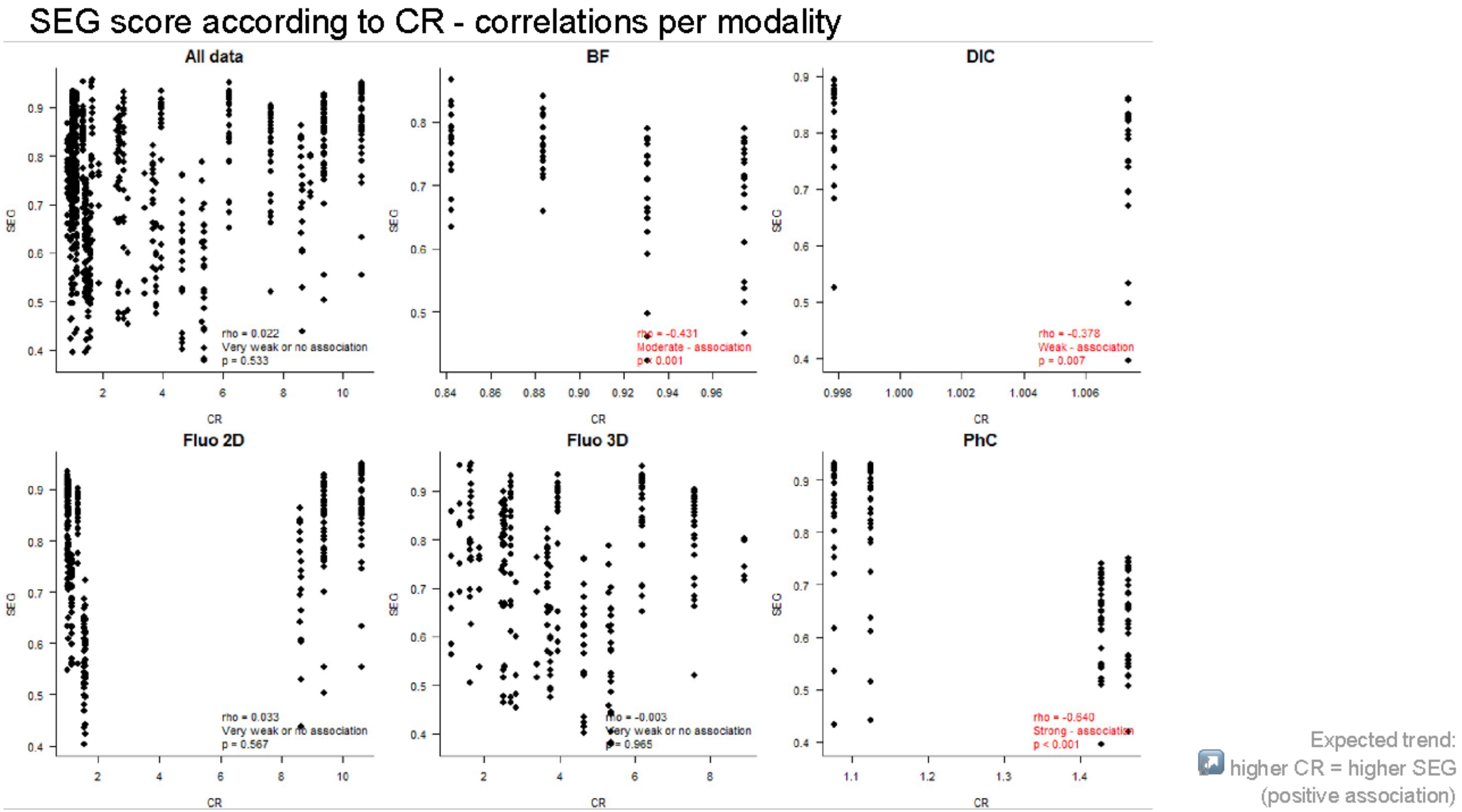

Supplementary Figure 6. Spearman's rank correlation coefficient between Segmentation scores and Contrast Ratio (CR). Significance level fixed at 0.05. Measurements given per video sequence.

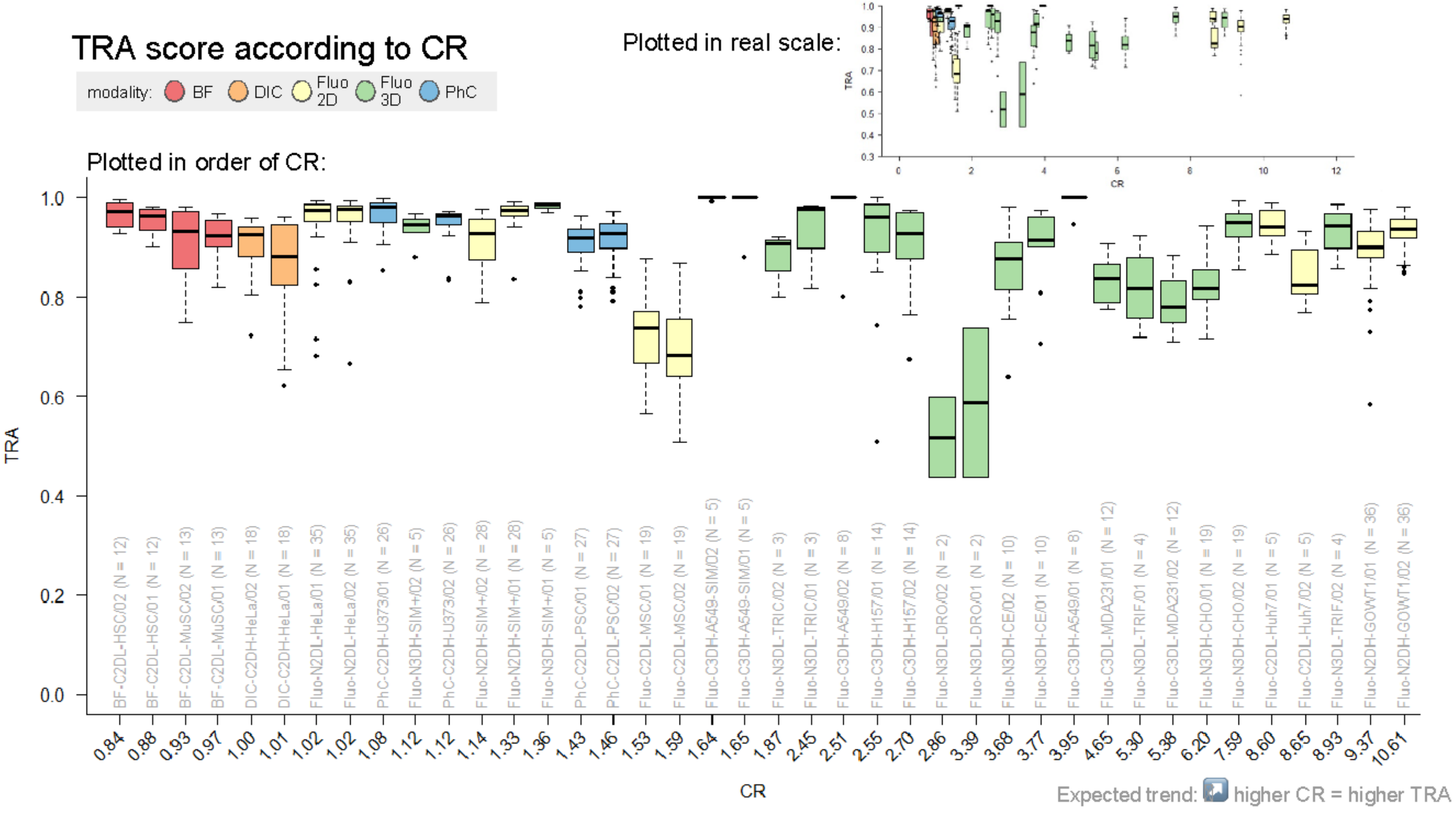

Supplementary Figure 7. Tracking scores as a function of Contrast Ratio (CR). Bold line represents median values. Measurements are given per video sequence. Outliers indicate values higher/lower than 1.5 times the interquartile range.

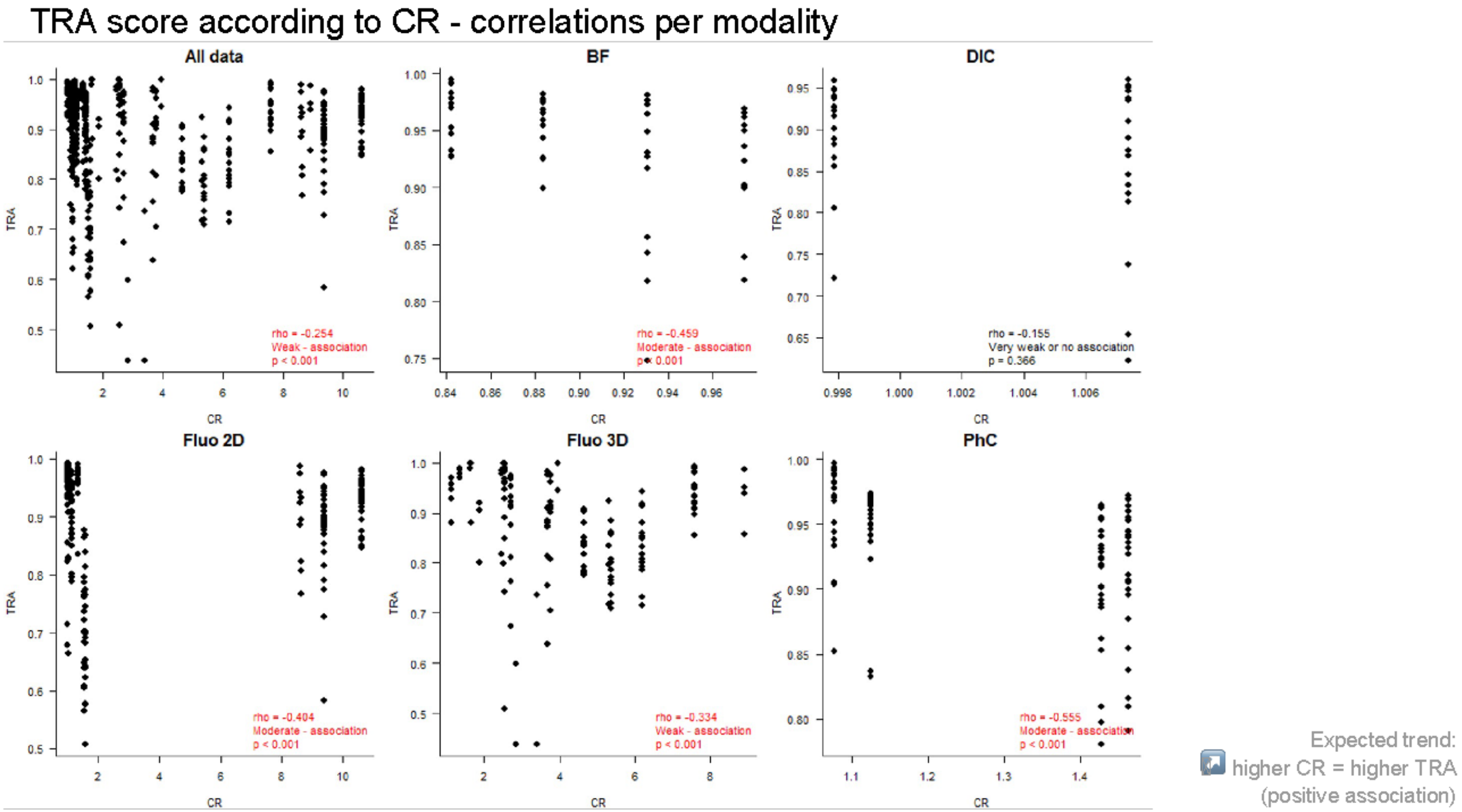

Supplementary Figure 8. Supplementary Figure 6. Spearman's rank correlation coefficient between Tracking scores and Contrast Ratio (CR). Significance level fixed at 0.05. Measurements given per video sequence.

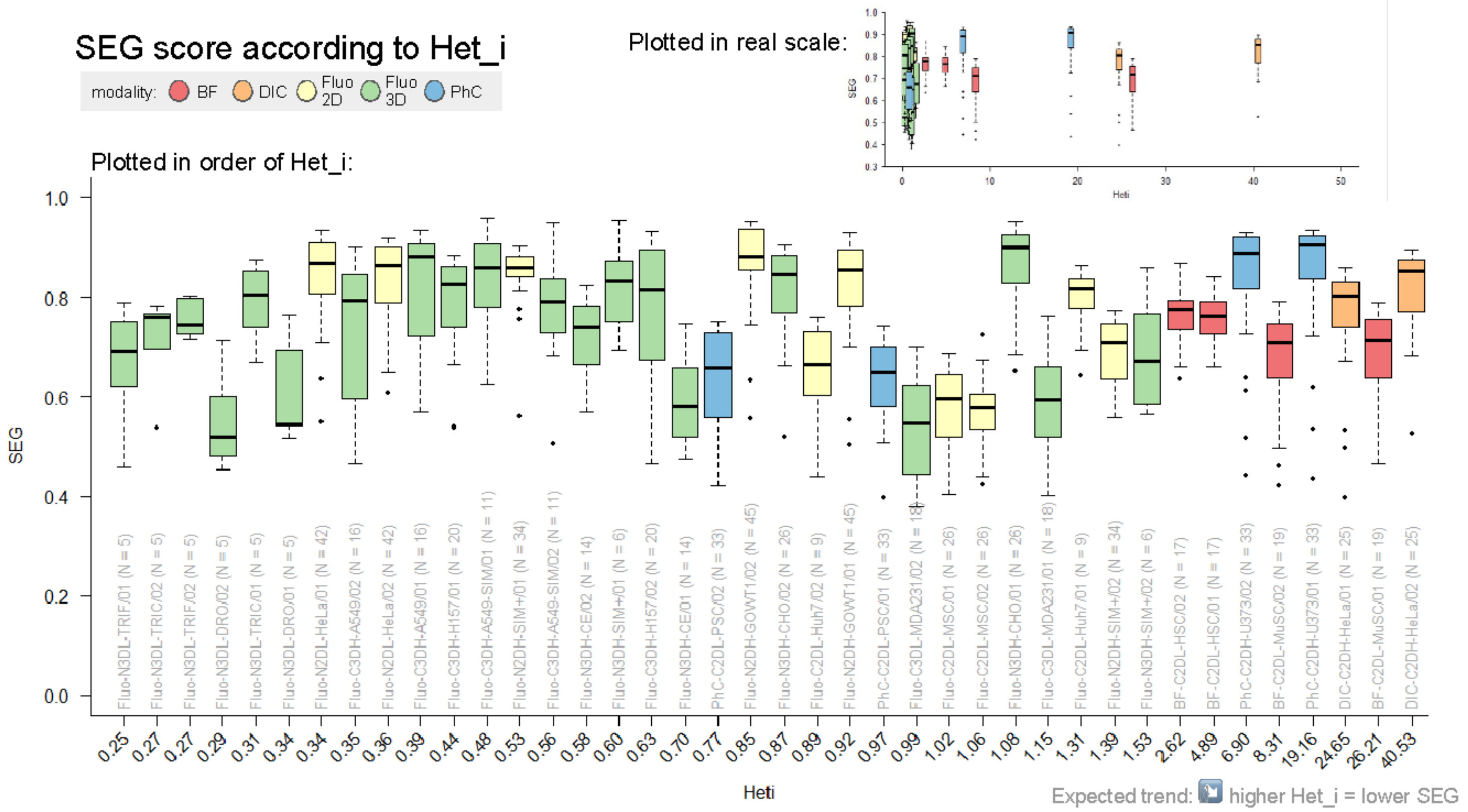

Supplementary Figure 9. Segmentation scores as a function of Heterogeneity of the signal inside the cells (Het_i) Bold line represents median values. Measurements are given per video sequence. Outliers indicate values higher/lower than 1.5 times the interquartile range**.**

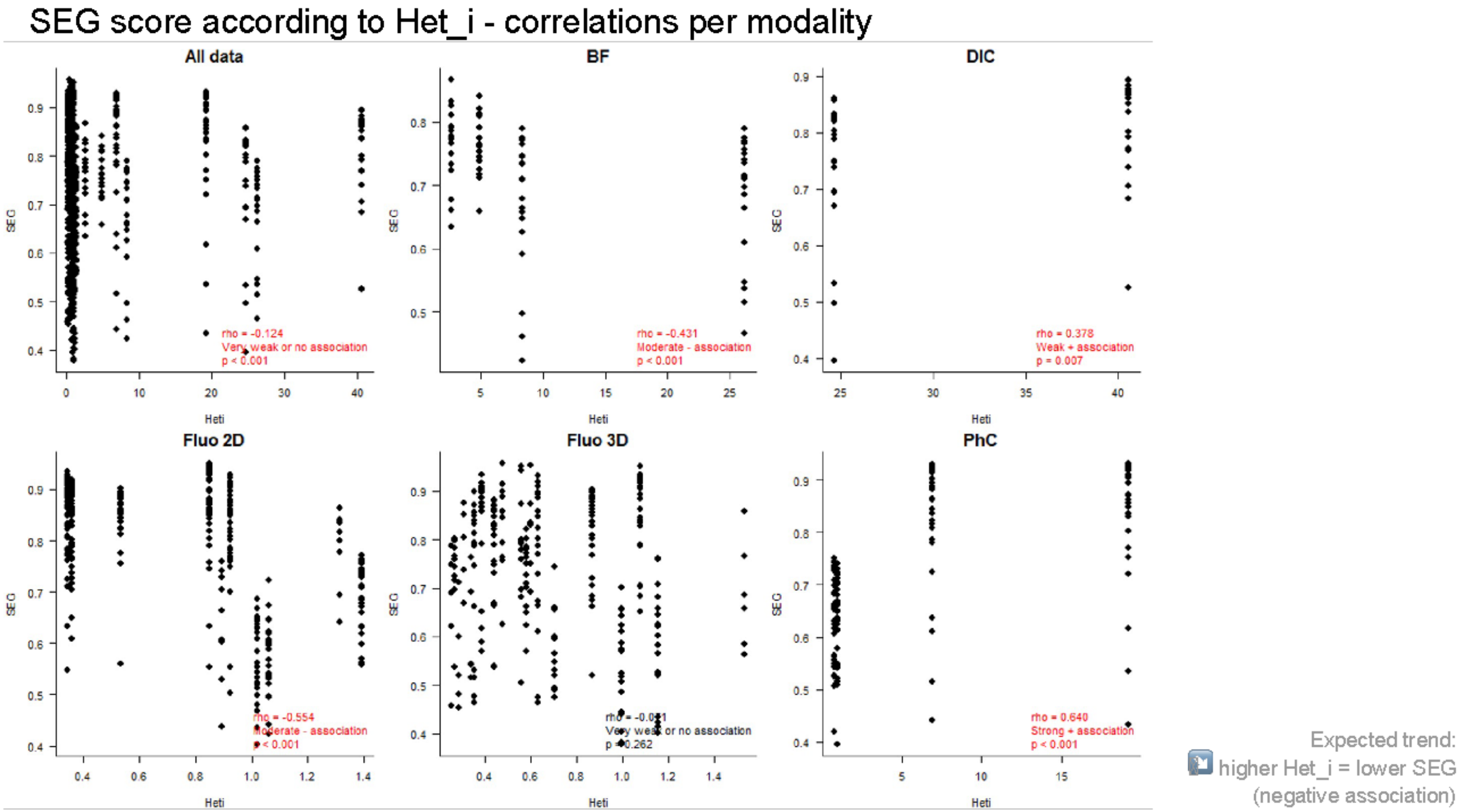

Supplementary Figure 10. Spearman's rank correlation coefficient between Segmentation scores and Heterogeneity of the signal inside the cells (Het_i). Significance level fixed at 0.05. Measurements given per video sequence.

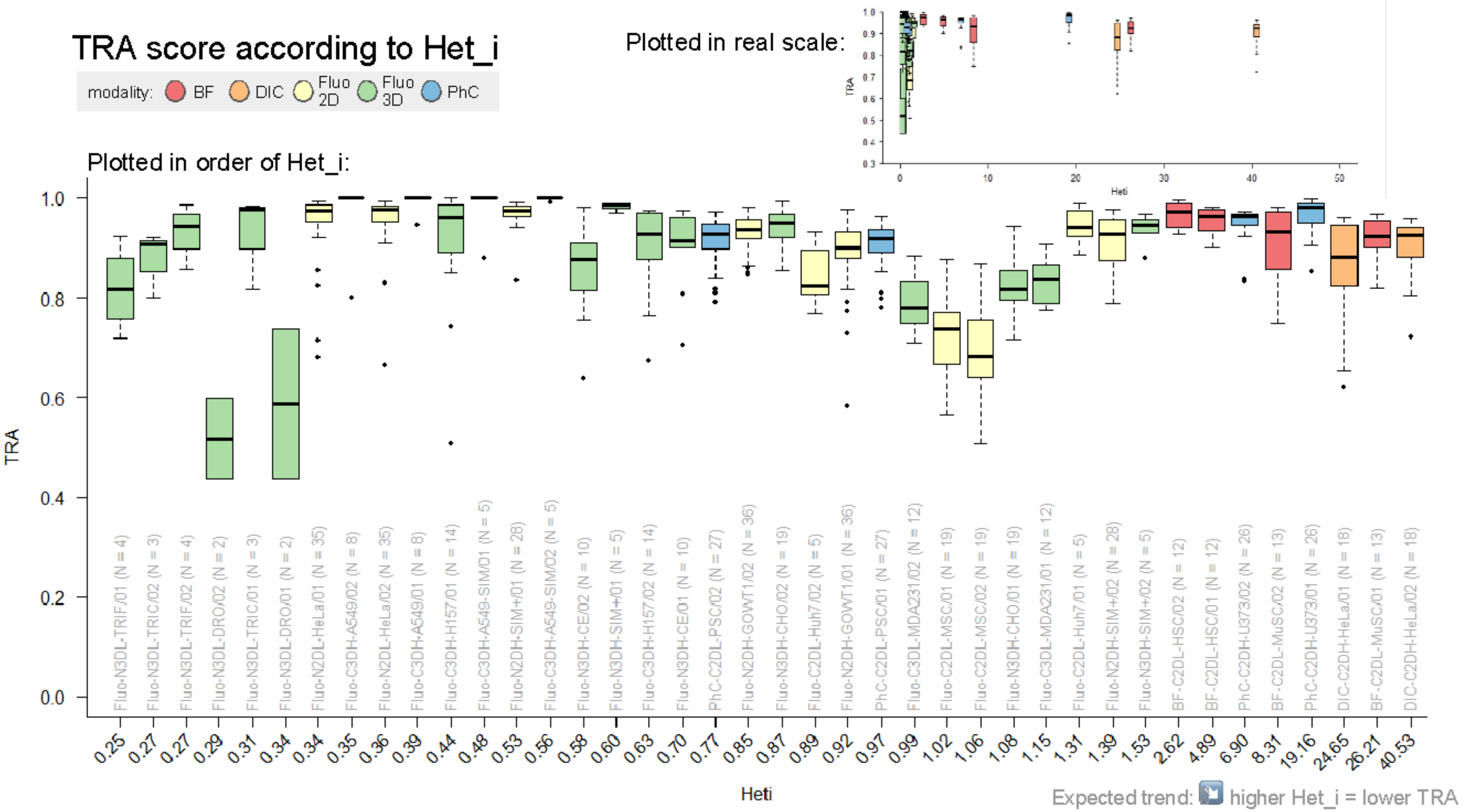

Supplementary Figure 11. Tracking scores as a function of Heterogeneity of the signal inside the cells (Het_i) Bold line represents median values. Measurements are given per video sequence. Outliers indicate values higher/lower than 1.5 times the interquartile range

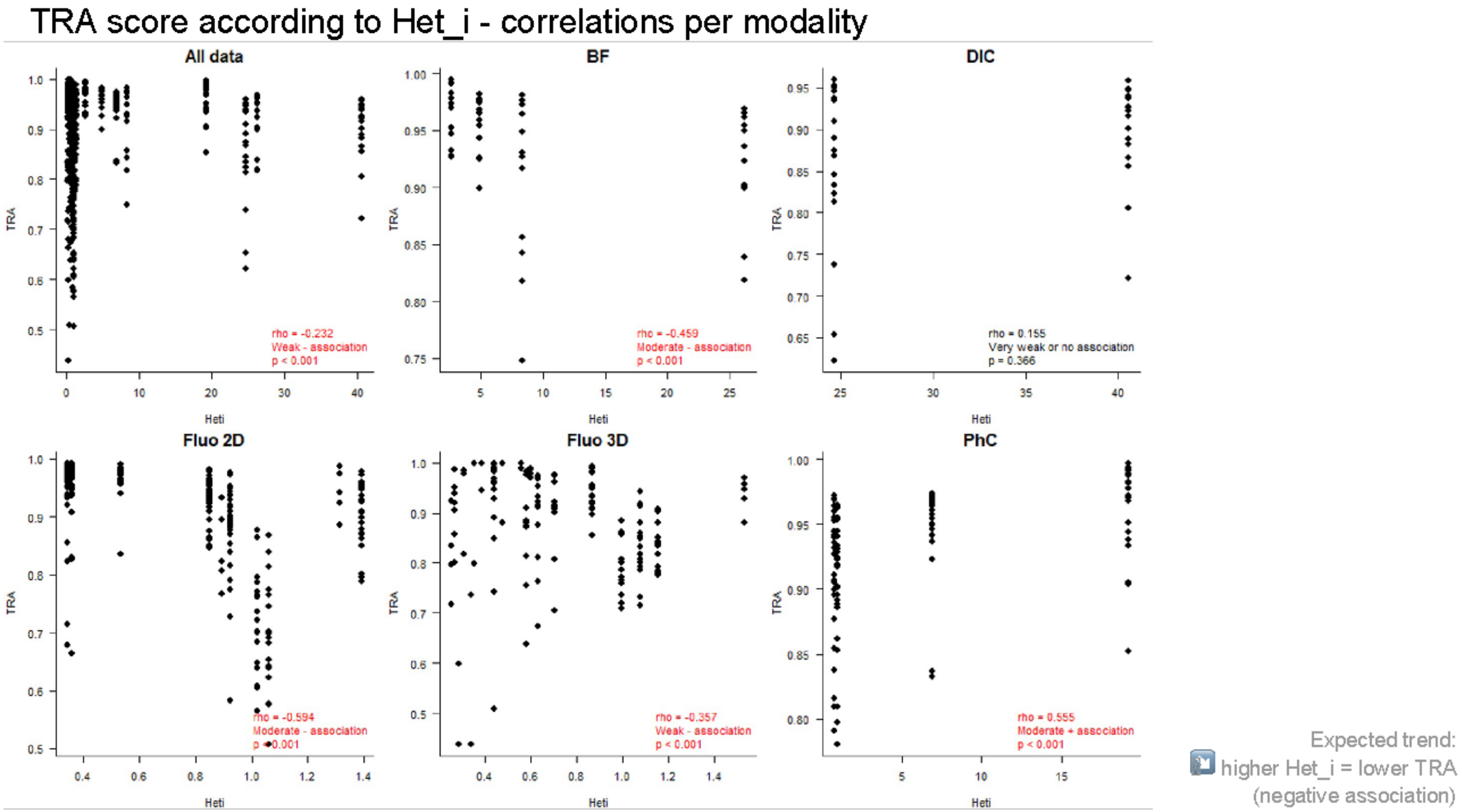

Supplementary Figure 12. Spearman's rank correlation coefficient between Tracking scores and Heterogeneity of the signal inside the cells (Het_i). Significance level fixed at 0.05. Measurements given per video sequence.

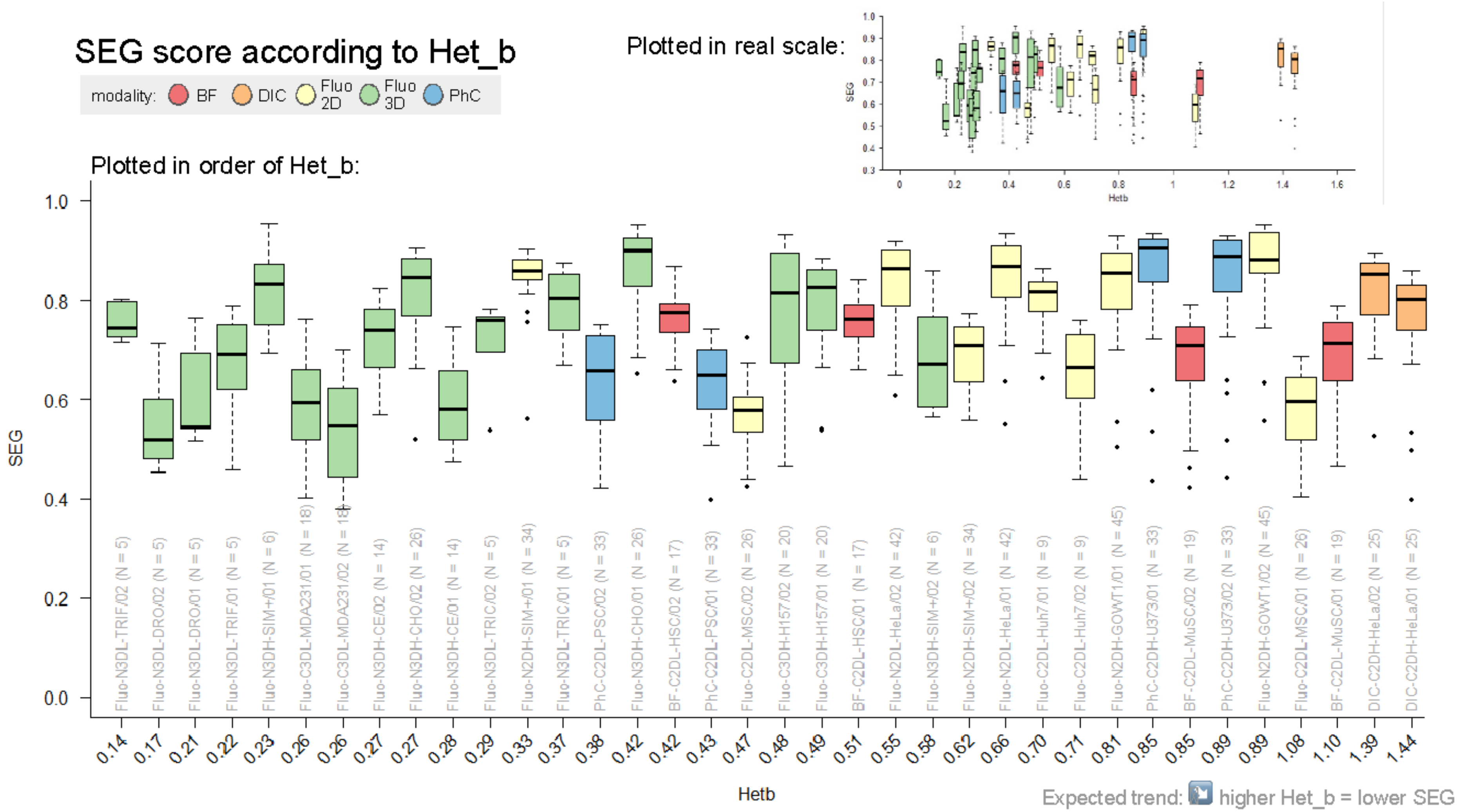

Supplementary Figure 13. Segmentation scores as a function of Heterogeneity of the signal between the cells (Het_b) Bold line represents median values. Measurements are given per video sequence. Outliers indicate values higher/lower than 1.5 times the interquartile range.

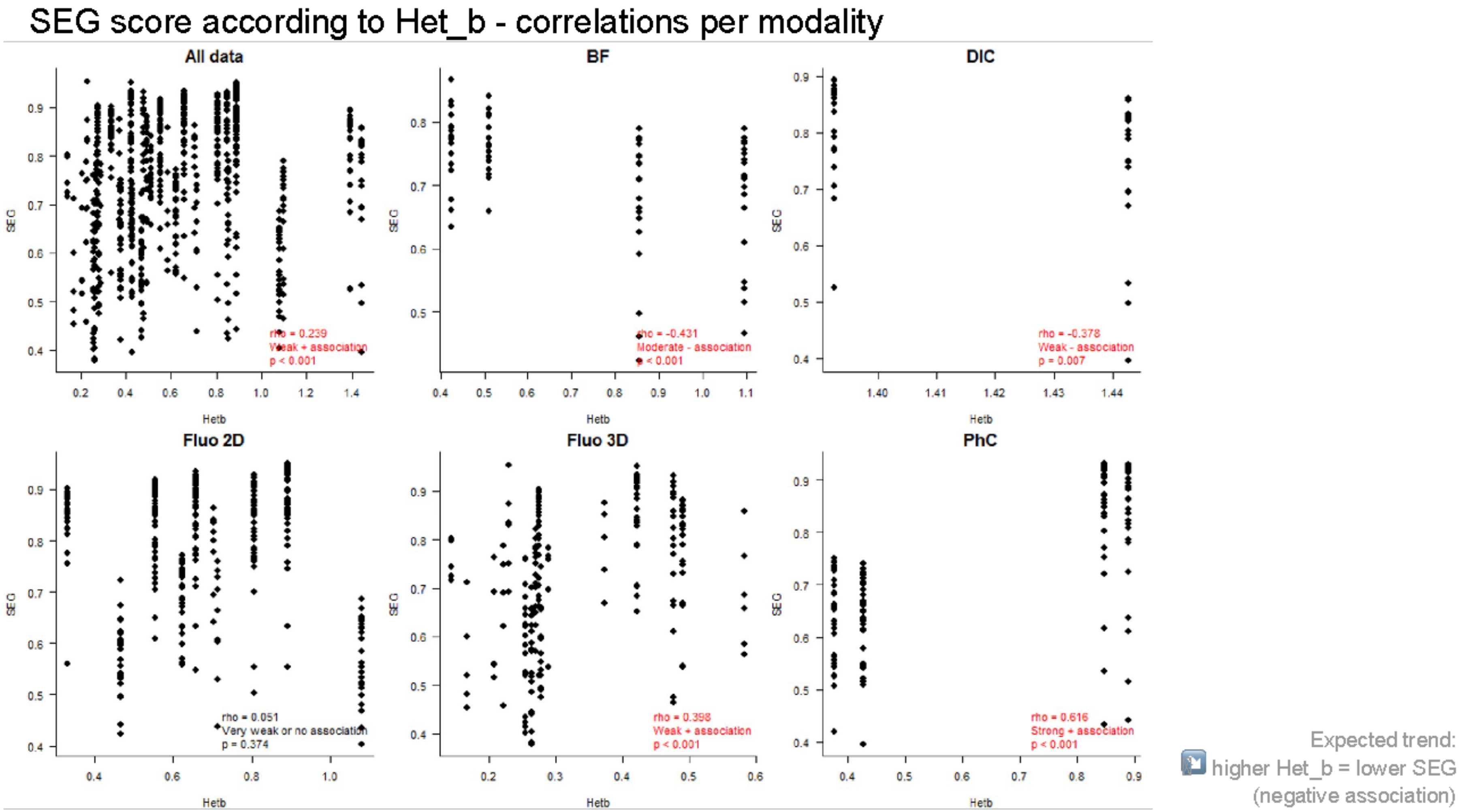

Supplementary Figure 14. Spearman's rank correlation coefficient between Segmentation scores and Heterogeneity of the signal between the cells (Het_b). Significance level fixed at 0.05. Measurements given per video sequence.

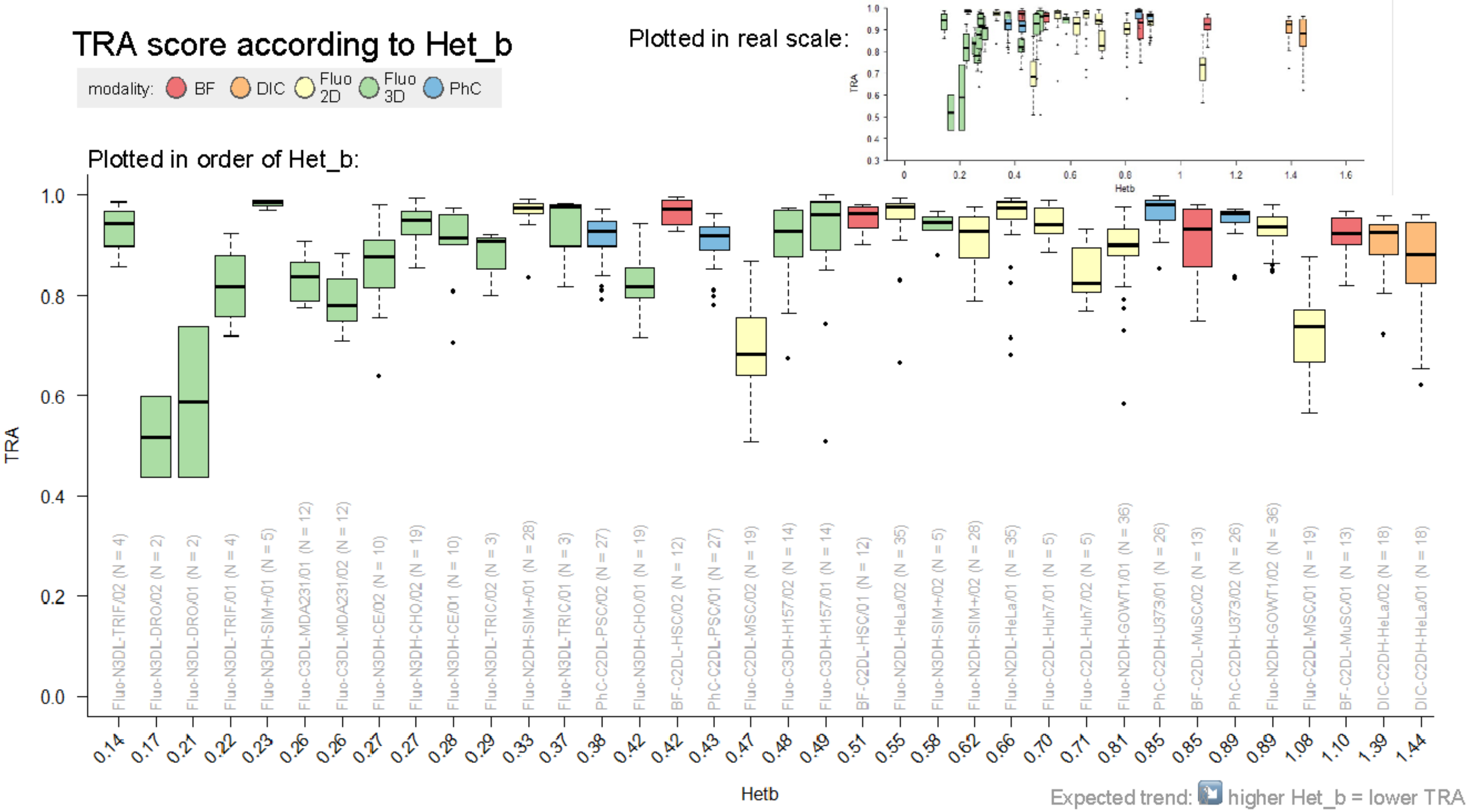

Supplementary Figure 15. Tracking scores as a function of Heterogeneity of the signal between the cells (Het_b) Bold line represents median values. Measurements are given per video sequence. Outliers indicate values higher/lower than 1.5 times the interquartile range.

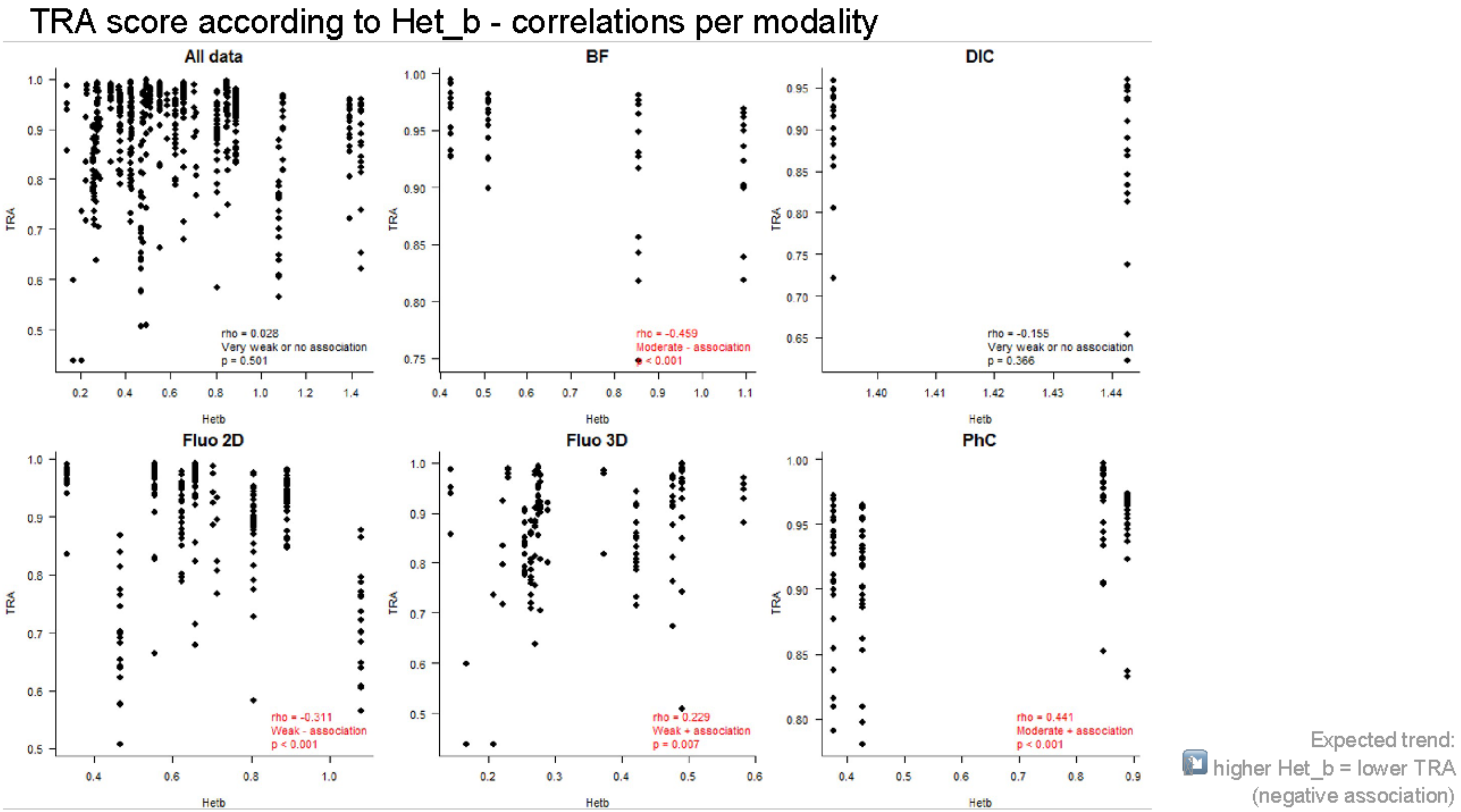

Supplementary Figure 16. Spearman's rank correlation coefficient between Tracking scores and Heterogeneity of the signal between the cells (Het_b). Significance level fixed at 0.05. Measurements given per video sequence.

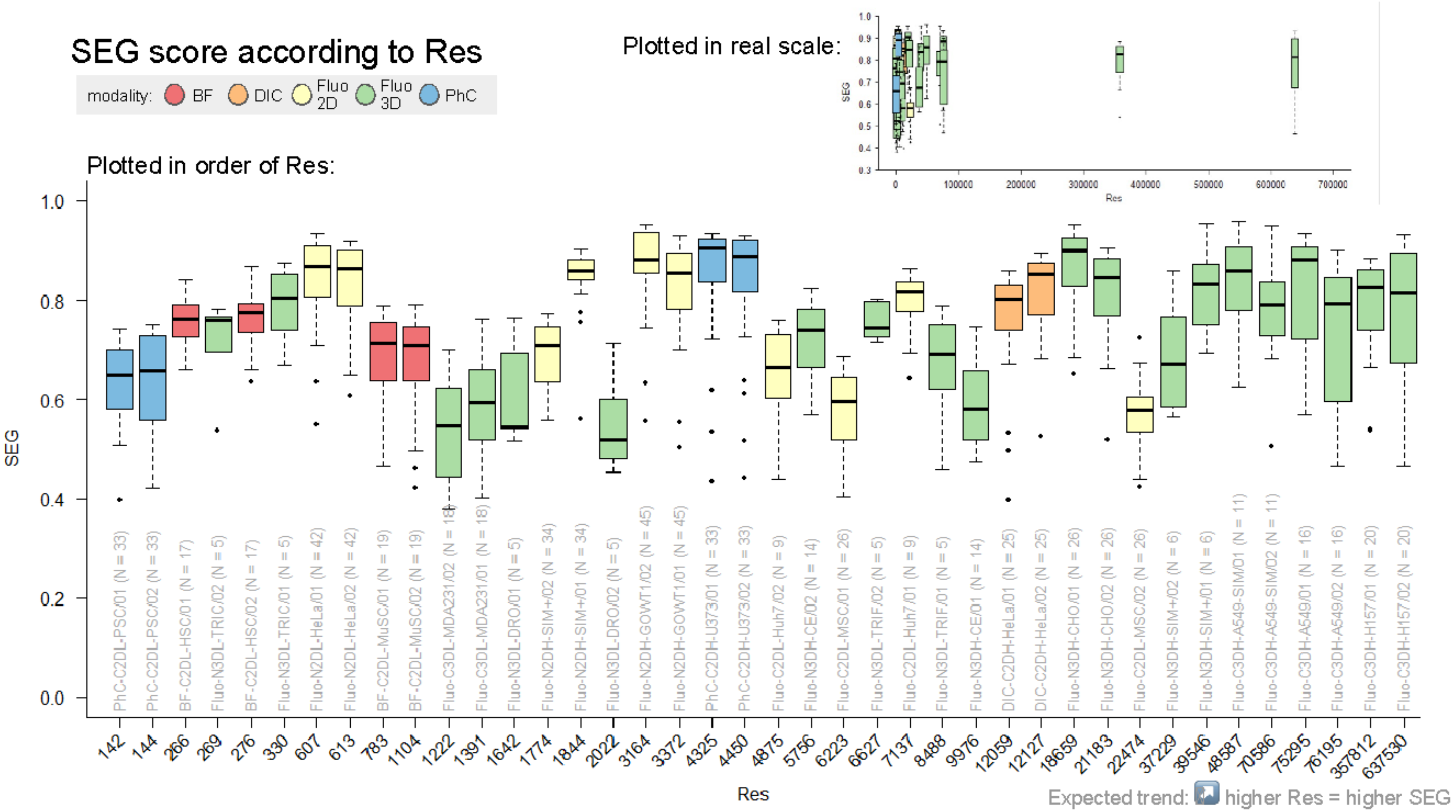

Supplementary Figure 17. Segmentation scores as a function of Resolution (Res) Bold line represents median values. Measurements are given per video sequence. Outliers indicate values higher/lower than 1.5 times the interquartile range.

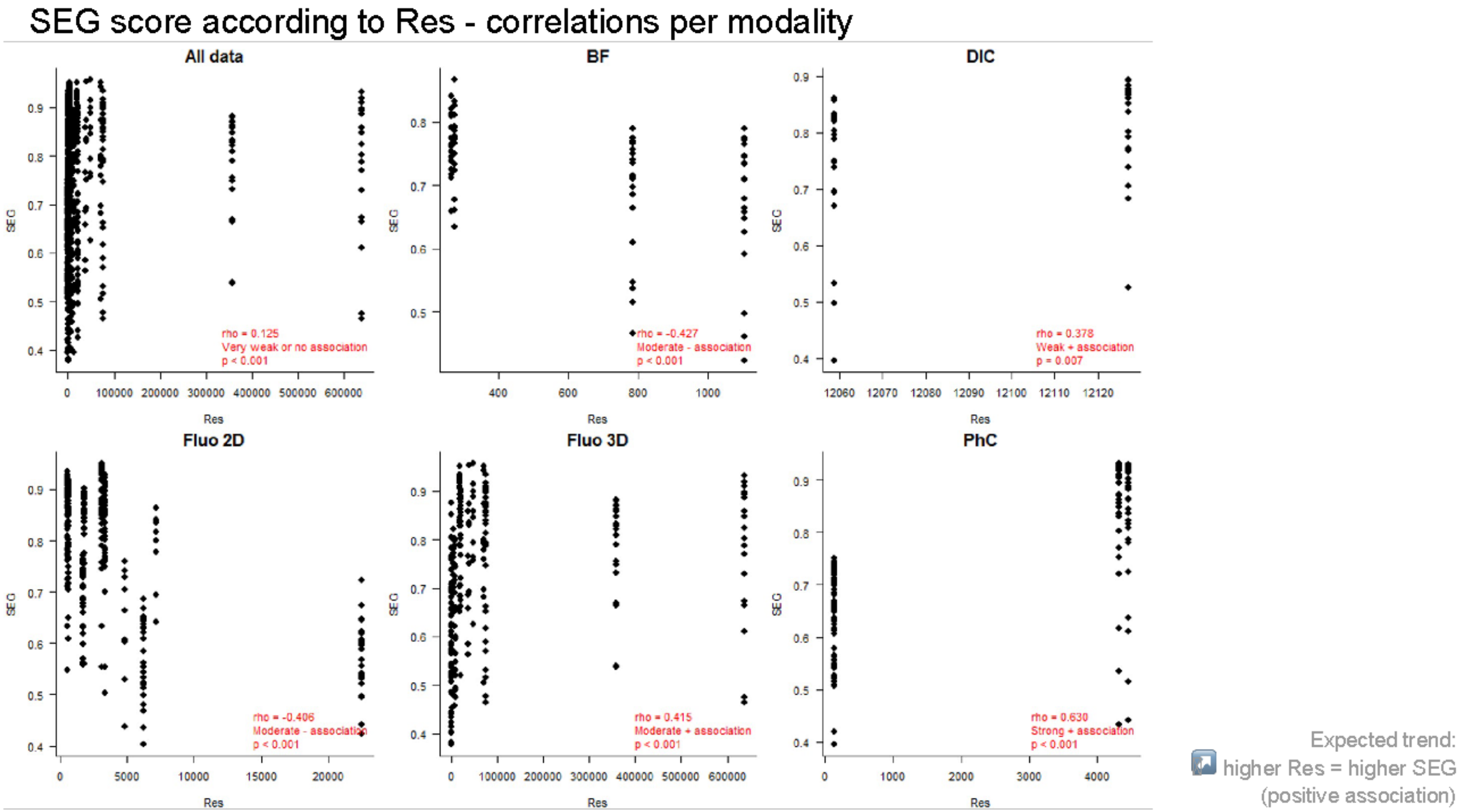

Supplementary Figure 18. Spearman's rank correlation coefficient between Segmentation scores and Resolution (Res). Significance level fixed at 0.05. Measurements given per video sequence.

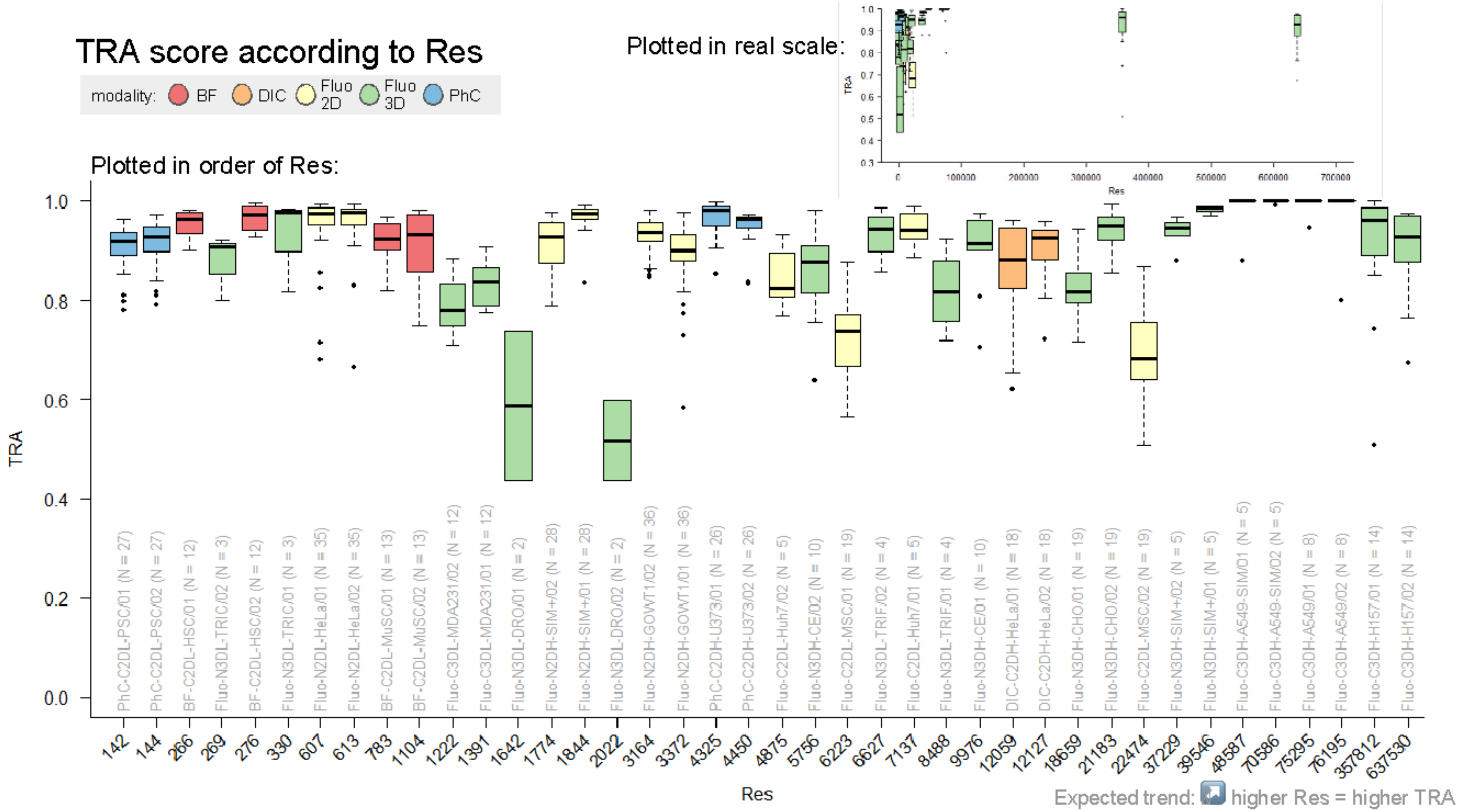

Supplementary Figure 19. Tracking scores as a function of Resolution (Res) Bold line represents median values. Measurements are given per video sequence. Outliers indicate values higher/lower than 1.5 times the interquartile range.

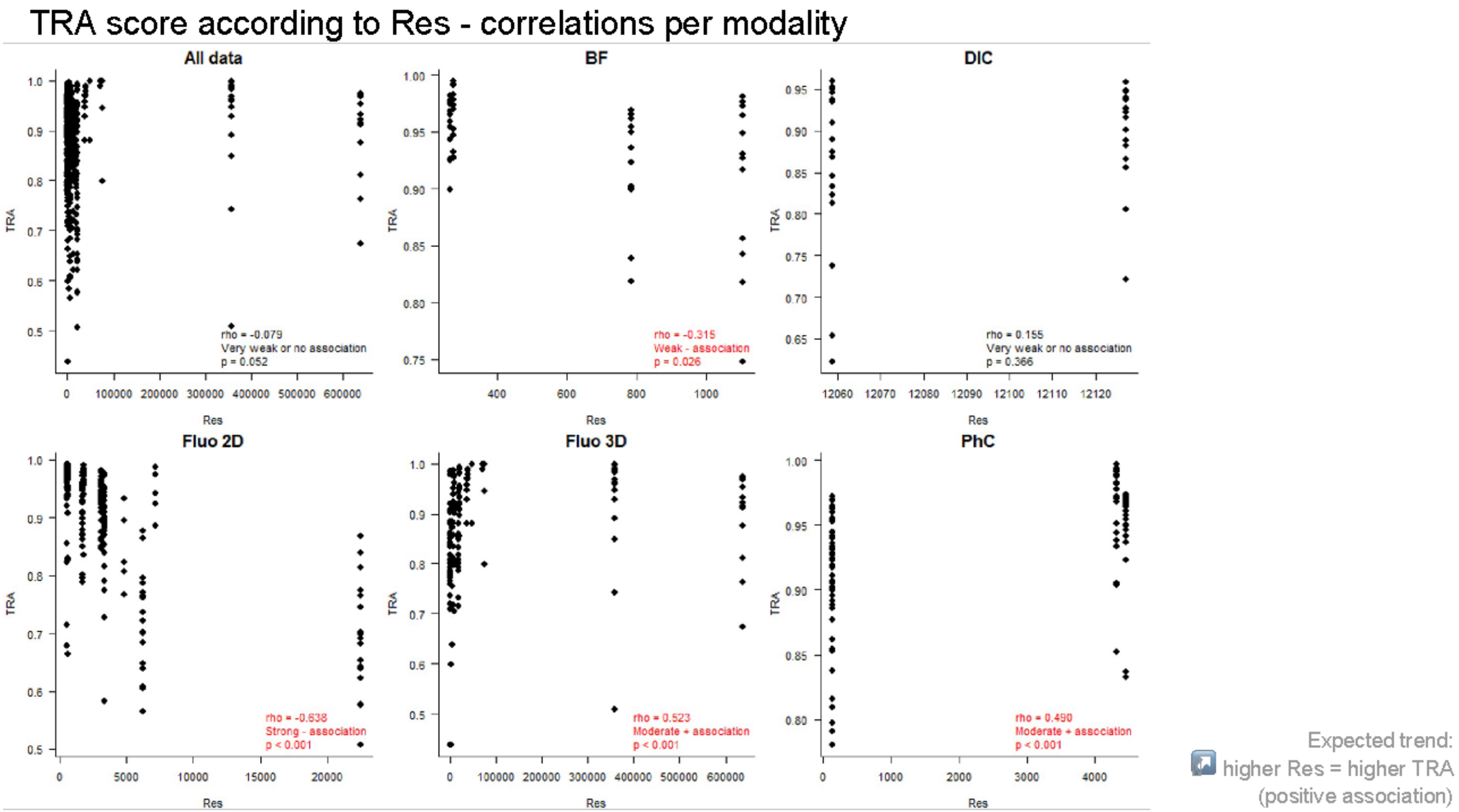

Supplementary Figure 20. Spearman's rank correlation coefficient between Tracking scores and Resolution (Res). Significance level fixed at 0.05. Measurements given per video sequence.

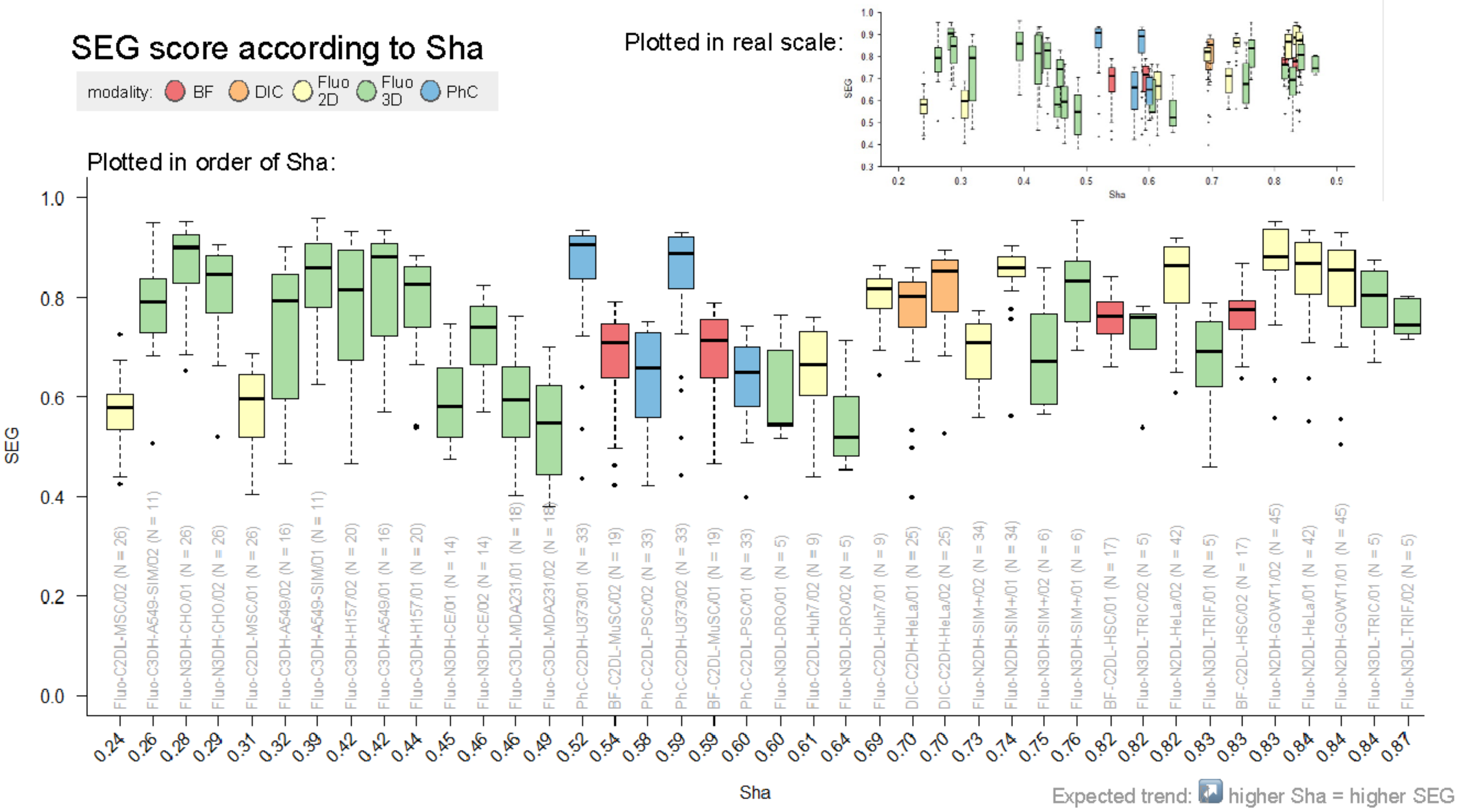

Supplementary Figure 21. Segmentation scores as a function of Shape (Sha) Bold line represents median values. Measurements are given per video sequence. Outliers indicate values higher/lower than 1.5 times the interquartile range

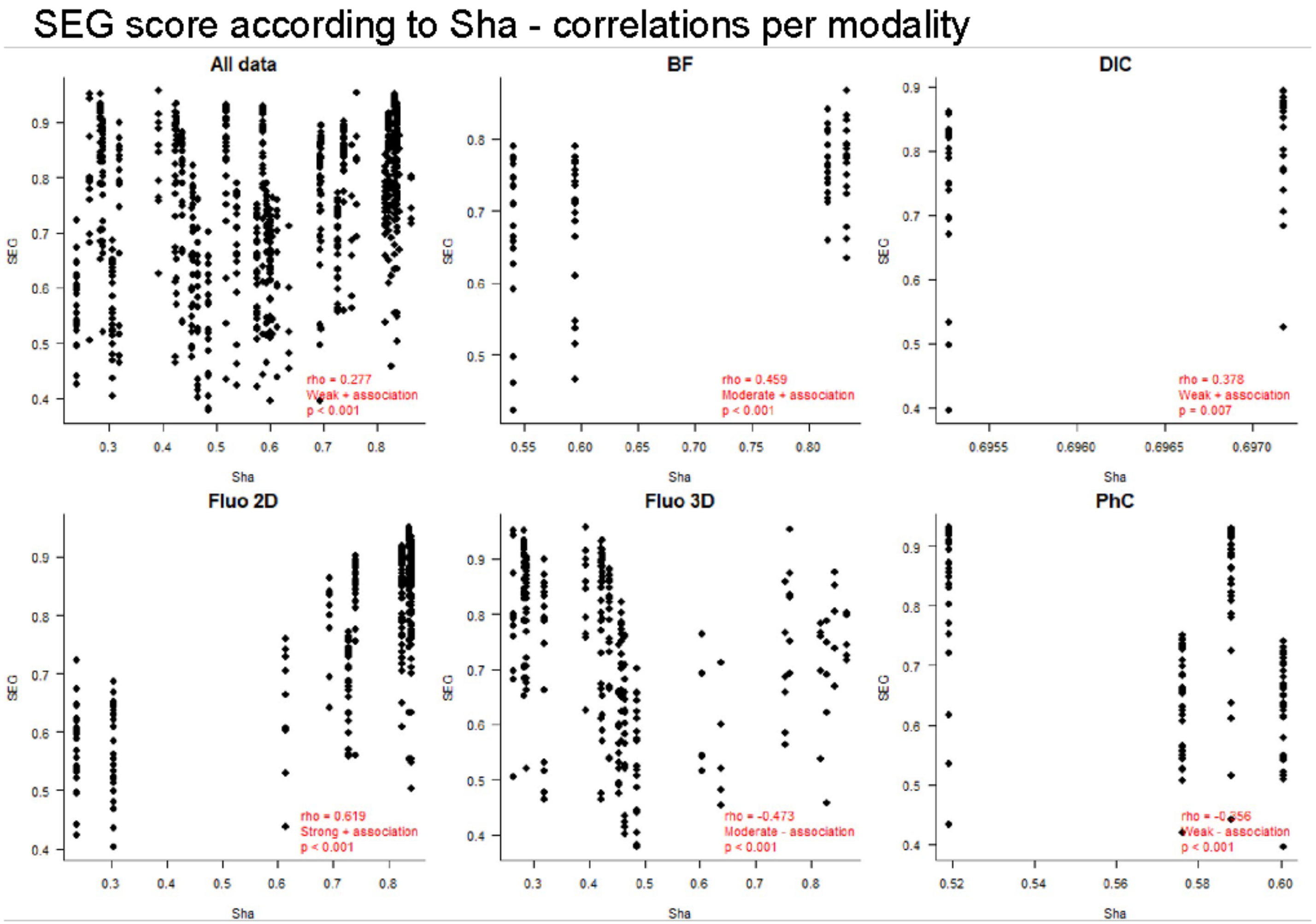

Expected trend:
higher Sha = higher SEG
(positive association)

Supplementary Figure 22. Spearman's rank correlation coefficient between Segmentation scores and Shape (Sha). Significance level fixed at 0.05. Measurements given per video sequence.

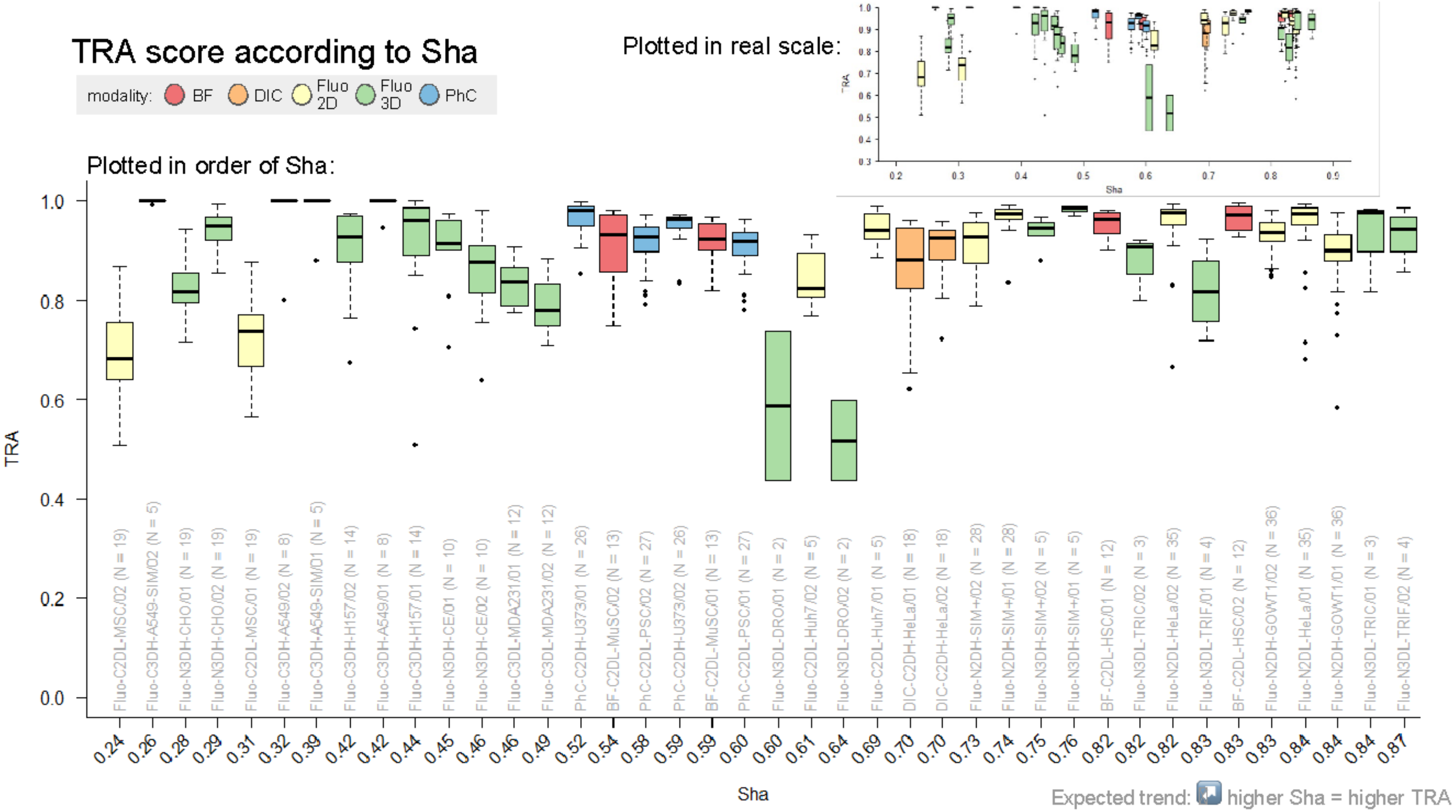

Supplementary Figure 23. Tracking scores as a function of Shape (Sha) Bold line represents median values. Measurements are given per video sequence. Outliers indicate values higher/lower than 1.5 times the interquartile range

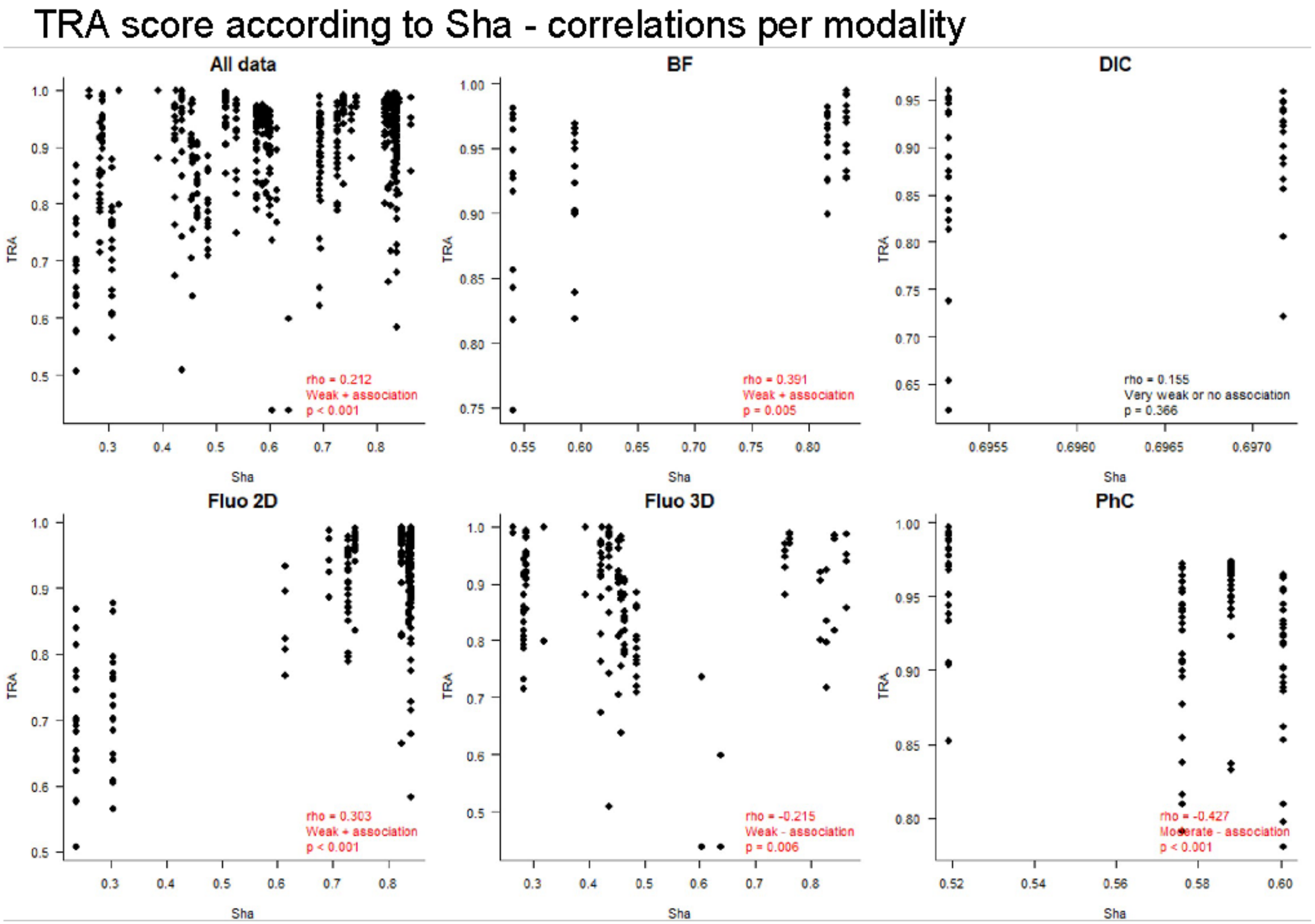

Expected trend:
higher Sha = higher TRA
(positive association)

Supplementary Figure 24. Spearman's rank correlation coefficient between Tracking scores and Shape (Sha). Significance level fixed at 0.05. Measurements given per video sequence.

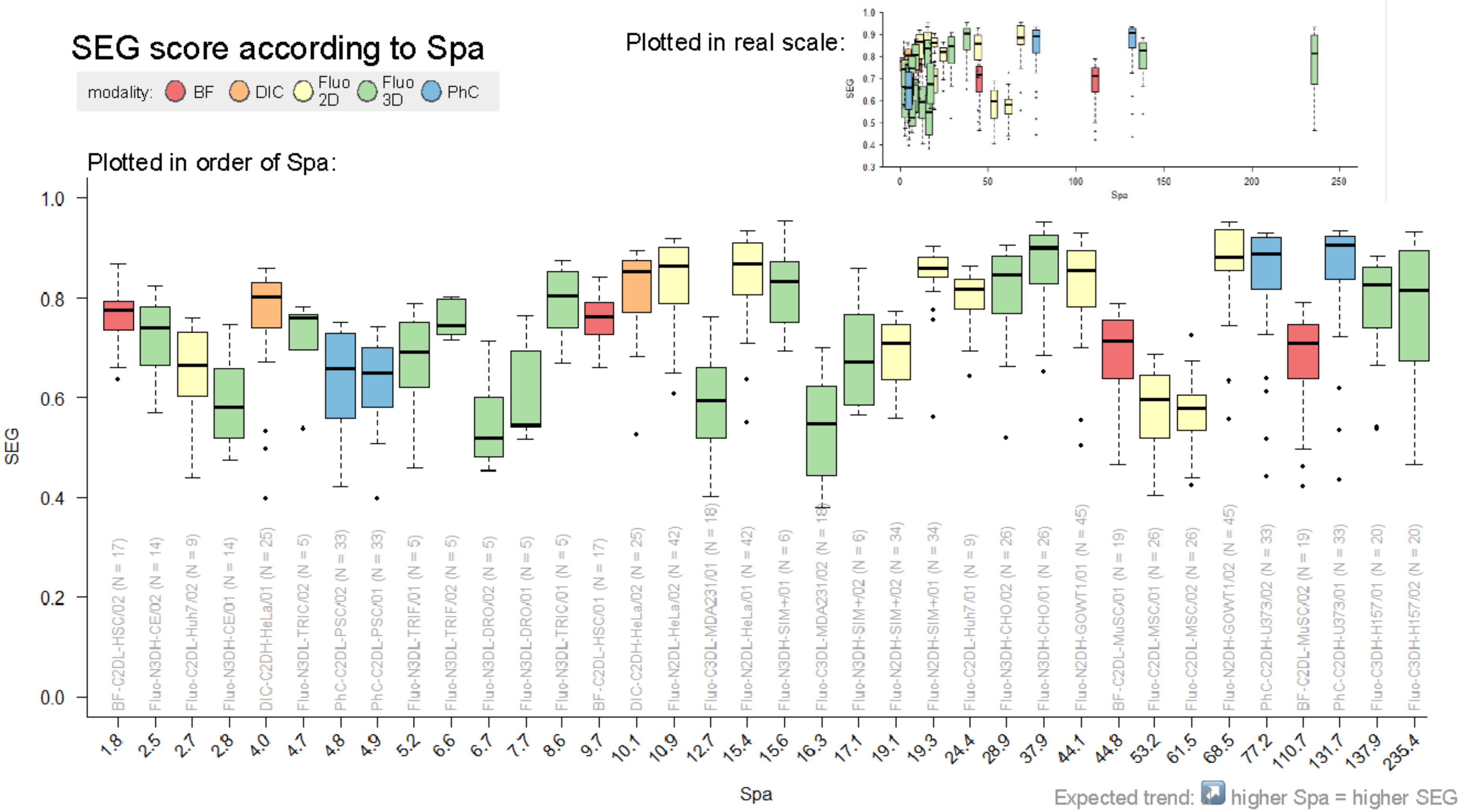

Supplementary Figure 25. Segmentation scores as a function of Spacing between cells (Spa) Bold line represents median values. Measurements are given per video sequence. Outliers indicate values higher/lower than 1.5 times the interquartile range.

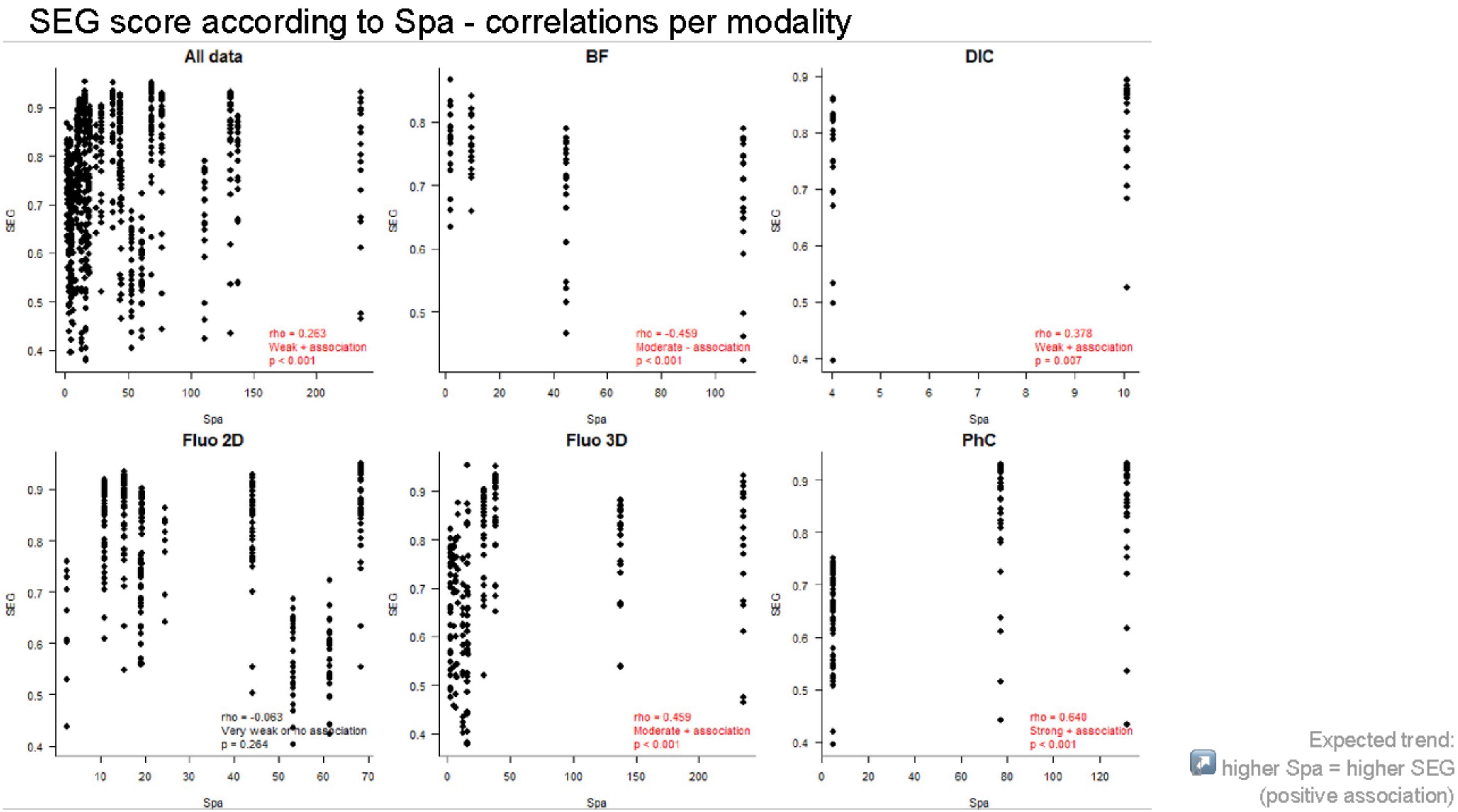

Supplementary Figure 26. Spearman's rank correlation coefficient between Segmentation scores and Spacing between cells (Spa). Significance level fixed at 0.05. Measurements given per video sequence

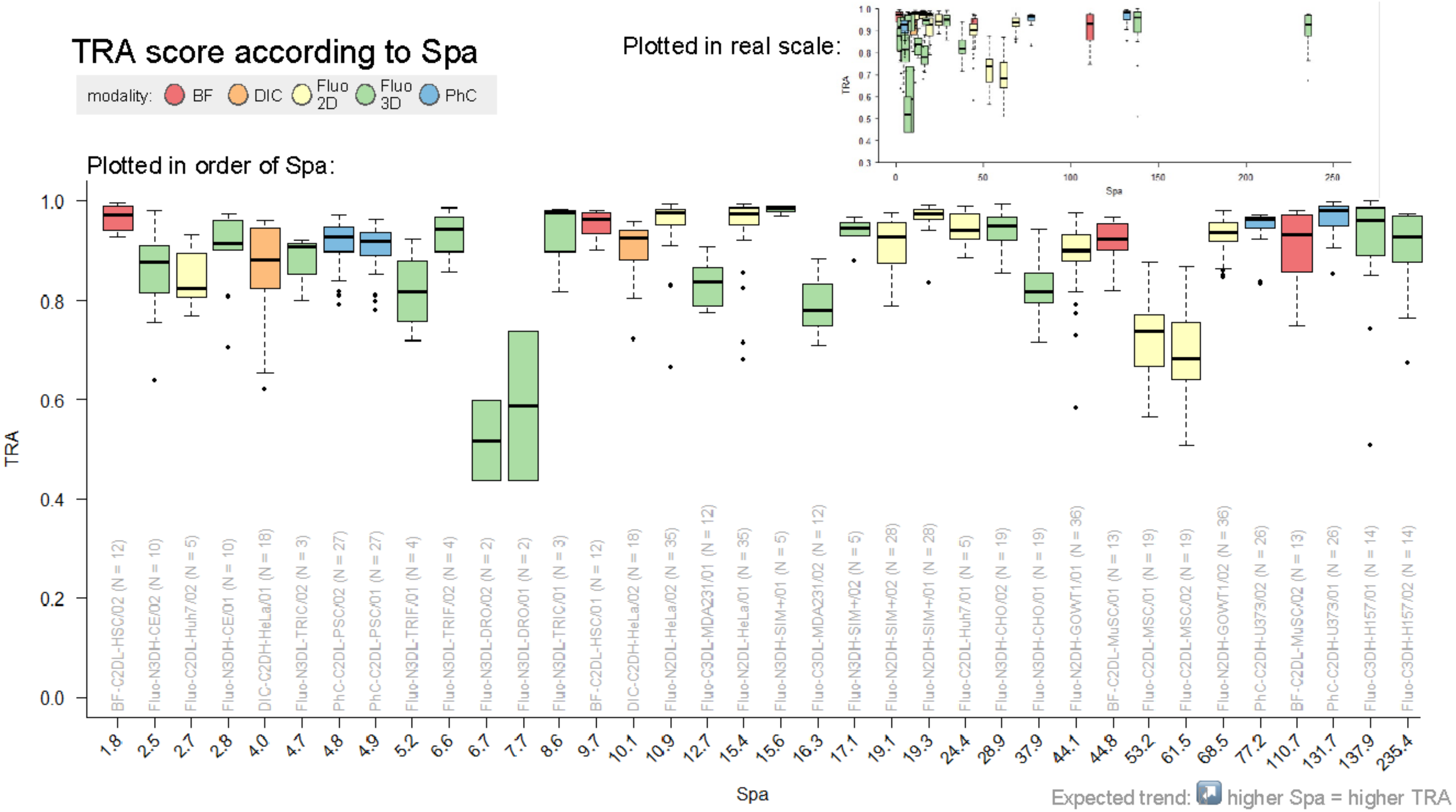

Supplementary Figure 27. Tracking scores as a function of Spacing between cells (Spa) Bold line represents median values. Measurements are given per video sequence. Outliers indicate values higher/lower than 1.5 times the interquartile range

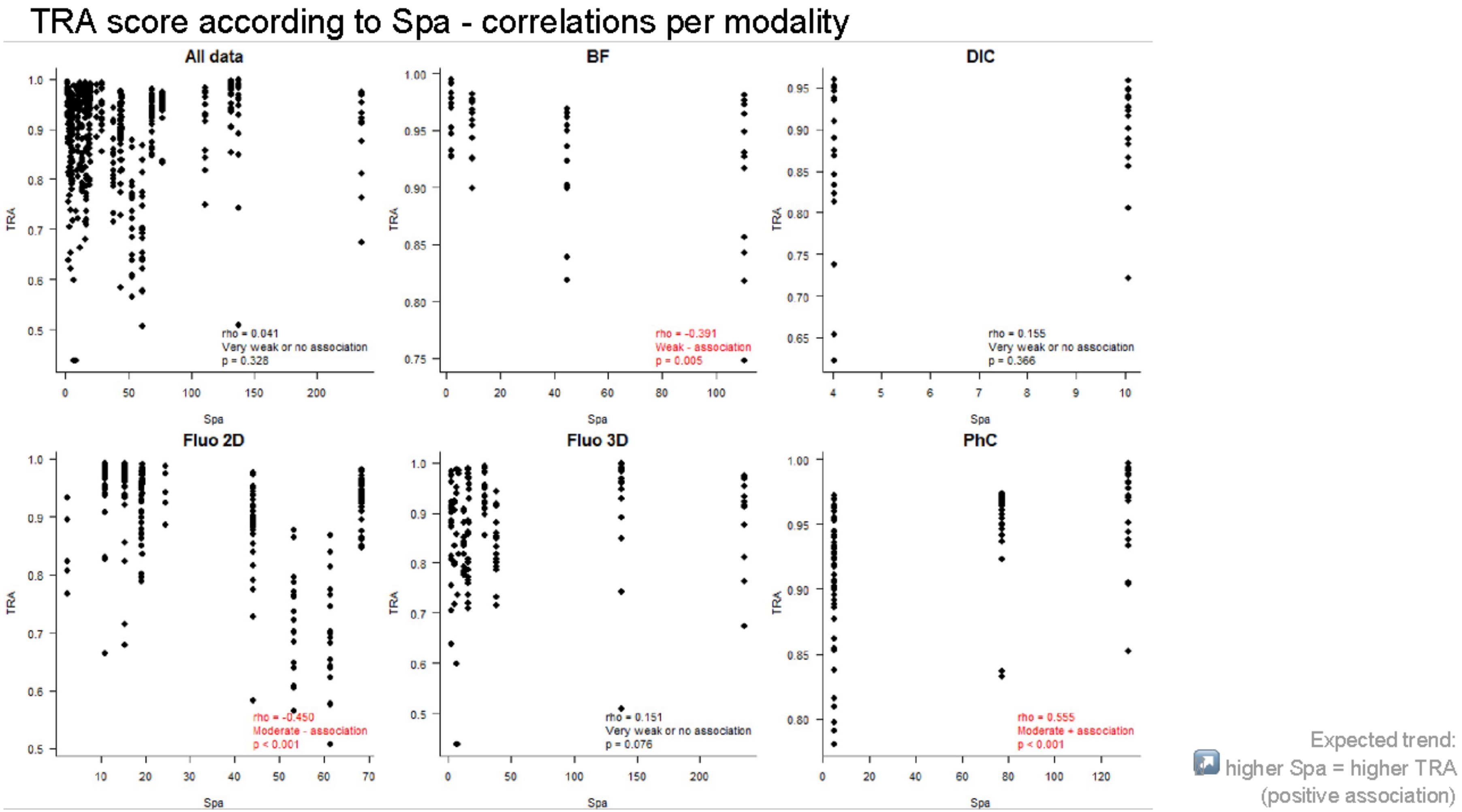

Supplementary Figure 28. Spearman's rank correlation coefficient between Tracking scores and Spacing between cells (Spa). Significance level fixed at 0.05. Measurements given per video sequence

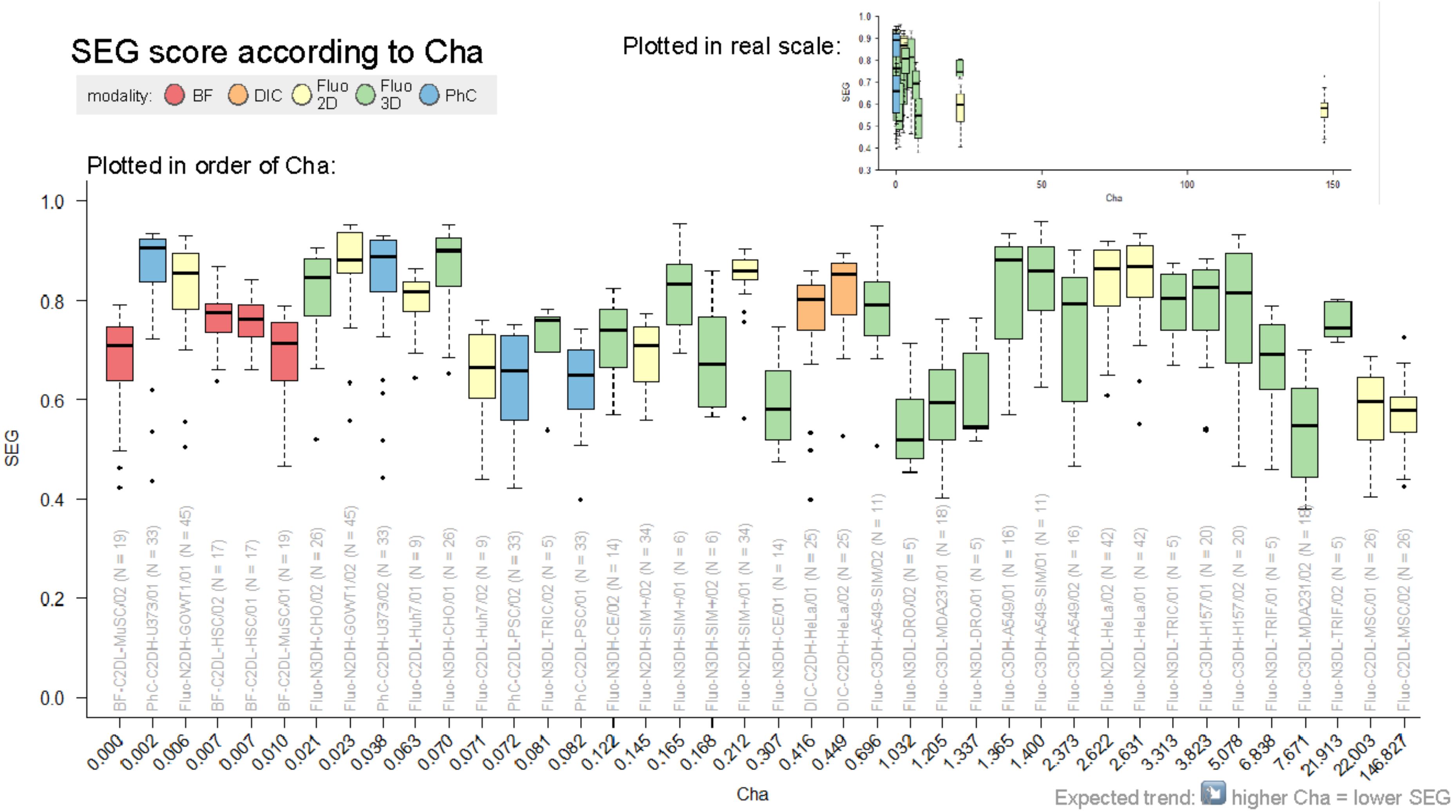

Supplementary Figure 29. Segmentation scores as a function of the Change of signal intensity with time (Cha) Bold line represents median values. Measurements are given per video sequence. Outliers indicate values higher/lower than 1.5 times the interquartile range.

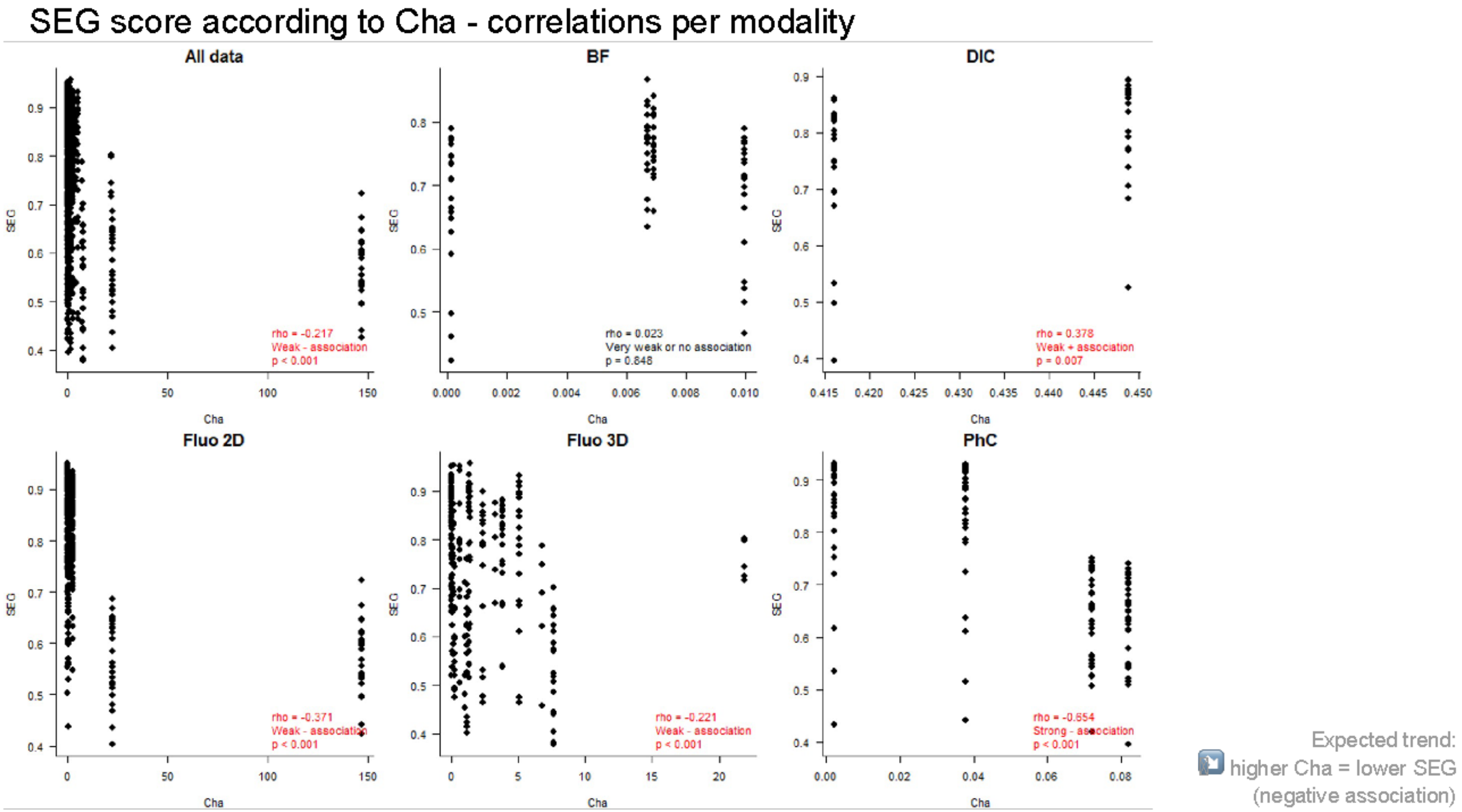

Supplementary Figure 30. Spearman's rank correlation coefficient between Segmentation scores and Change of signal intensity with time (Cha). Significance level fixed at 0.05. Measurements given per video sequence.

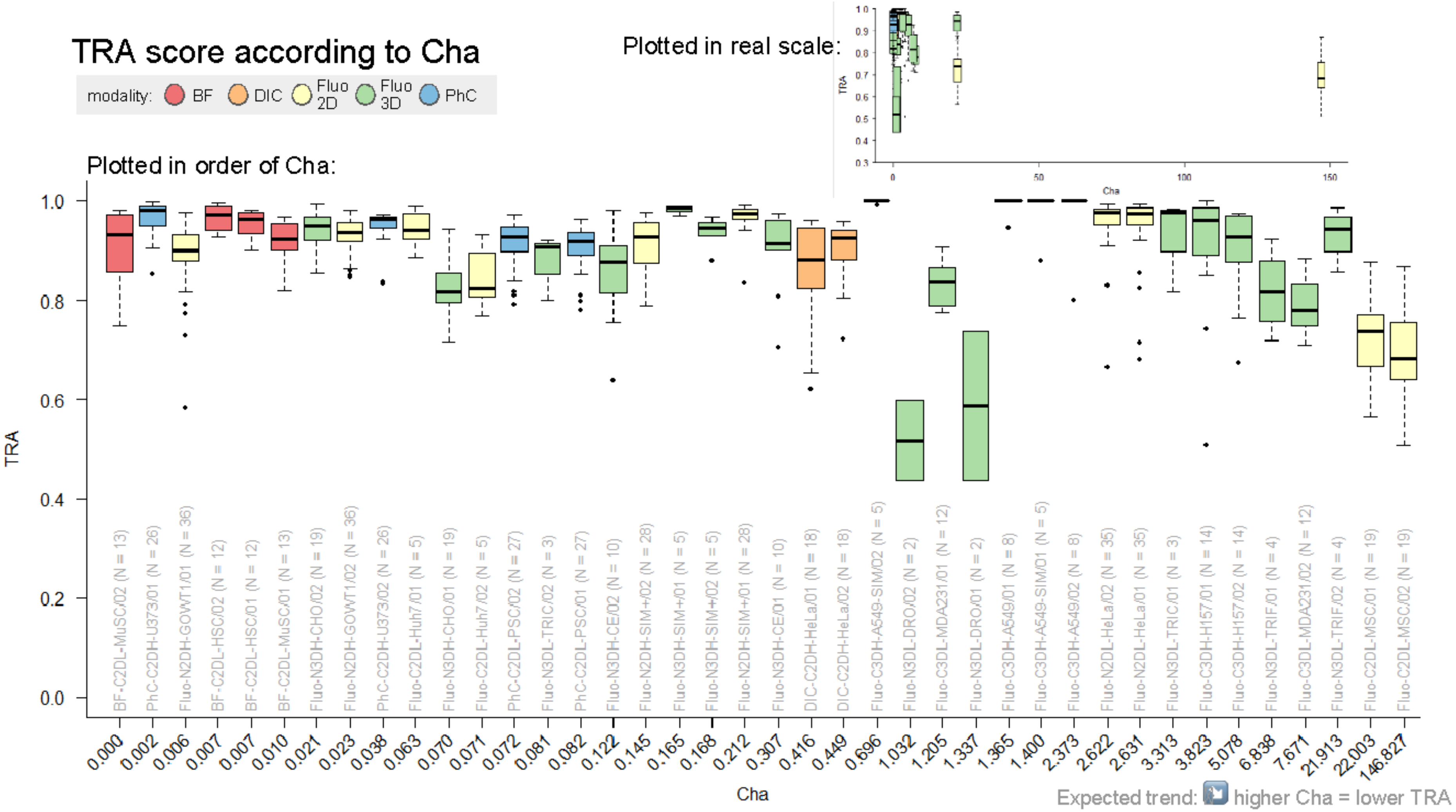

Supplementary Figure 31. Tracking scores as a function of the Change of signal intensity with time (Cha) Bold line represents median values. Measurements are given per video sequence. Outliers indicate values higher/lower than 1.5 times the interquartile range.

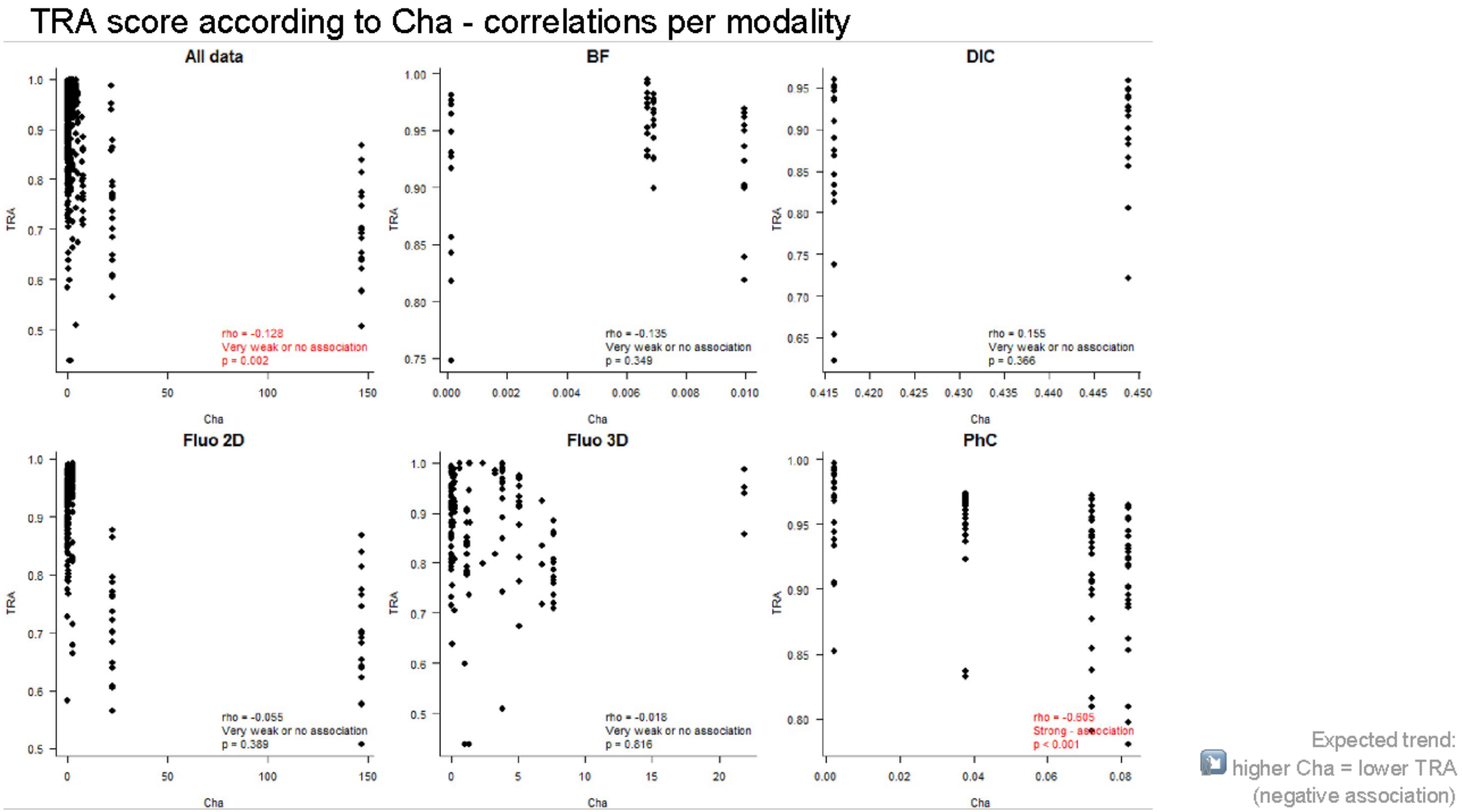

Supplementary Figure 32. Spearman's rank correlation coefficient between Tracking scores and Change of signal intensity with time (Cha). Significance level fixed at 0.05. Measurements given per video sequence

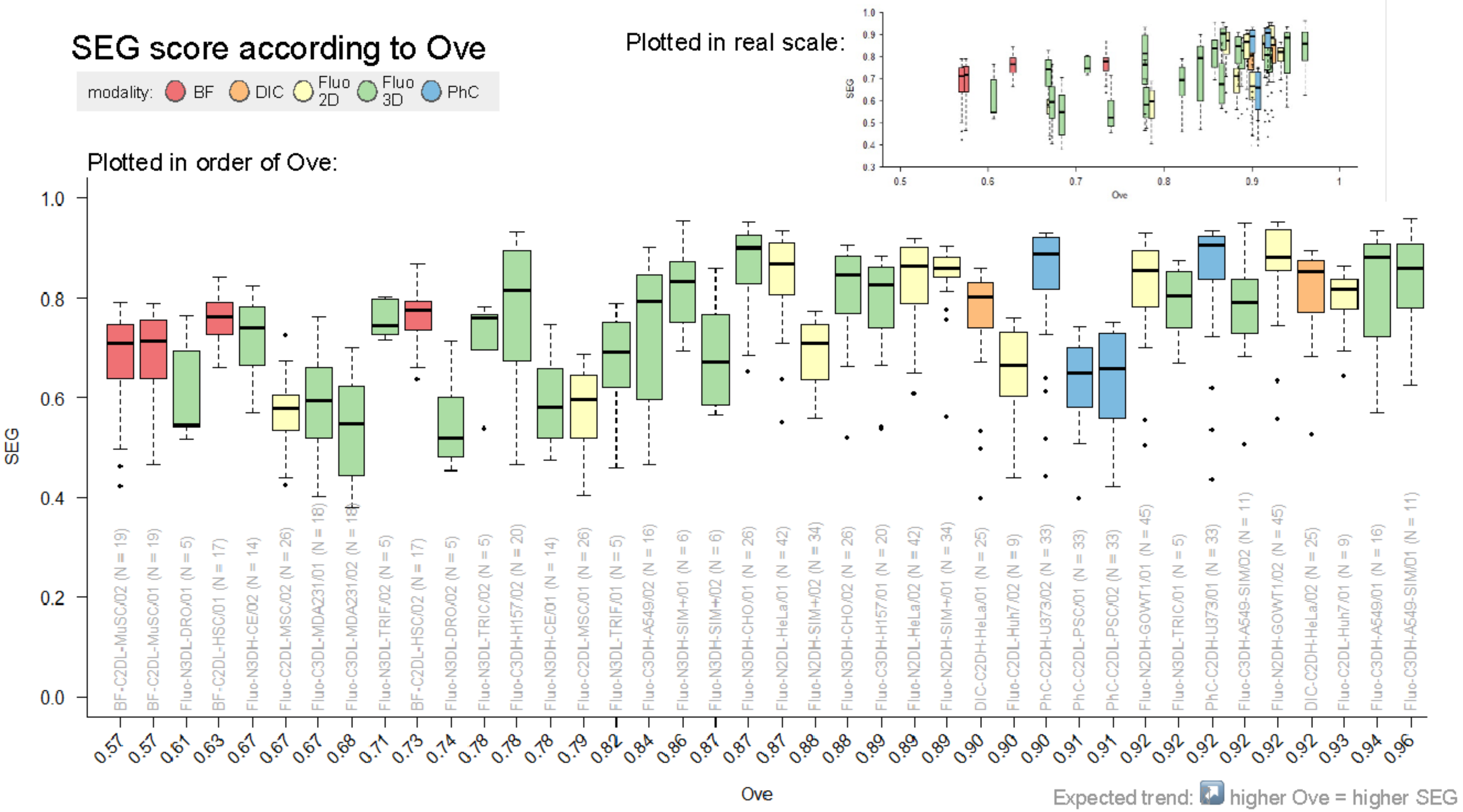

Supplementary Figure 33. Segmentation scores as a function of Overlap (Ove) Bold line represents median values. Measurements are given per video sequence. Outliers indicate values higher/lower than 1.5 times the interquartile range

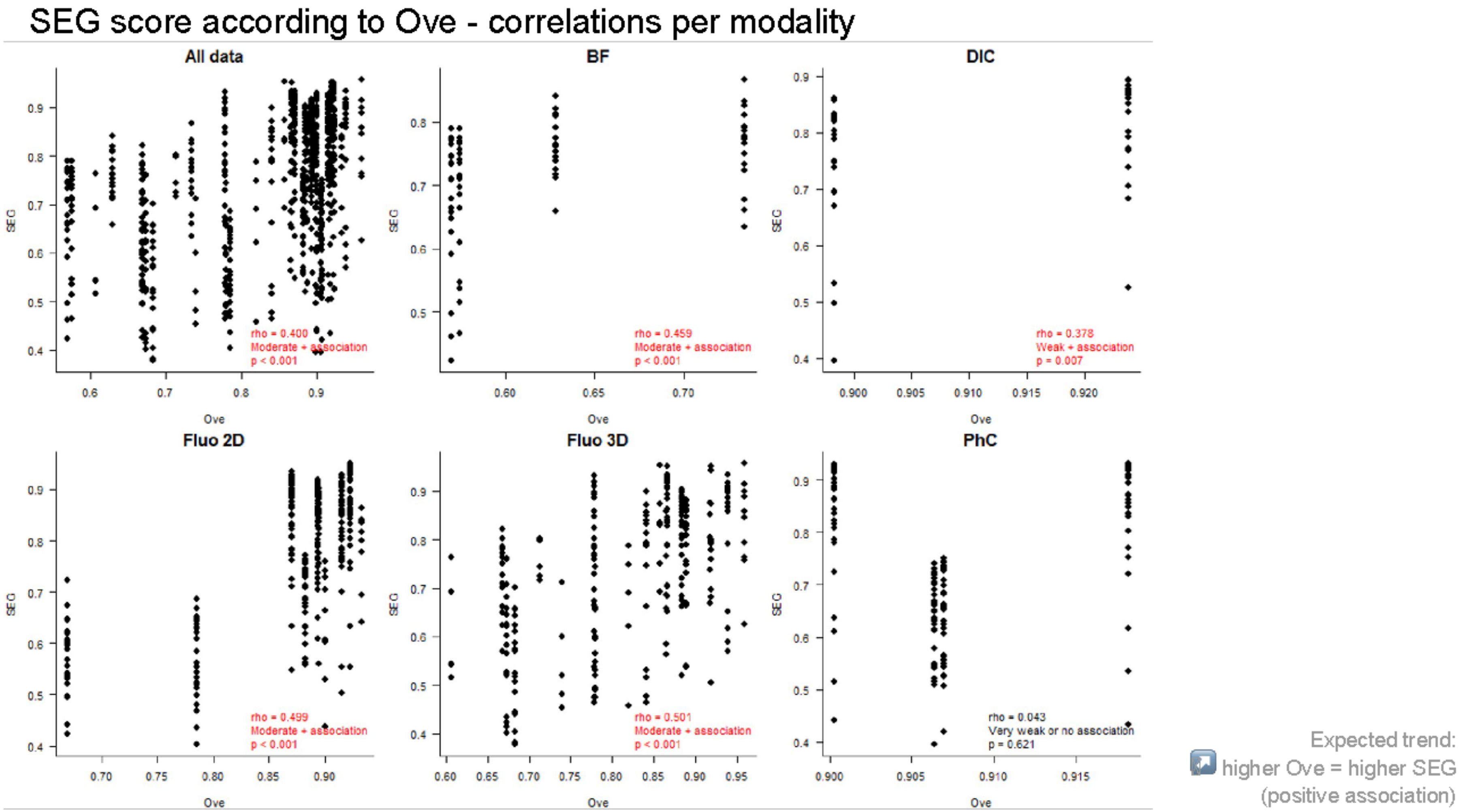

Supplementary Figure 34. Spearman's rank correlation coefficient between Segmentation scores and Overlap (Ove). Significance level fixed at 0.05. Measurements given per video sequence.

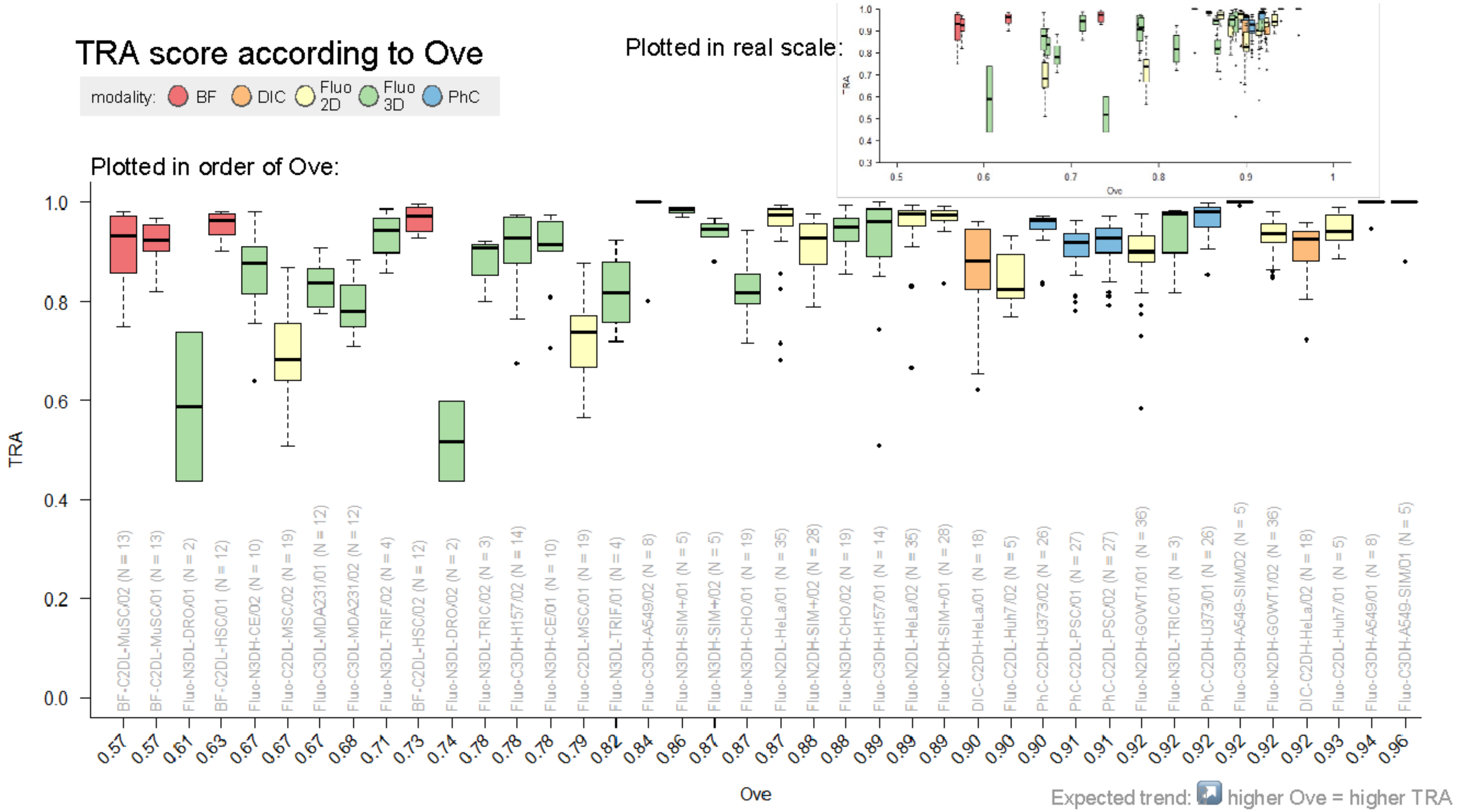

Supplementary Figure 35. Tracking scores as a function of Overlap (Ove) Bold line represents median values. Measurements are given per video sequence. Outliers indicate values higher/lower than 1.5 times the interquartile range.

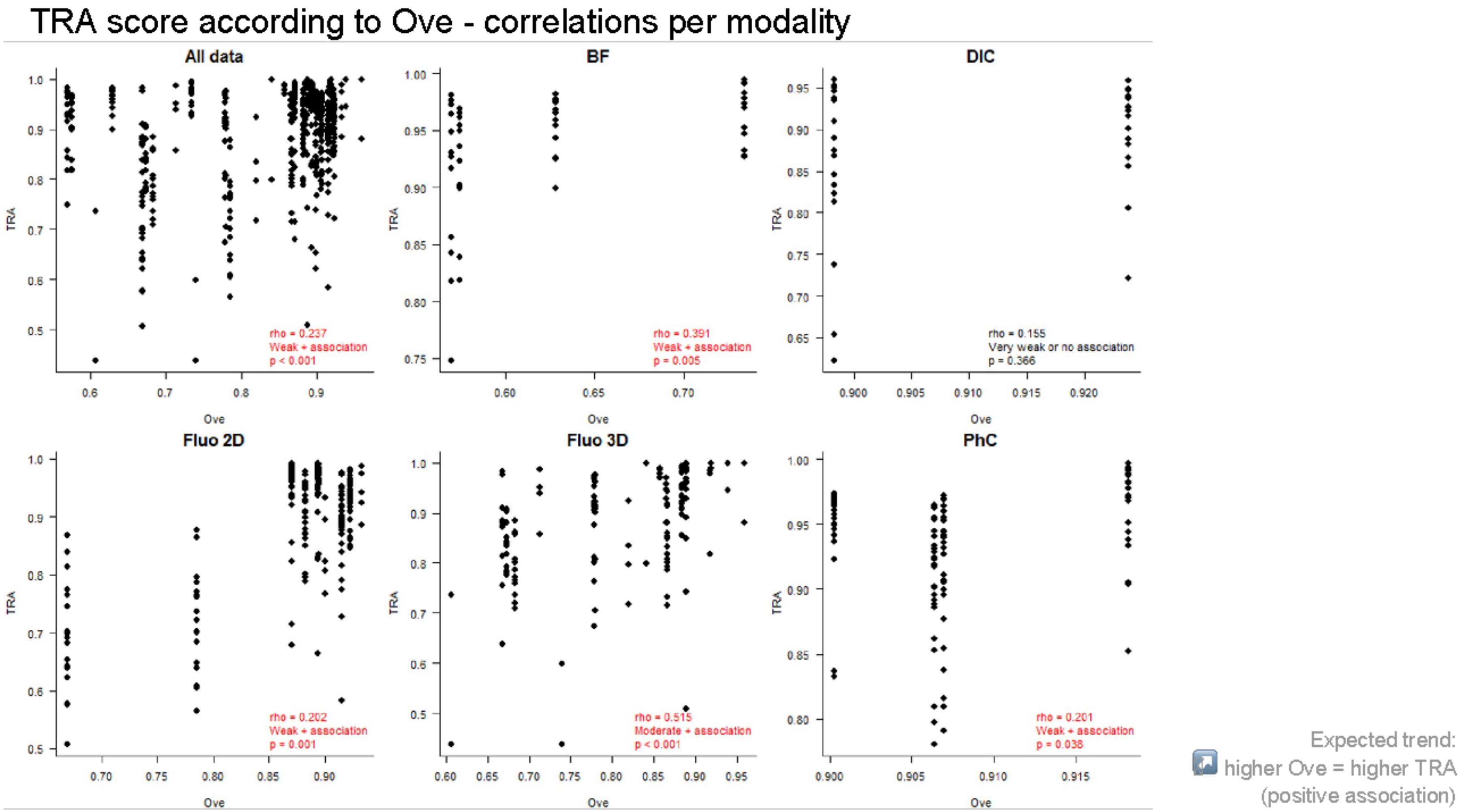

Supplementary Figure 36. Spearman's rank correlation coefficient between Tracking scores and Overlap (Ove). Significance level fixed at 0.05. Measurements given per video sequence.

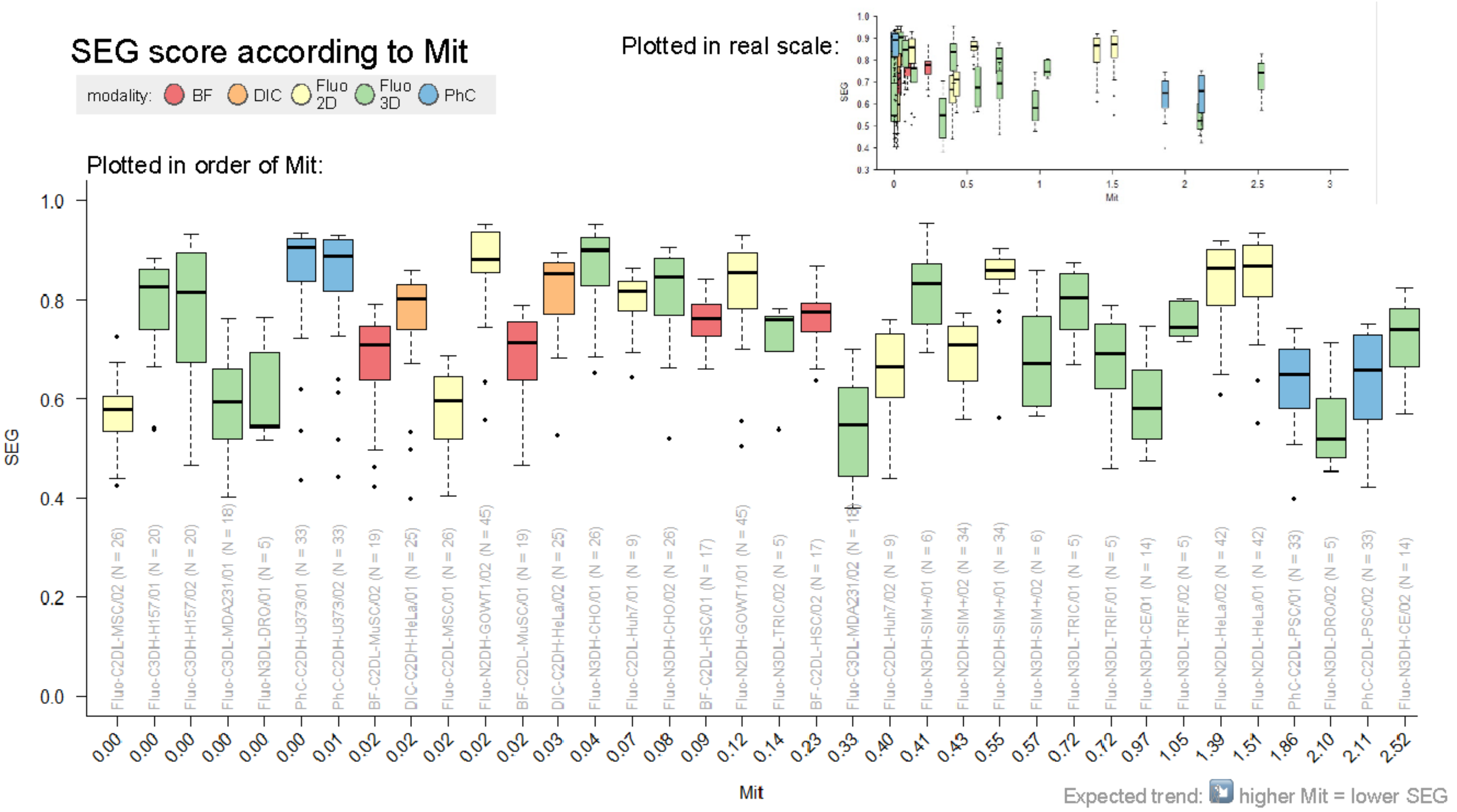

Supplementary Figure 37. Segmentation scores as a function of Mitotic division rate (Mit) Bold line represents median values. Measurements are given per video sequence. Outliers indicate values higher/lower than 1.5 times the interquartile range.

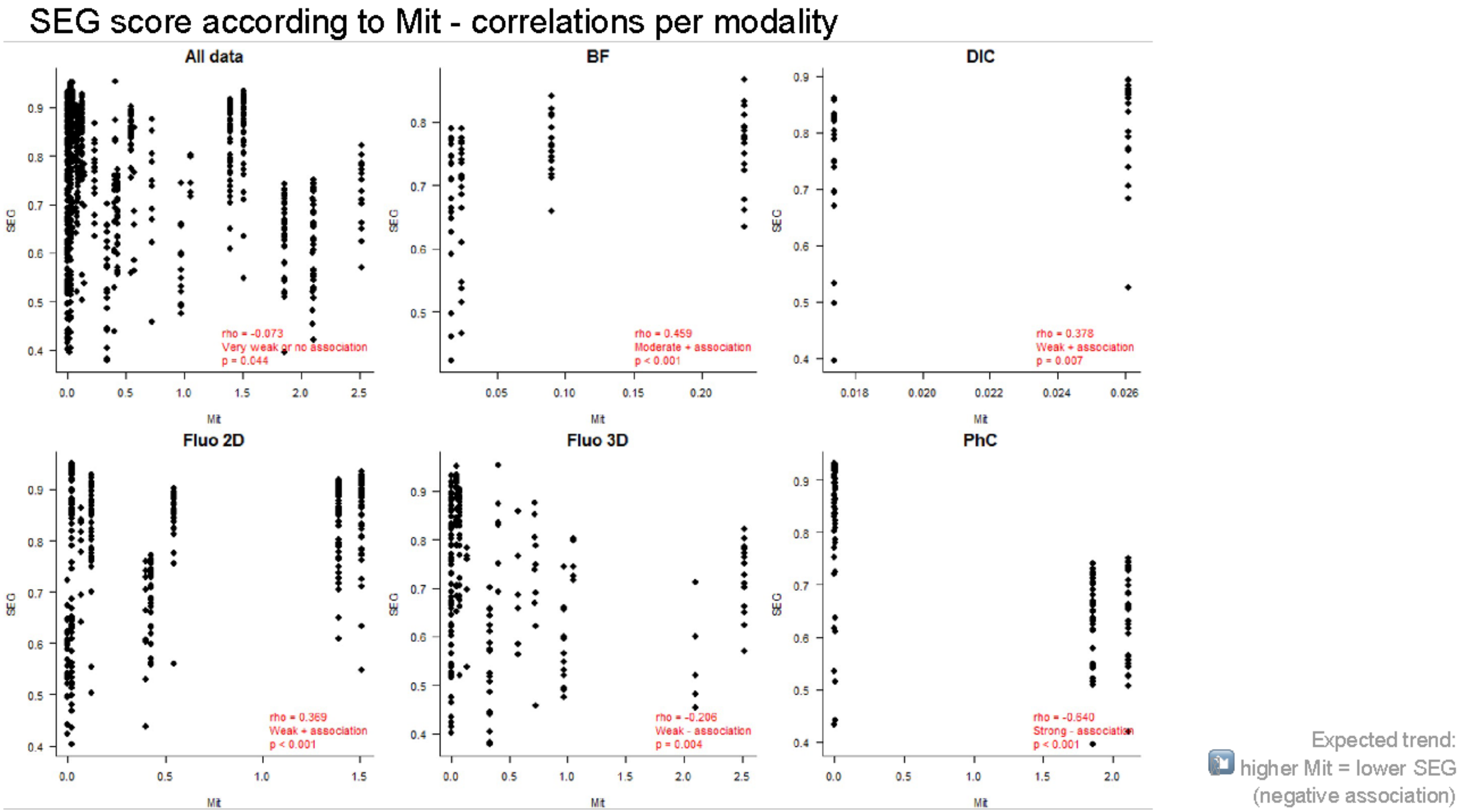

Supplementary Figure 38. Spearman's rank correlation coefficient between Segmentation scores and Mitotic division rate (Mit). Significance level fixed at 0.05. Measurements given per video sequence.

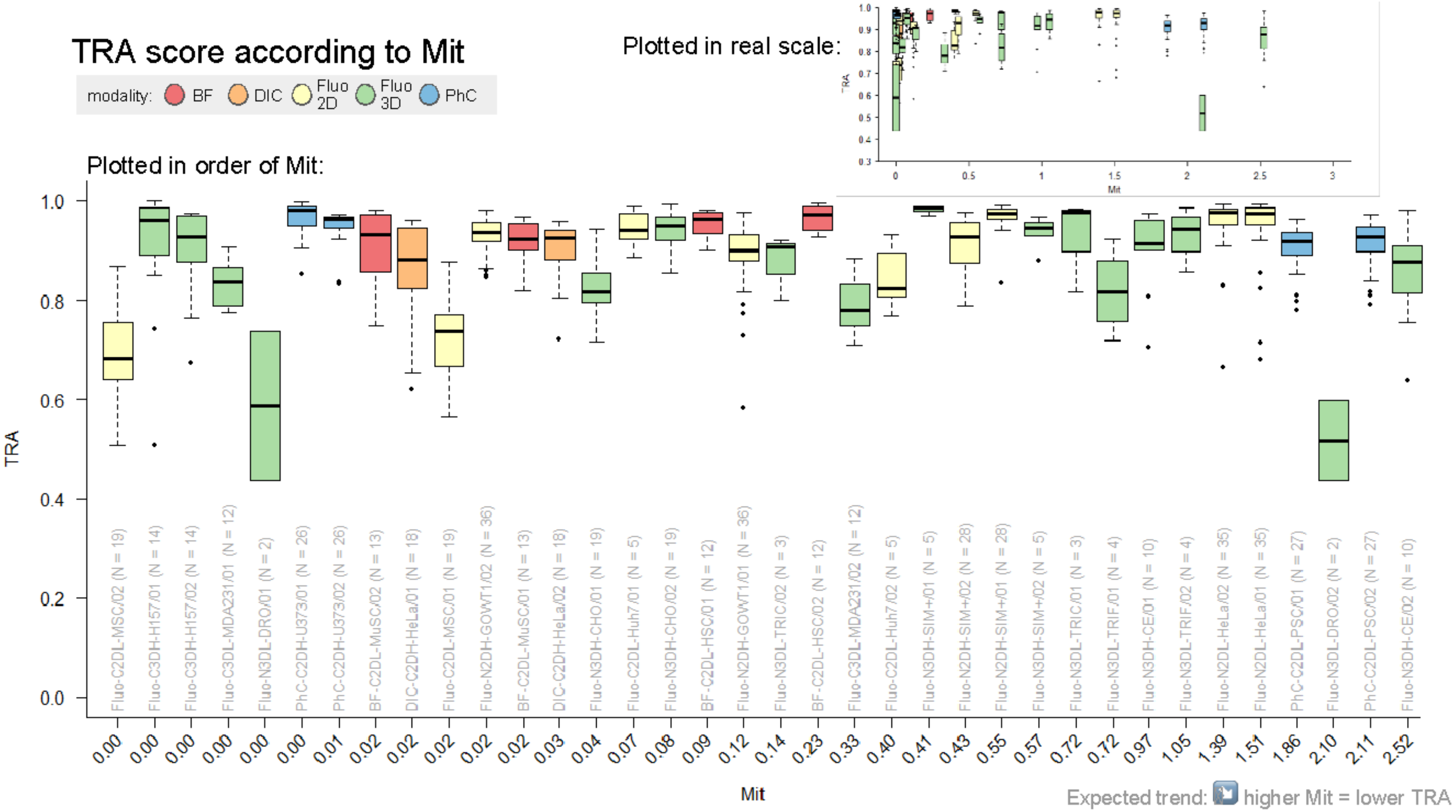

Supplementary Figure 39. Tracking scores as a function of Mitotic division rate (Mit) Bold line represents median values. Measurements are given per video sequence. Outliers indicate values higher/lower than 1.5 times the interquartile range.

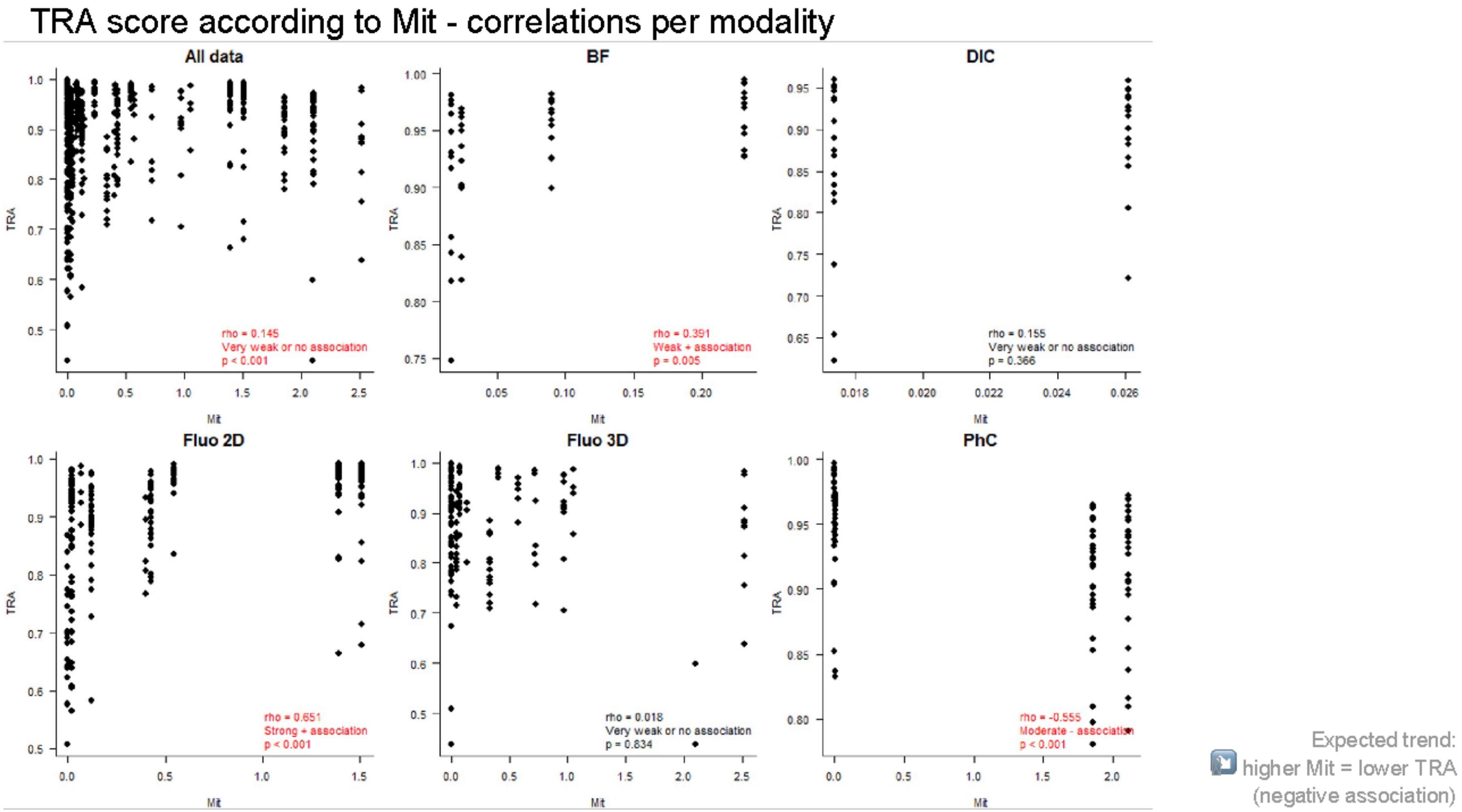

Supplementary Figure 40. Spearman's rank correlation coefficient between Tracking scores and Mitotic division rate (Mit). Significance level fixed at 0.05. Measurements given per video sequence

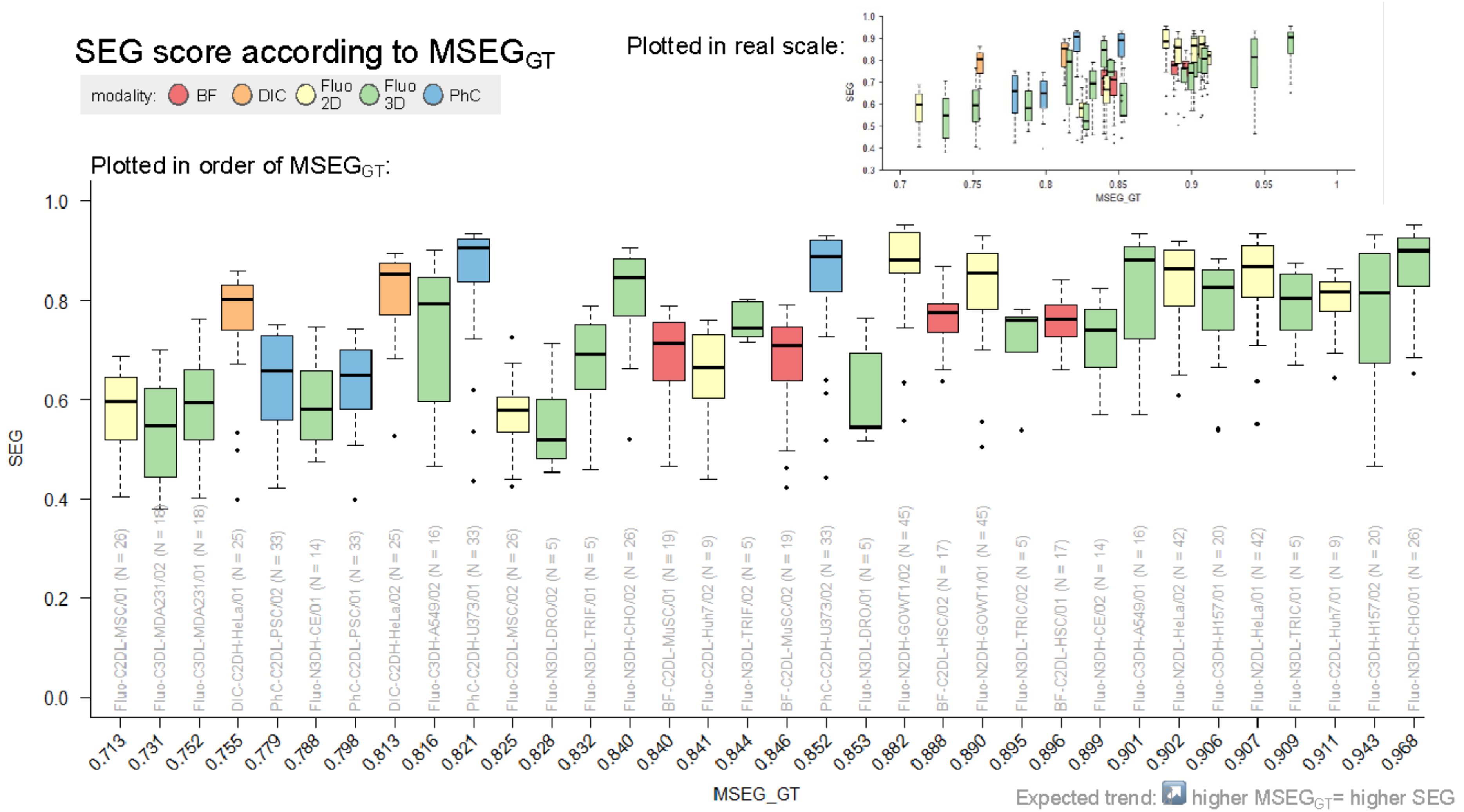

Supplementary Figure 41. Segmentation scores as a function of the quality of the GT Segmentation annotation ($MSEG_{GT}$) Bold line represents median values. Measurements are given per video sequence. Outliers indicate values higher/lower than 1.5 times the interquartile range.

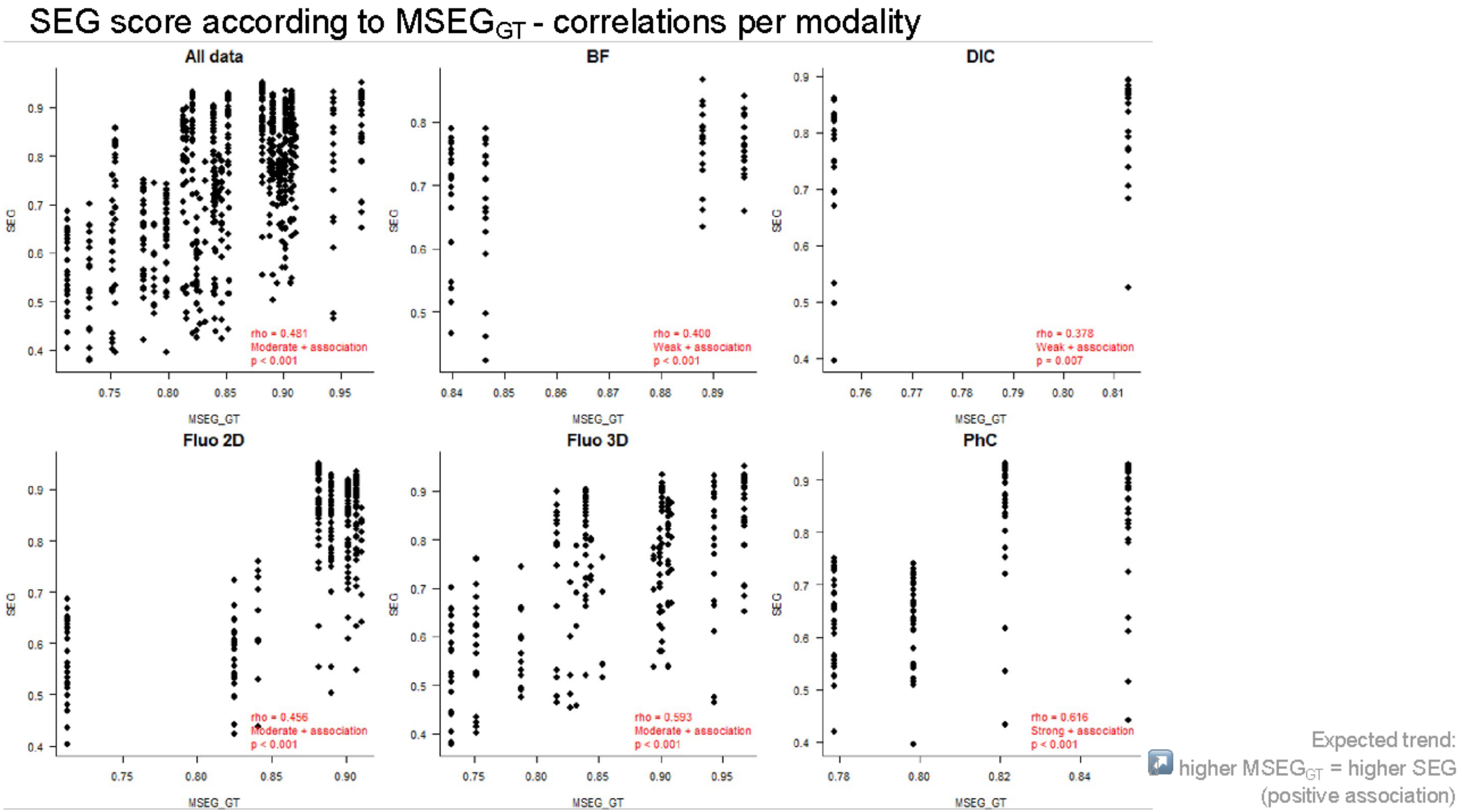

Supplementary Figure 42. Spearman's rank correlation coefficient between Segmentation scores and the quality of the GT Segmentation annotation (MSEG$_{GT}$). Significance level fixed at 0.05. Measurements given per video sequence.

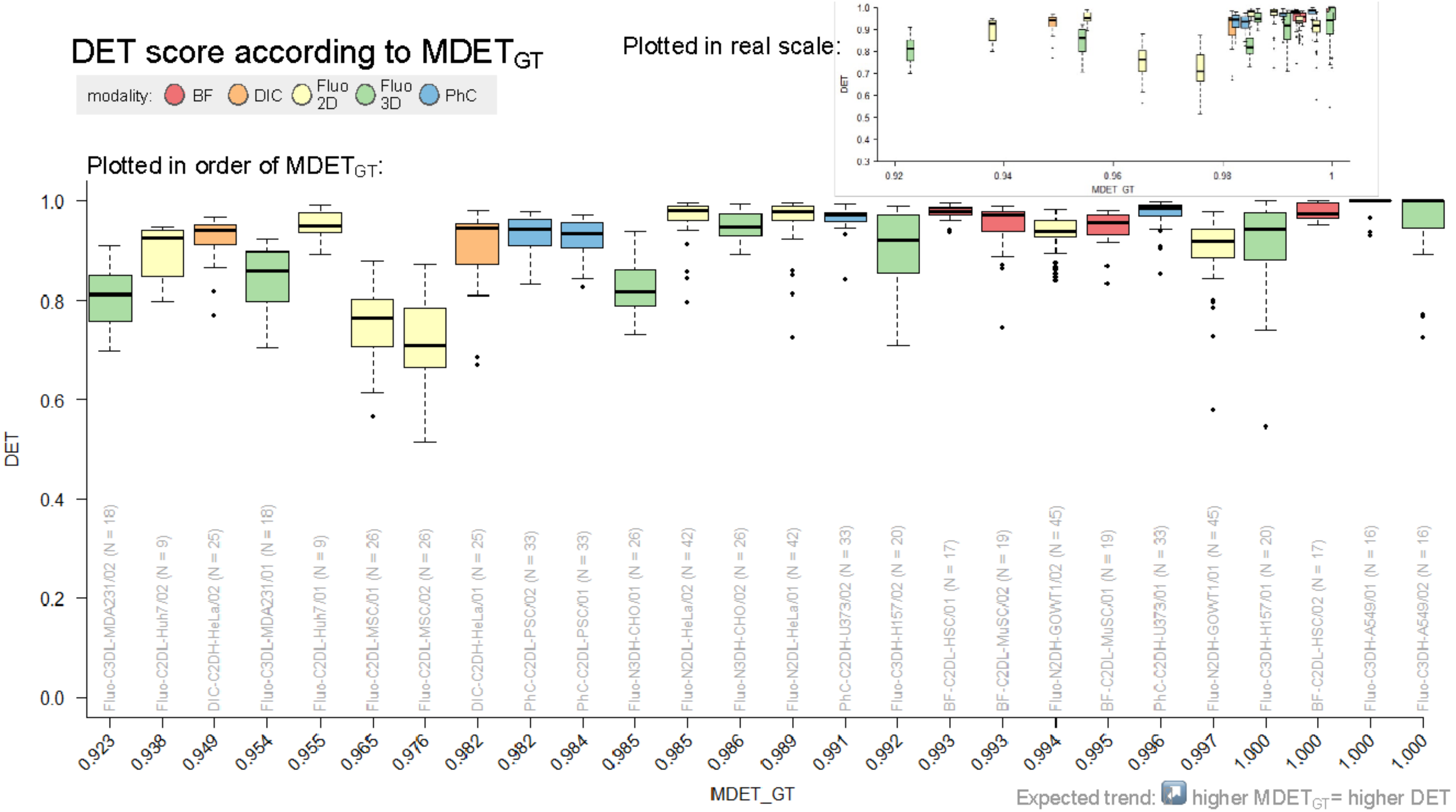

Supplementary Figure 43. Detection scores as a function of the quality of the GT Detection annotation ($MDET_{GT}$). Bold line represents median values. Measurements are given per video sequence. Outliers indicate values higher/lower than 1.5 times the interquartile range.

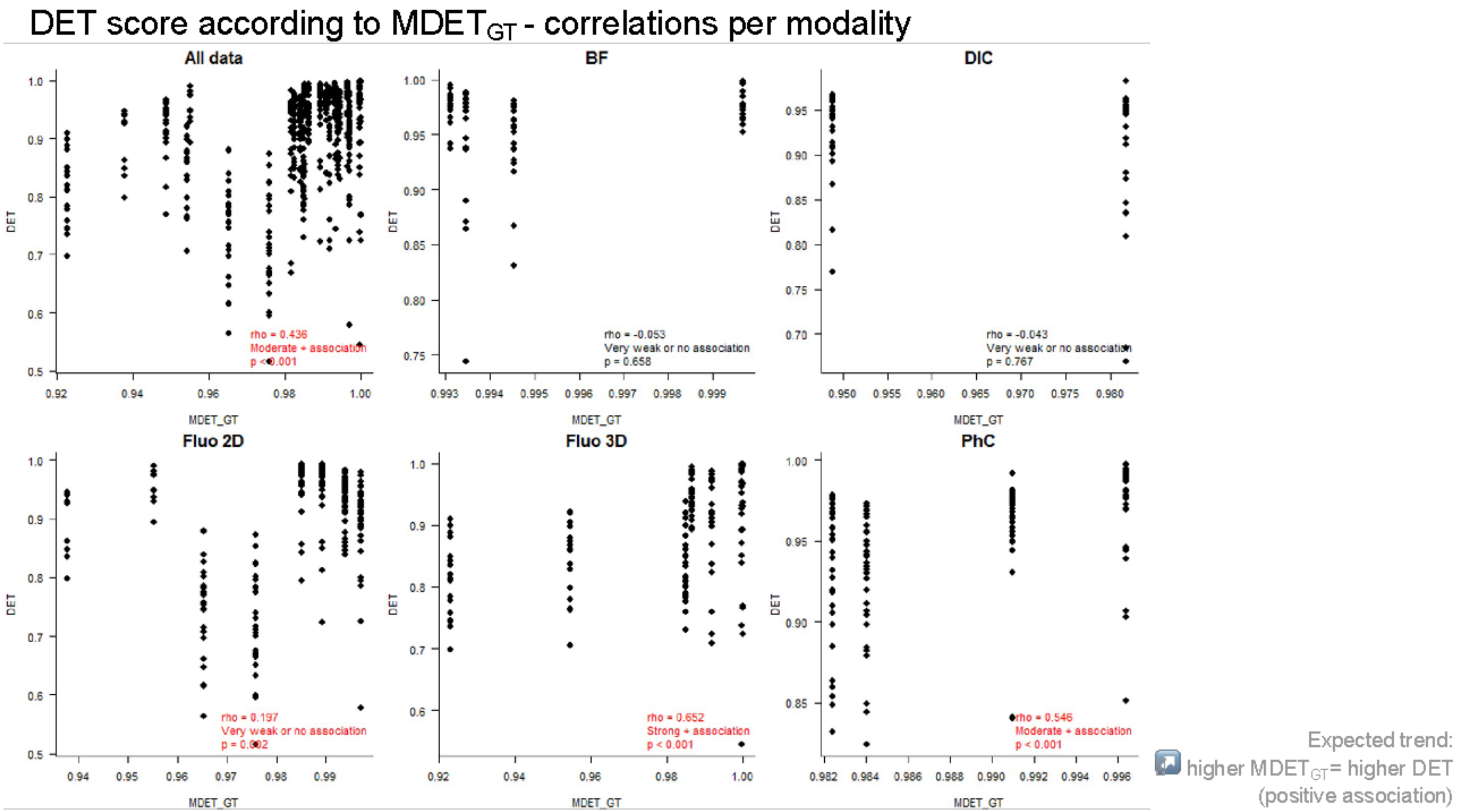

Supplementary Figure 44. Spearman's rank correlation coefficient between Detection scores and the quality of the GT Detection annotation ($MDET_{GT}$). Significance level fixed at 0.05. Measurements given per video sequence

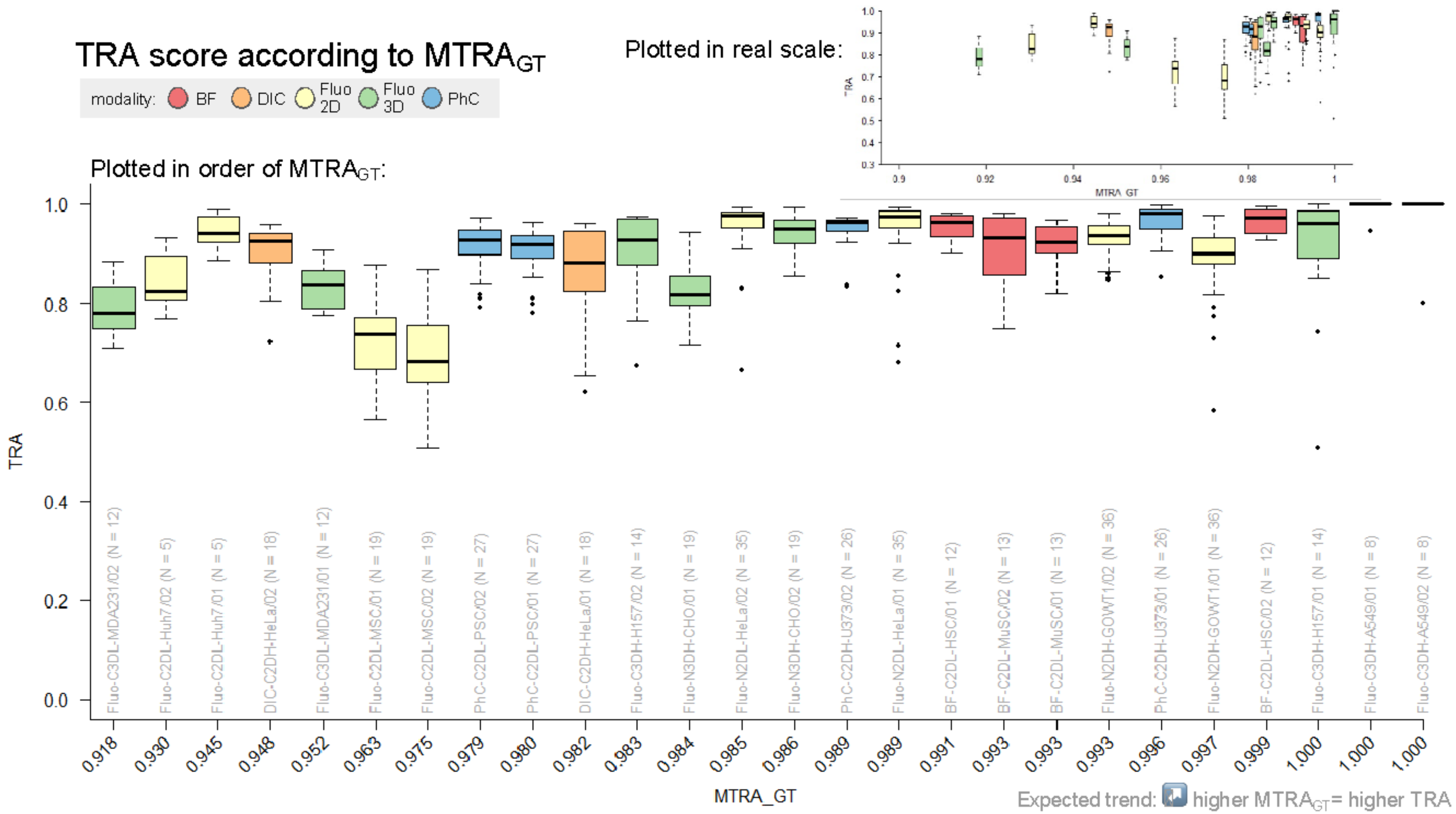

Supplementary Figure 45. Tracking scores as a function of the quality of the GT Tracking annotation (MTRA$_{GT}$). Bold line represents median values. Measurements are given per video sequence. Outliers indicate values higher/lower than 1.5 times the interquartile range.

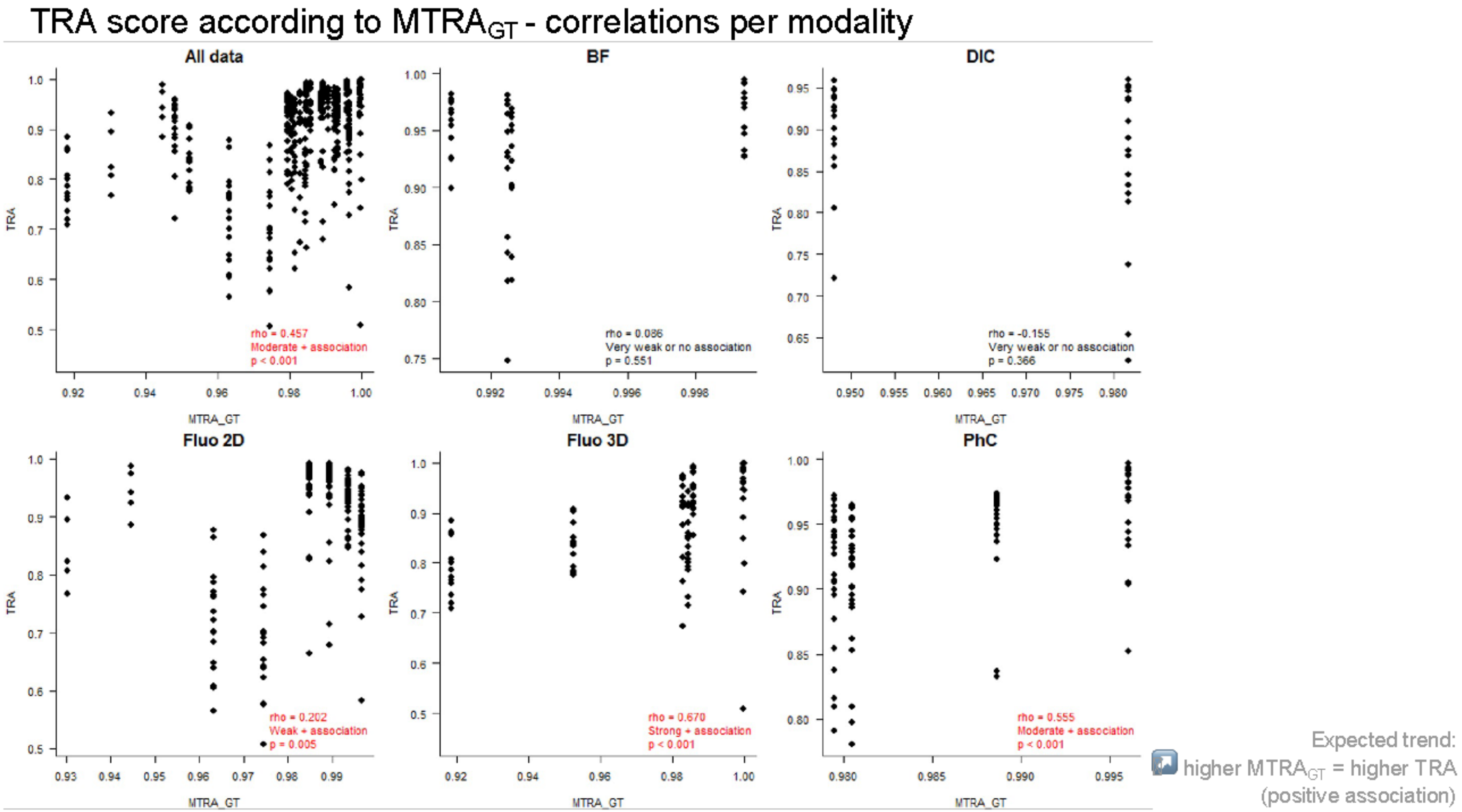

Supplementary Figure 46. Spearman's rank correlation coefficient between Tracking scores and the quality of the GT Tracking annotation ($MTRA_{GT}$). Significance level fixed at 0.05. Measurements given per video sequence.

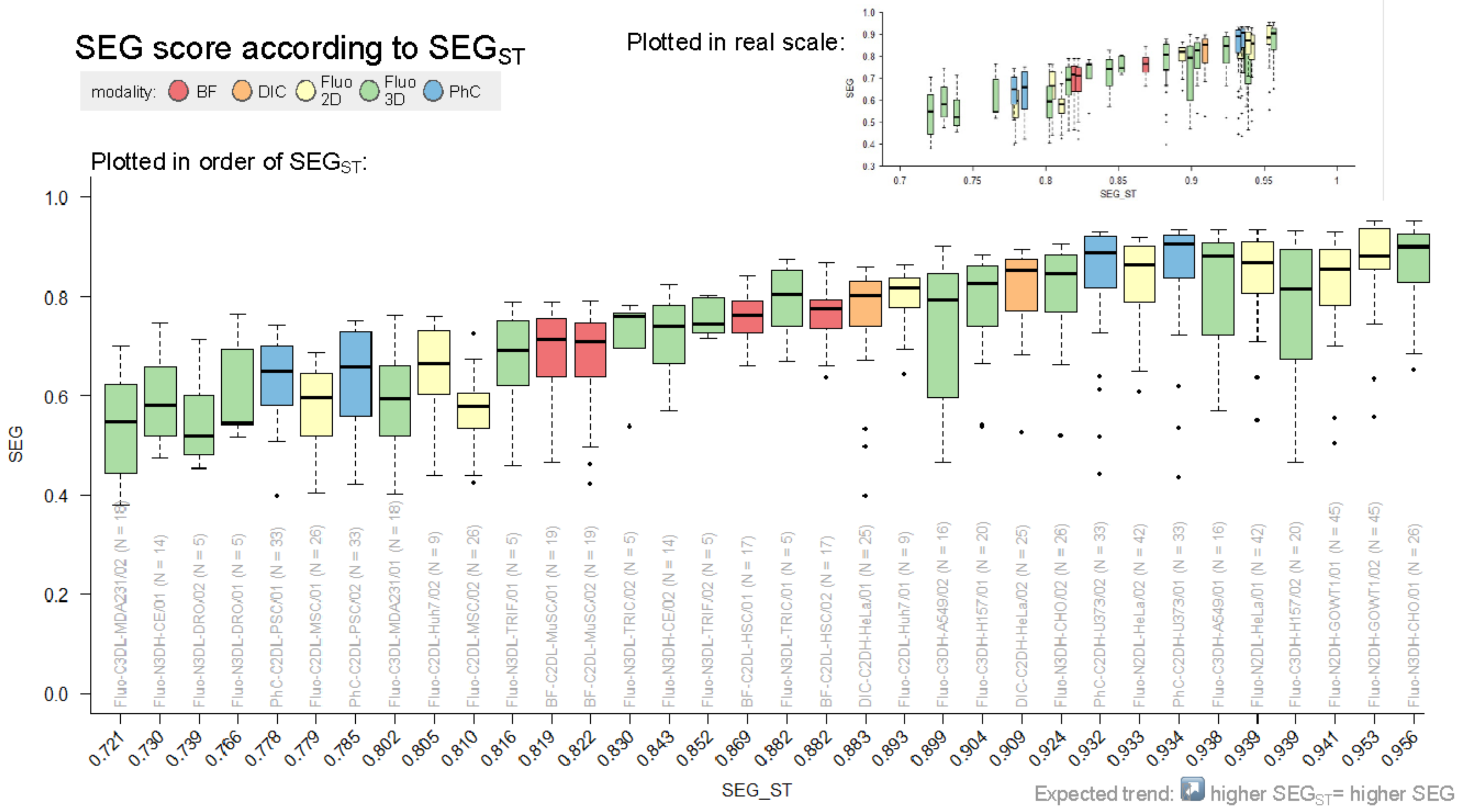

Supplementary Figure 47. Segmentation scores as a function of the quality of the ST Segmentation annotation (MSEG$_{ST}$) Bold line represents median values. Measurements are given per video sequence. Outliers indicate values higher/lower than 1.5 times the interquartile range.

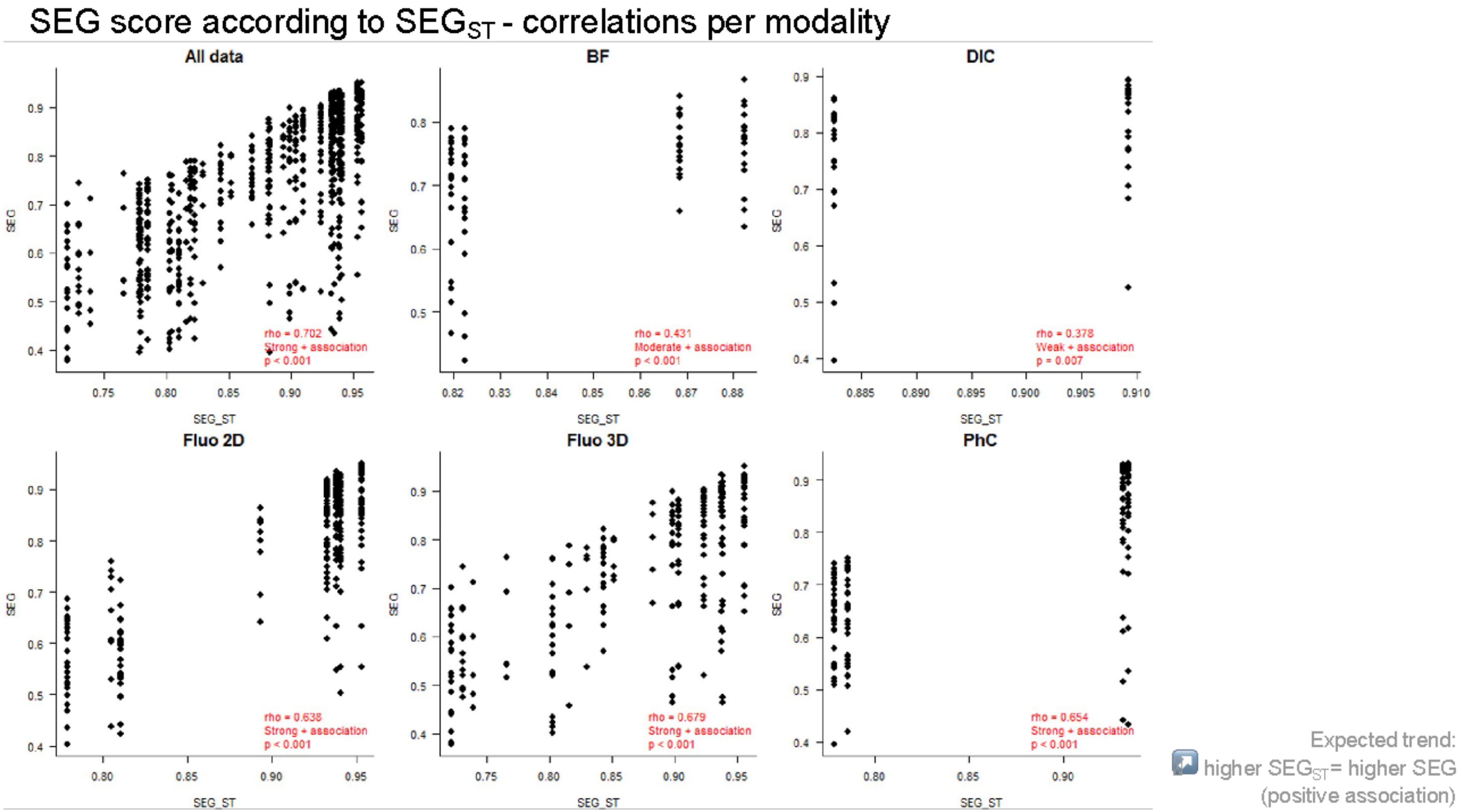

Supplementary Figure 48. Spearman's rank correlation coefficient between Segmentation scores and the quality of the ST Segmentation annotation ($MSEG_{ST}$). Significance level fixed at 0.05. Measurements given per video sequence.

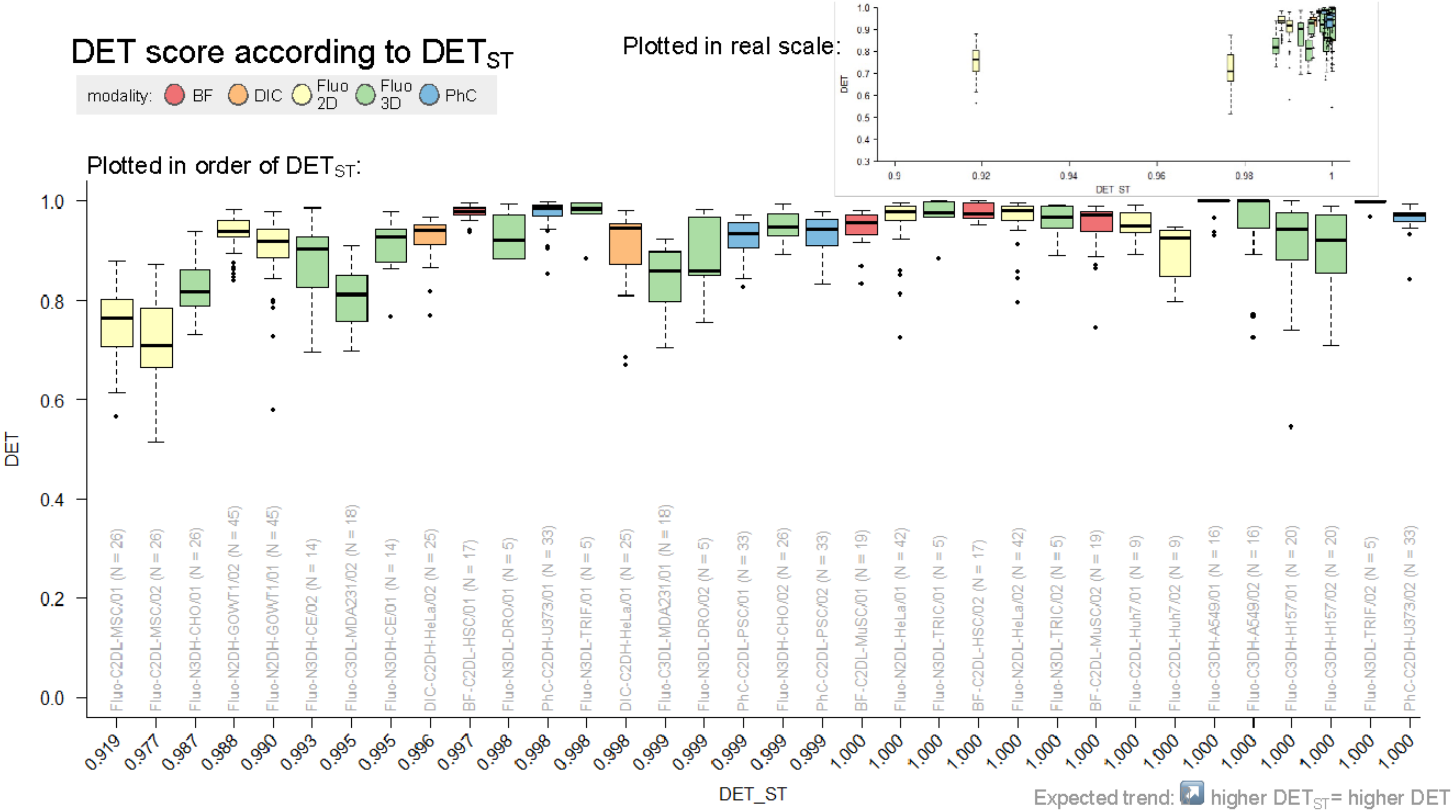

Supplementary Figure 49. Detection scores as a function of the quality of the ST Detection annotation (MDET$_{ST}$). Bold line represents median values. Measurements are given per video sequence. Outliers indicate values higher/lower than 1.5 times the interquartile range

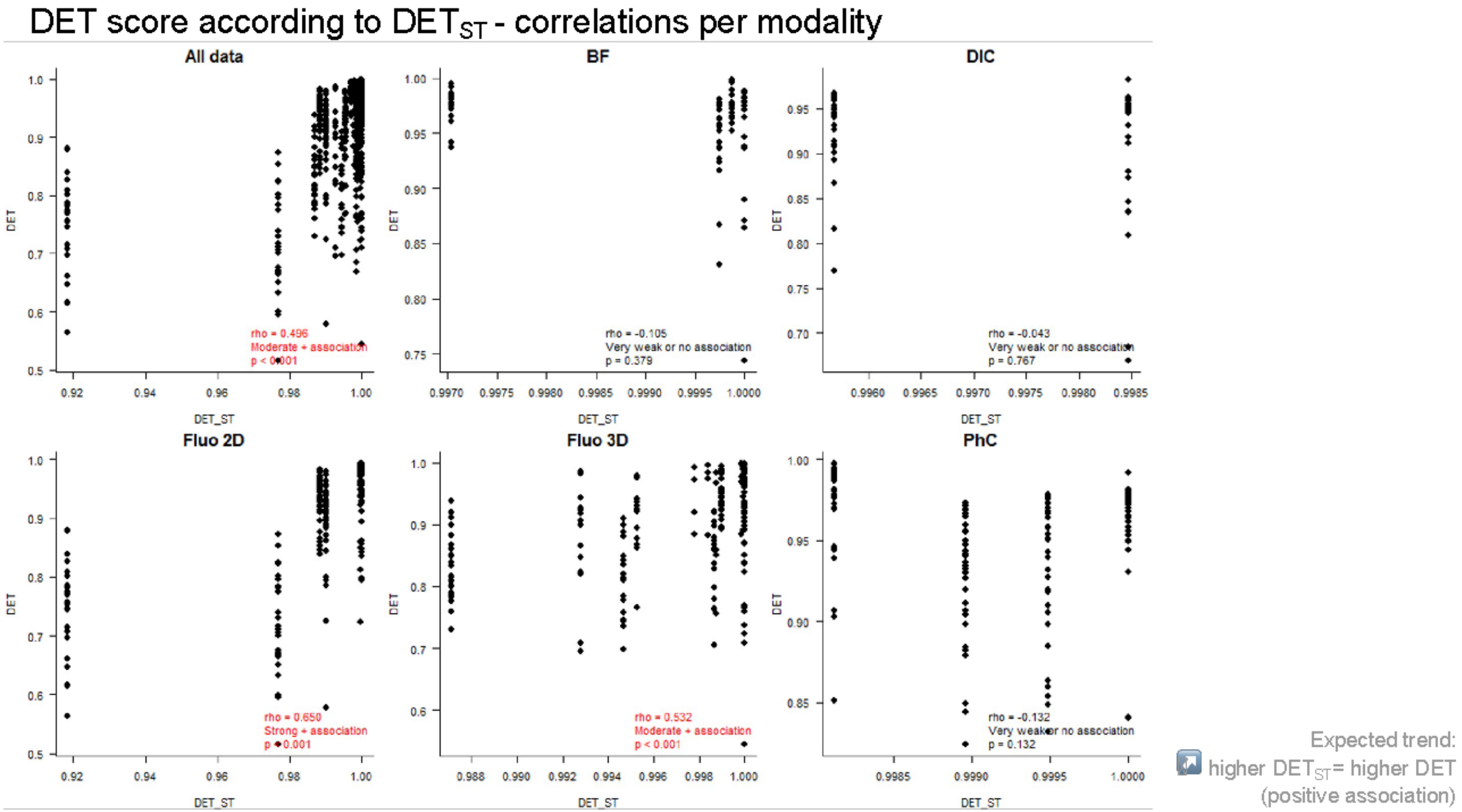

Supplementary Figure 50. Spearman's rank correlation coefficient between Detection scores and the quality of the ST Detection annotation (MDET$_{ST}$). Significance level fixed at 0.05. Measurements given per video sequence.

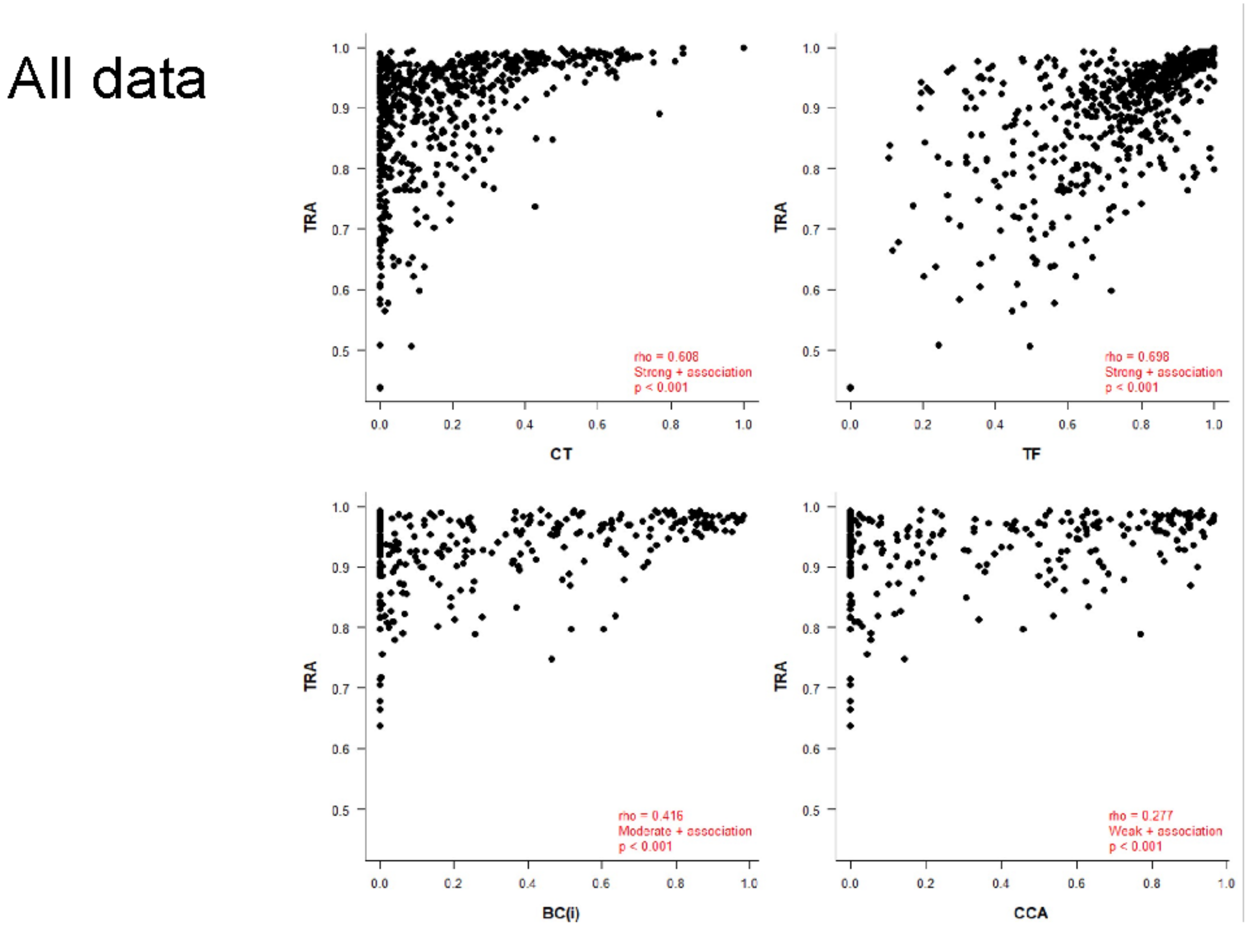

Supplementary Figure 51. Spearman's rank correlation coefficient between the Tracking scores and the biologically inspired measures, for all datasets. Significance level fixed at 0.05. Measurements given per video sequence. Complete tracks (CT), Track fractions (TF), Branching Correctness (BC(i)), Cell cycle accuracy (CCA)

BF

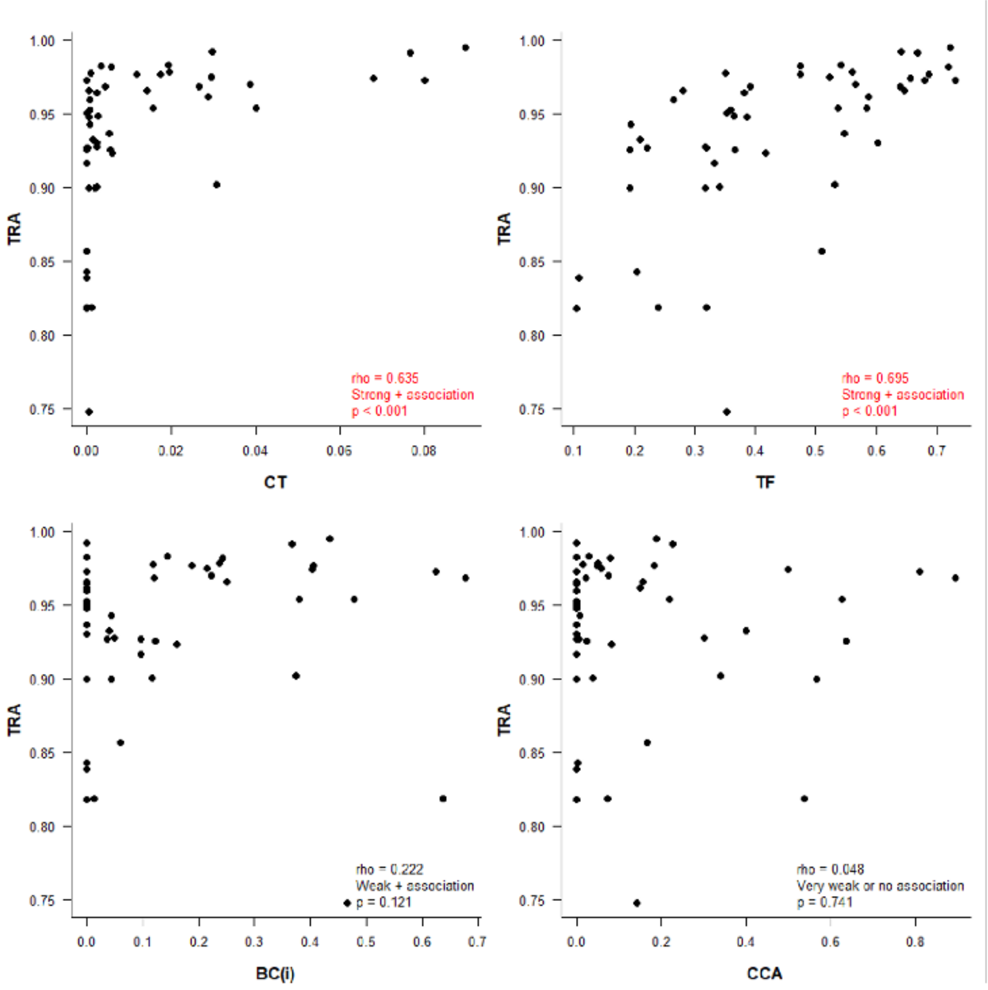

Supplementary Figure 52. Spearman's rank correlation coefficient between the Tracking scores and the biologically inspired measures, for brightfield (BD) datasets. Significance level fixed at 0.05. Measurements given per video sequence. Complete tracks (CT), Track fractions (TF), Branching Correctness (BC(i)), Cell cycle accuracy (CCA)

DIC

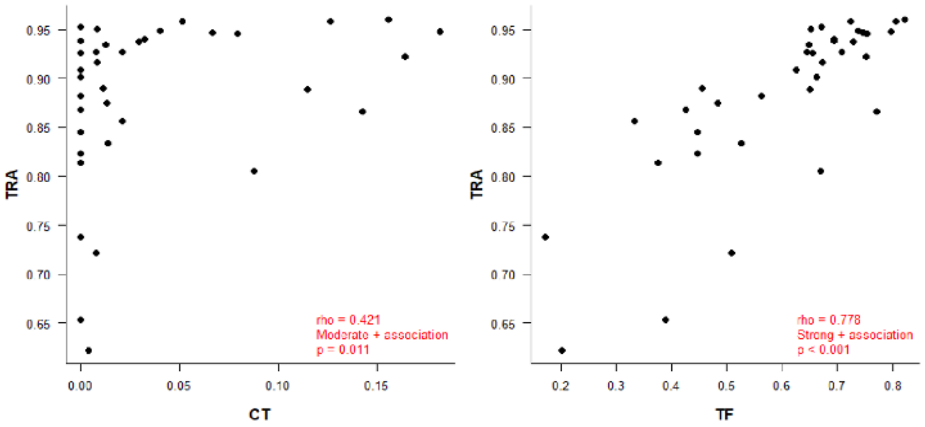

BC(i): no values are available

CCA: no values are available

Supplementary Figure 53. Spearman's rank correlation coefficient between the Tracking scores and the biologically inspired measures, for Differential Interference Contrast (DCI) datasets. Significance level fixed at 0.05. Measurements given per video sequence. Complete tracks (CT), Track fractions (TF), Branching Correctness (BC(i)), Cell cycle accuracy (CCA)

Fluo 2D

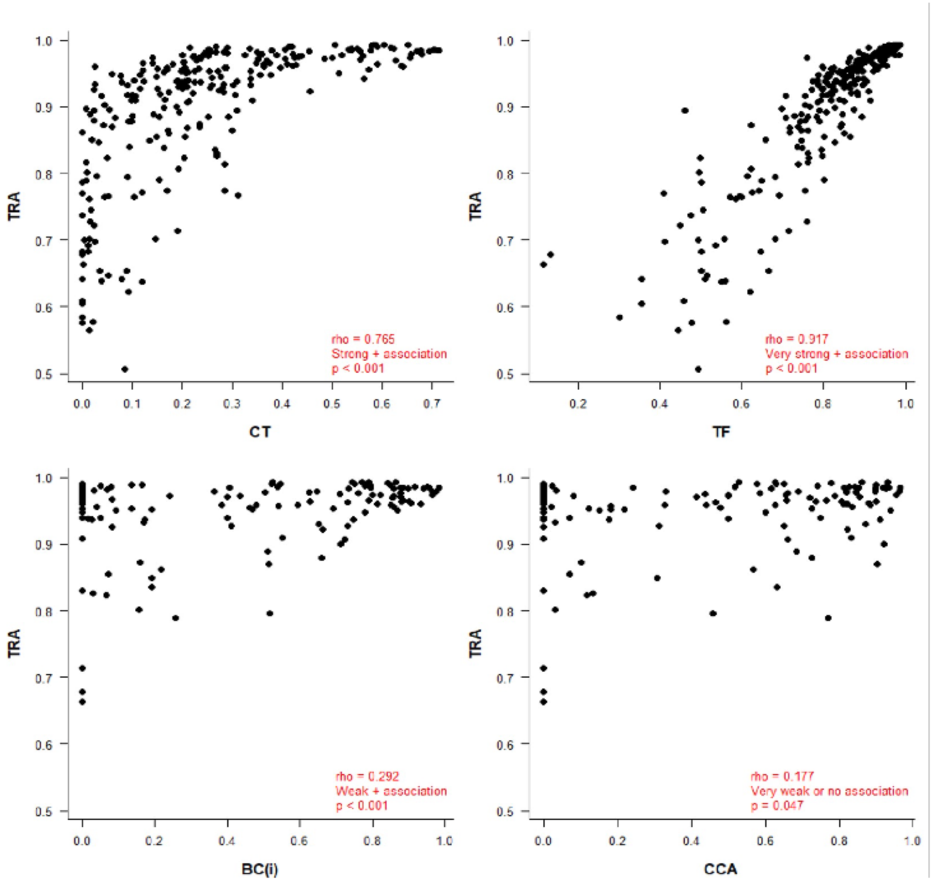

Supplementary Figure 54. Spearman's rank correlation coefficient between the Tracking scores and the biologically inspired measures, for 2D Fluorescence (Fluo 2D) datasets. Significance level fixed at 0.05. Measurements given per video sequence. Complete tracks (CT), Track fractions (TF), Branching Correctness (BC(i)), Cell cycle accuracy (CCA

Fluo 3D

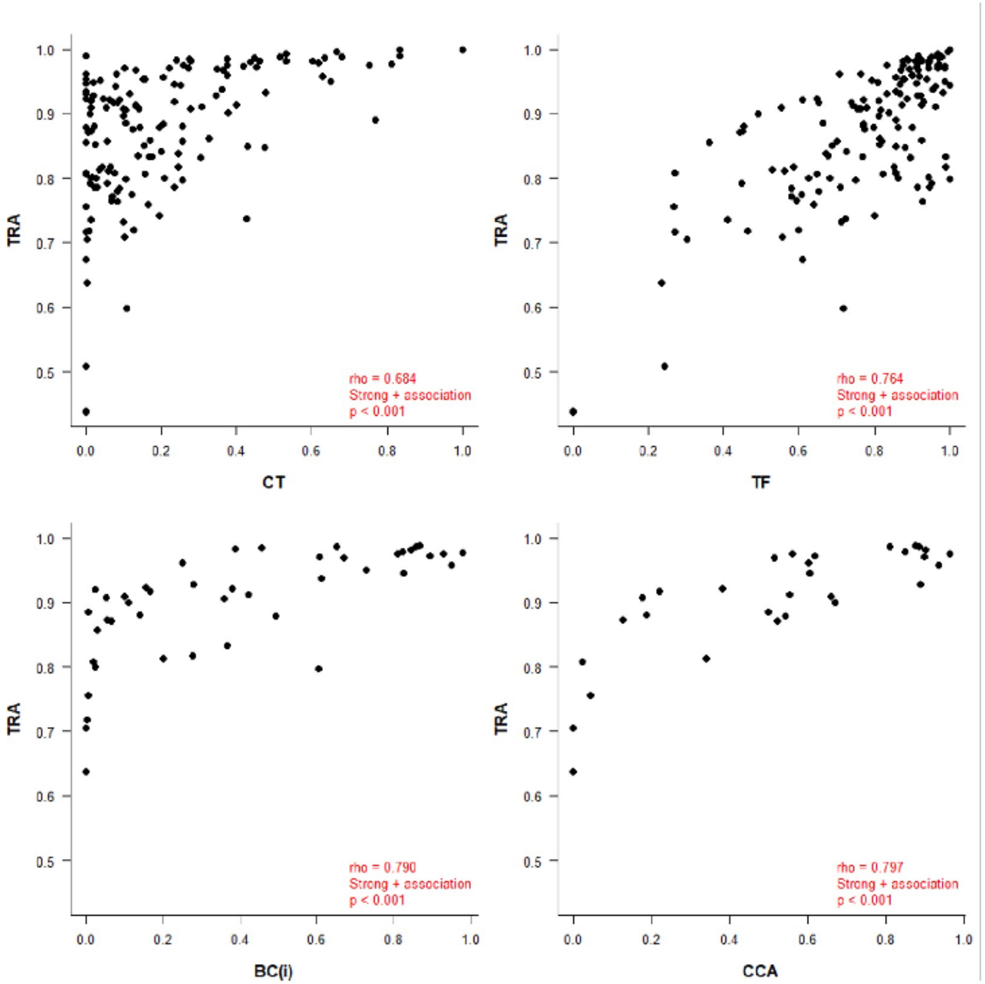

Supplementary Figure 55. Spearman's rank correlation coefficient between the Tracking scores and the biologically inspired measures, for 3D Fluorescence (Fluo 3D) datasets. Significance level fixed at 0.05. Measurements given per video sequence. Complete tracks (CT), Track fractions (TF), Branching Correctness (BC(i)), Cell cycle accuracy (CCA)

PhC

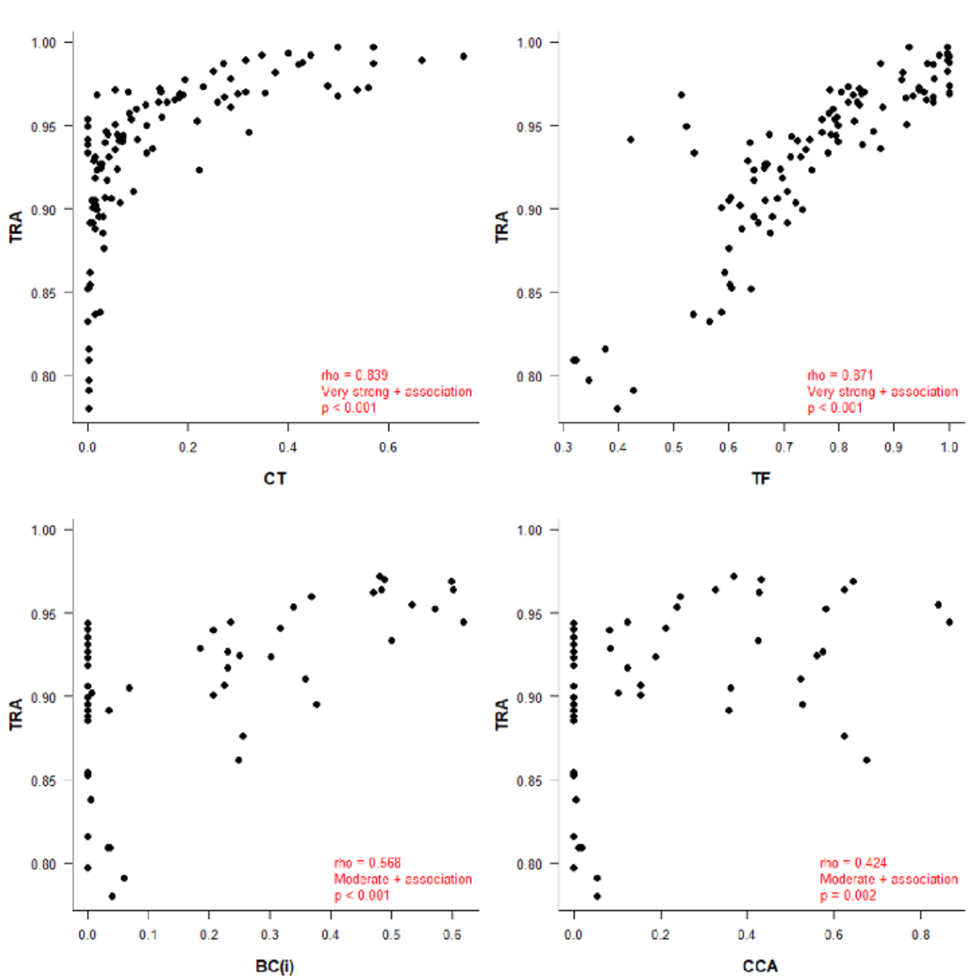

Supplementary Figure 56. Spearman's rank correlation coefficient between the Tracking scores and the biologically inspired measures, for Phase Contrast Microscopy (PhC) datasets. Significance level fixed at 0.05. Measurements given per video sequence. Complete tracks (CT), Track fractions (TF), Branching Correctness (BC(i)), Cell cycle accuracy (CCA)
