# Supplementary material for: The Cell Tracking Challenge: 10 years of objective benchmarking
Source: Nat Methods. 2023 May 18;20(7):1010–20. doi: 10.1038/s41592-023-01879-y (PMC10333123; doi:10.1038/s41592-023-01879-y)
Supplement: Supplementary file 2 — Reporting Summary [file 41592_2023_1879_MOESM2_ESM.pdf]

nature research

Corresponding author(s): Michal Kozubek, Carlos Ortiz de Solórzano

Last updated by author(s): Mar 31, 2023

Reporting Summary

Nature Research wishes to improve the reproducibility of the work that we publish. This form provides structure for consistency and transparency in reporting. For further information on Nature Research policies, see our Editorial Policies and the Editorial Policy Checklist.

Statistics

For all statistical analyses, confirm that the following items are present in the figure legend, table legend, main text, or Methods section.

n/a	Confirmed	
☐	☒	The exact sample size (*n*) for each experimental group/condition, given as a discrete number and unit of measurement
☐	☒	A statement on whether measurements were taken from distinct samples or whether the same sample was measured repeatedly
☐	☒	The statistical test(s) used AND whether they are one- or two-sided<br>*Only common tests should be described solely by name; describe more complex techniques in the Methods section.*
☐	☒	A description of all covariates tested
☐	☒	A description of any assumptions or corrections, such as tests of normality and adjustment for multiple comparisons
☐	☒	A full description of the statistical parameters including central tendency (e.g. means) or other basic estimates (e.g. regression coefficient) AND variation (e.g. standard deviation) or associated estimates of uncertainty (e.g. confidence intervals)
☐	☒	For null hypothesis testing, the test statistic (e.g. *F*, *t*, *r*) with confidence intervals, effect sizes, degrees of freedom and *P* value noted<br>*Give P values as exact values whenever suitable.*
☒	☐	For Bayesian analysis, information on the choice of priors and Markov chain Monte Carlo settings
☒	☐	For hierarchical and complex designs, identification of the appropriate level for tests and full reporting of outcomes
☐	☒	Estimates of effect sizes (e.g. Cohen's *d*, Pearson's *r*), indicating how they were calculated

Our web collection on statistics for biologists contains articles on many of the points above.

Software and code

Policy information about availability of computer code

Data collection	N/A No special software was used for data collection.
Data analysis	All the code used to produce the results reported in this article is freely available both at the CTC website and also as a Fiji plugin whose source codes can be found at https://github.com/CellTrackingChallenge/. Furthermore, this public github repository contains links to the executable versions of the individual algorithms and Colab Notebooks of those ten participants who agreed to share their tools. The parameters used by the participants to produce their submitted results are listed on the CTC website as well.<br>Analysis of the collected data was performed using R Statistical Software (v3.4.3).

For manuscripts utilizing custom algorithms or software that are central to the research but not yet described in published literature, software must be made available to editors and reviewers. We strongly encourage code deposition in a community repository (e.g. GitHub). See the Nature Research guidelines for submitting code & software for further information.

Data

Policy information about availability of data

All manuscripts must include a data availability statement. This statement should provide the following information, where applicable:

- Accession codes, unique identifiers, or web links for publicly available datasets
- A list of figures that have associated raw data
- A description of any restrictions on data availability

The training datasets with their reference annotations and test datasets used in the challenge are publicly available at the CTC website: http://celltrackingchallenge.net. As for the raw data for individual Figures and Tables:
Figure 1: Complete raw data available for download in the CTC website: http://celltrackingchallenge.net/datasets/. Source raw files used in the figure provided as Source Data Figures 1a, 1b, 1c, 1d, 1e, 1f, 1g

Figure 2: Calculated from raw test image data available for download on the CTC website: http://celltrackingchallenge.net/datasets/, from unreleased results of selected benchmarked algorithms, and from the secret tracking GT annotations, following the methodology described in the Dataset properties section in Online Methods and using the freely available Fiji plugins.
Figure 3: Compiled from the information contained in the CTC website (http://celltrackingchallenge.net/participants/), summarized in Supplementary Data (Tabs 3-4).
Figure 4: Compiled from the data contained in Supplementary Data (Tabs 5-9) & (Tabs 10-14).
Figure 5: Calculated from the data obtained for Figure 2 and from secret manual GT annotations of test images, following the methodology described in the Section Quality of annotations and human level performance section in Online Methods and using the freely available Fiji plugins.
Figure 6: Compiled from the data contained in Supplementary Data (Tabs 15-20).
Extended Data Figure 1: Compiled from the data contained in Supplementary Data (Tabs 3-4), (Tabs 5-9) & (Tabs 10-14).
Extended Data Figure 2: Compiled from the data contained in Supplementary Data (Tabs 3-4) & (Tabs 5-9).
Extended Data Figure 3: Compiled from the data contained in Supplementary Data (Tabs 3-4) & (Tabs 5-9).
Extended Data Figure 4: Compiled from the data contained in Supplementary Data (Tabs 3-4) & (Tabs 5-9).
Extended Data Figure 5: Compiled from the data contained in Supplementary Data (Tabs 3-4) & (Tabs 5-9).
Extended Data Figure 6: Compiled from the data contained in Supplementary Data (Tabs 3-4) & (Tabs 10-14).
Extended Data Figure 7: Compiled from the data contained in Supplementary Data (Tabs 3-4) & (Tabs 10-14).
Extended Data Figure 8: Compiled from the data contained in Supplementary Data (Tabs 3-4) & (Tabs 15-20).
Extended Data Figure 9: Compiled from the data contained in Supplementary Data (Tabs 21-33) & (Tabs 34-42).

Field-specific reporting

Please select the one below that is the best fit for your research. If you are not sure, read the appropriate sections before making your selection.

☒ Life sciences ☐ Behavioural & social sciences ☐ Ecological, evolutionary & environmental sciences

For a reference copy of the document with all sections, see nature.com/documents/nr-reporting-summary-flat.pdf

Life sciences study design

All studies must disclose on these points even when the disclosure is negative.

Sample size	N/A. This manuscript reports on procedures and software methods used to analyze images, not the actual use of those methods. There were no formal hypothesis either expected results in advance, therefore we collected the possible highest sample size regarding to the capacities.
Data exclusions	N/A (see comment about Sample size). Not to spoil the conclusions of the analyses by low-performing or poorly fine-tuned CTC submissions, only those algorithms that simultaneously achieved DET and SEG scores in case of the CSB submissions, and SEG and TRA scores in case of the CTB submissions, over halves of the reported human performance were considered.
Replication	N/A (see comment about Sample size) We did not perform any replication to have the groups independent as much as possible.
Randomization	N/A (see comment about Sample size) There was no randomization as we have no concerns about biased results due to chosen techniques.
Blinding	N/A (see comment about Sample size) There was no blinding as the participants chose the technique by their own to get the best results.

Reporting for specific materials, systems and methods

We require information from authors about some types of materials, experimental systems and methods used in many studies. Here, indicate whether each material, system or method listed is relevant to your study. If you are not sure if a list item applies to your research, read the appropriate section before selecting a response.

Materials & experimental systems

n/a	Involved in the study	
☒	☐	Antibodies
☒	☐	Eukaryotic cell lines
☒	☐	Palaeontology and archaeology
☒	☐	Animals and other organisms
☒	☐	Human research participants
☒	☐	Clinical data
☒	☐	Dual use research of concern

Methods

n/a	Involved in the study	
☒	☐	ChIP-seq
☒	☐	Flow cytometry
☒	☐	MRI-based neuroimaging
