# Supplementary figures and images for: The Cell Tracking Challenge: 10 years of objective benchmarking
Source: Nat Methods. 2023 May 18;20(7):1010–20. doi: 10.1038/s41592-023-01879-y (PMC10333123; doi:10.1038/s41592-023-01879-y)

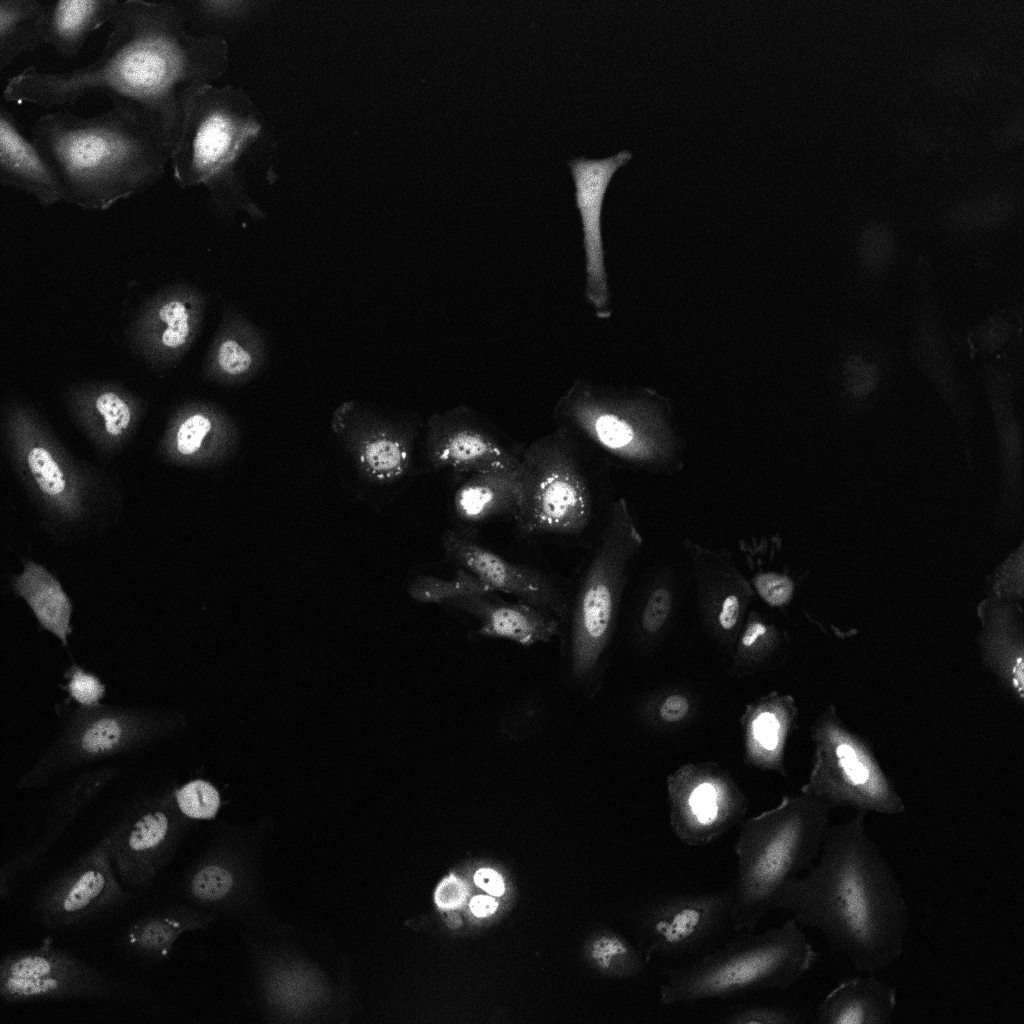

Supplement: Source Data Fig. 1 — Original image frames used in each of the seven parts (a–g) of Fig. 1. [file 41592_2023_1879_MOESM5_ESM.zip › Source-Data-File-Figure 1/Figure_1a.tif]

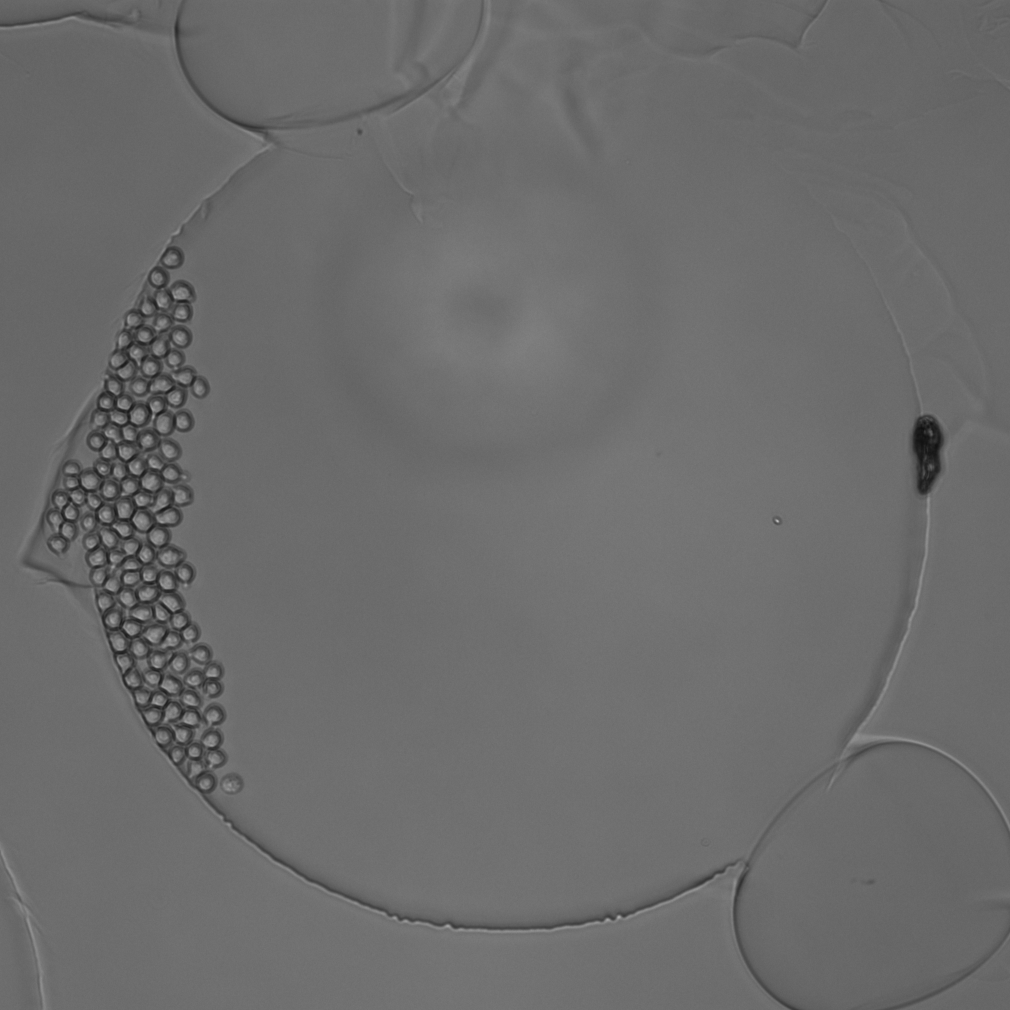

Supplement: Source Data Fig. 1 — Original image frames used in each of the seven parts (a–g) of Fig. 1. [file 41592_2023_1879_MOESM5_ESM.zip › Source-Data-File-Figure 1/Figure_1b.tif]

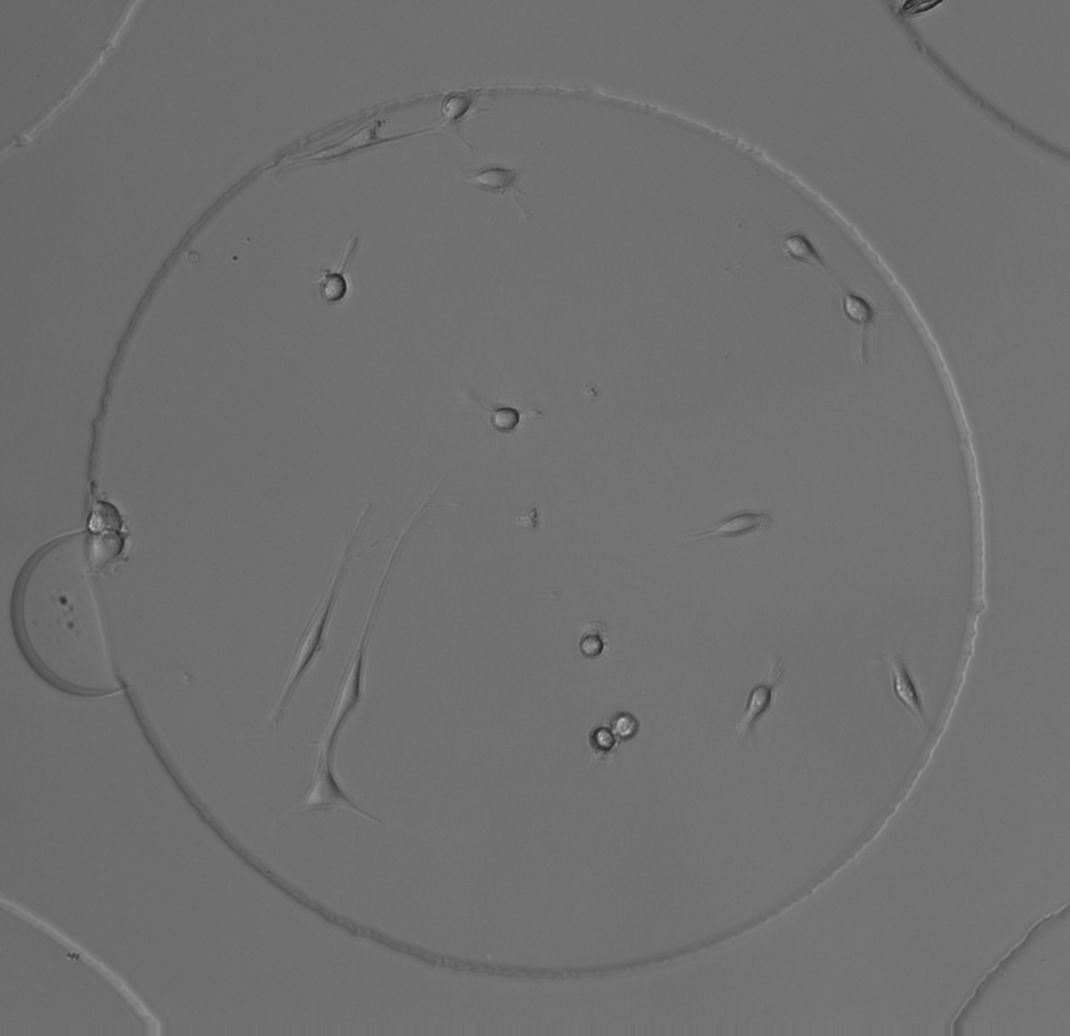

Supplement: Source Data Fig. 1 — Original image frames used in each of the seven parts (a–g) of Fig. 1. [file 41592_2023_1879_MOESM5_ESM.zip › Source-Data-File-Figure 1/Figure_1c.tif]

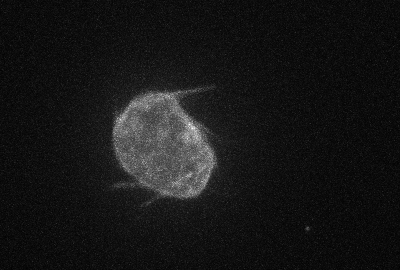

Supplement: Source Data Fig. 1 — Original image frames used in each of the seven parts (a–g) of Fig. 1. [file 41592_2023_1879_MOESM5_ESM.zip › Source-Data-File-Figure 1/Figure_1d.tif]

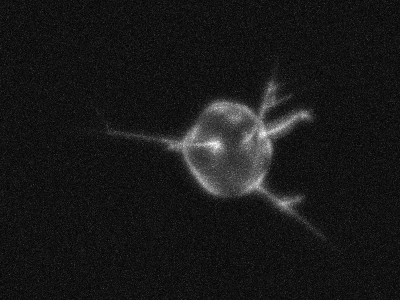

Supplement: Source Data Fig. 1 — Original image frames used in each of the seven parts (a–g) of Fig. 1. [file 41592_2023_1879_MOESM5_ESM.zip › Source-Data-File-Figure 1/FIgure_1e.tif]

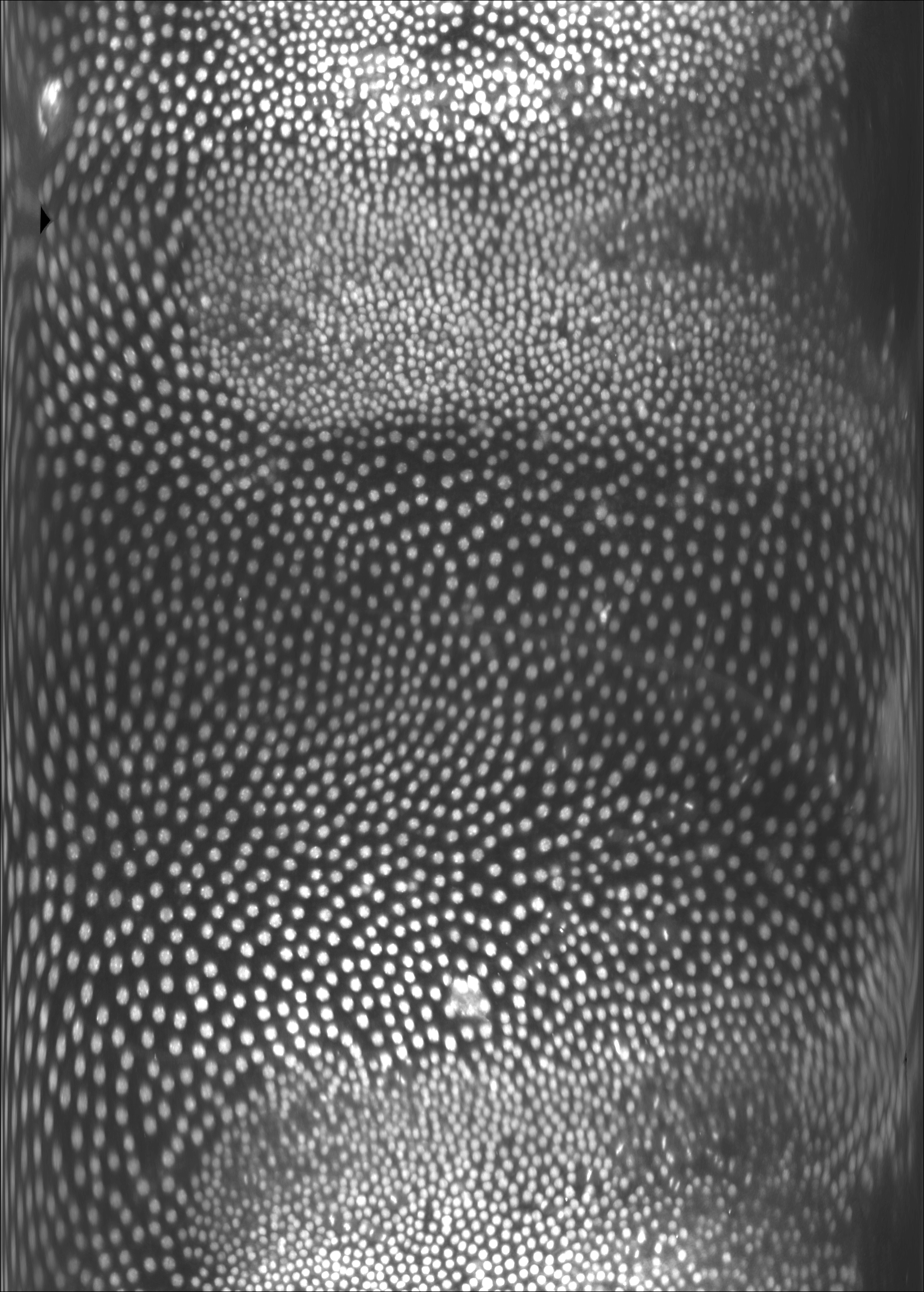

Supplement: Source Data Fig. 1 — Original image frames used in each of the seven parts (a–g) of Fig. 1. [file 41592_2023_1879_MOESM5_ESM.zip › Source-Data-File-Figure 1/Figure_1f.tif]

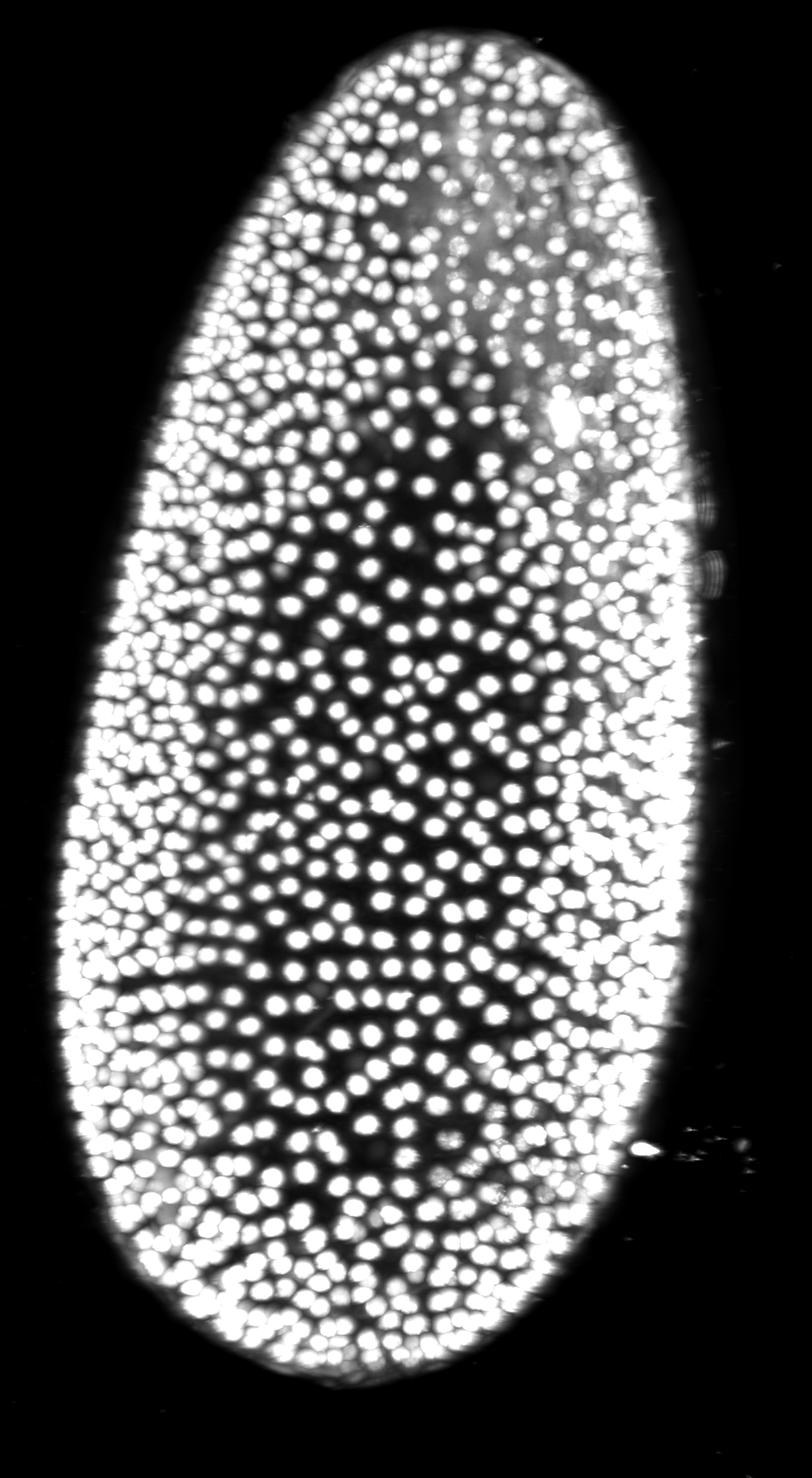

Supplement: Source Data Fig. 1 — Original image frames used in each of the seven parts (a–g) of Fig. 1. [file 41592_2023_1879_MOESM5_ESM.zip › Source-Data-File-Figure 1/Figure_1g.tif]
